# Supplementary material for: The human SKI complex regulates channeling of ribosome-bound RNA to the exosome via an intrinsic gatekeeping mechanism
Source: Mol Cell. 2022 Feb 17;82(4):756–769.e8. doi: 10.1016/j.molcel.2022.01.009 (PMC8860381; doi:10.1016/j.molcel.2022.01.009)
Supplement: Document S1. Figures S1–S7 and Tables S1 and S2 [file mmc1.pdf]

Molecular Cell, Volume *82*

Supplemental information

The human SKI complex regulates channeling of ribosome-bound RNA to the exosome via an intrinsic gatekeeping mechanism

Alexander Kögel, Achim Keidel, Fabien Bonneau, Ingmar B. Schäfer, and Elena Conti

Supplementary Figure S1. Cryo-EM data analysis of recombinant apo hSKI (related to Figures 1, 2 and 3)

Characteristics of the apo hSKI single particle cryo-EM sample and data. **(A) On the left,** analytical size exclusion chromatography profile on a S6 increase column (Cytiva) of a representative hSKI preparation, and on the right is a 4-12 % Coomassie stained SDS-PAGE of a peak fraction of the SEC run (labelled with *, M is Marker). In **(B)** a representative cryo-EM micrograph of the apo hSKI sample (A) is shown. This image was recorded at 300 kV with a pixel size of 1.024 Å/pix using a post-GIF K3 direct detector. **(C)** 2D class averages of the apo hSKI sample in the closed (left hand panel, red frame) and open (right hand panel, blue frame) state conformations. **(D)** Processing scheme of the single particle cryo-EM dataset of the apo hSKI sample resulting in 3D reconstructions for the closed (red frame) and open (blue frame) states.

Supplementary Figure S2. Cryo-EM reconstruction analysis of apo hSKI (related to Figures 1, 2 and 3)

Quality indicators of the apo hSKI closed and open 3D reconstructions. Local resolution estimates and Fourier Shell Correlations (FSC) of the closed **((A)** and **(B))** and the open **((D)** and **(E))** reconstructions of the apo hSKI cryo-EM SPA data. The FSC of the masked independent half maps were calculated in the RELION 3.1 postprocessing routine and the map vs model FSC using phenix.mtriage. The FSC cut-off criteria of 0.5 and 0.143 are indicated by dotted lines. Angular sampling distributions of the closed **(C)** and open **(F)** reconstructions of the apo hSKI data. Sampling angle data were plotted in 3º by 3º bins and sampling bins coloured according to particle number with red indicating more and blue fewer particles. **(G)** Selected model and map regions of the closed state reconstructions showing the quality of the structural data. **(H)** Local resolution estimate of a focused 3D reconstruction of the $hSKI2_{arch}$ (panel on the left) used for rigid fitting of a homology model of this domain (panel on the right) and completion of the closed state model.

Supplementary Figure S3. Structural conservation between human SKI and yeast Ski (related to Figures 2, 3, 5 and 6)

Comparisons of the hSKI apo and the *S. cerevisiae* Ski structures. In **(A)** the model of hSKI closed apo state is juxtaposed to the yeast Ski model. SKI2 is coloured in yellow,

SKI3 in blue, $SKI8_{IN}$ in green and $SKI8_{OUT}$ in dark green. The SKI3 C-terminal module is absent in the yeast structure (box (B)). **(B)** Detailed view of the C-terminal module of hSKI3 in complex with the N-terminus of the hSKI2. **(C)** The hSKI2 N-terminal outer a-helix is sandwiched between hSKI3 TPR13 and TPR14. Note that the residue numbering of the $Ski2_N$ a-helix (see also Figure 2C) is based on prediction from AlphaFold (Tunyasuvunakool et al., 2021). A focused 3D reconstruction on the first superhelical turn of hSKI3 in the hSKI apo closed state is coloured according to local resolution on the left and displayed together with the respective part of the model on the right. **(D)** hSKI2 helicase modules of the apo hSKI closed state, the RNA-bound hSKI closed state and the *S.c.* Ski structure in comparison. Note the similar orientation and conformation of the $SKI2_{arch}$ with respect to the $SKI2_{cat}$ in all three structures. **(E)** Comparison of the human and the yeast 80S bound by human and yeast SKI complex respectively (*S. cerevisiae* 80S-Ski complex from PDB:5MC6). SKI2 is coloured in yellow, SKI3 in blue, $SKI8_{IN}$ in green, $SKI8_{OUT}$ in dark green, the CrPV IRES in the human case and the mRNA and tRNA in the yeast case in red, the 60S ribosomal subunit is in light blue and the 40S ribosomal subunit in light orange. The yeast and human SKI complexes sit in different orientations on the respective 80S and the RNAs appear differently bound to the helicase modules. In the yeast case this leaves 2-3 nucleotide positions to ySki2 R149 unoccupied (ySki2 R149 is structurally in the equivalent position as hSKI2 W146).

Supplementary Figure S4. SKI2 elements impacting the open/closed state (related to Figure 2, 3 and 4)

Details of selected elements in the $hSKI2_N$. In **(A)** a multiple sequence alignment (MSA) of eukaryotic SKI2 homologs focussed on the N-terminal wedge and a schematic representation of this region in the hSKI2 construct used for the hSKI-Δwedge complex is plotted. Degrees of conservation are indicated by shades of yellow in the MSA with darker shades highlighting stronger conservation. The organisation of the different motives of interest in proximity to the hSKI2 wedge is indicated in the cartoon atop the MSA by thicker yellow lines. A Coomassie stained SDS-PAGE of this sample is shown in Fig. 7B. These samples were vitrified on holey-carbon Cu grids and imaged at 200kV using a K2 direct detector in counting model at a pixel size of 1.885 Å/pix. In the top panel in **(B)** reference-free 2D class averages of these cryo-EM SPA data of the

hSKIΔwedge complex are displayed. These 2D class averages are overall similar to those of the apo open hSKI particles in Fig. S1C. The apo open hSKI complex was fitted into the low-resolution 3D reconstruction of the hSKIΔwedge complex in the panel at the bottom. The unmodelled density on the right with corkscrew-like appearance corresponds to the unmodelled hSKI3 N-terminal TPRs. Note the absence of any interpretable density for the hSKI2 helicase module. hSKI2 is coloured in yellow, hSKI3 in blue, $hSKI8_{IN}$ in green and $hSKI8_{OUT}$ in dark green. In single particle cryo-EM, a *S. cerevisiae* Ski-Δarch complex adopts an open and closed conformation. **(C)** Coomassie stained 4-12 % SDS-PAGE of the yeast SkiΔarch complex. These samples were vitrified on holey-carbon Cu grids and imaged at 200kV using a K2 direct detector operated as above. Reference-free 2D class averages and the final 3D reconstruction of the closed state of the yeast Ski-Δarch complex are in **(D)**. **(E)** shows reference-free 2D class averages and the final 3D reconstruction of the open state of the yeast Ski-Δarch complex. Ski2 is coloured in yellow, Ski3 in blue, $Ski8_{IN}$ in green and $Ski8_{OUT}$ in dark green. **(F)** Fourier shell correlation of the masked independent half-maps for the 3D reconstructions of hSKIΔwedge (shown in **(B)**, forest green) as well as the closed and open states of the yeast Ski-Δarch complex (shown in **(D)** and **(E),** coloured in salmon and olive, respectively)**.**

Supplementary Figure S5. Characteristics of the RNA-bound closed hSKI single particle cryo-EM data (related to Figure 4)

(A) shows a representative cryo-EM micrograph and reference-free 2D class averages of the RNA-bound closed hSKI sample recorded at 300 kV with a pixel size of 0.8512 Å/pix using a Gatan K3 camera. In **(B)** the processing scheme of the single particle cryo-EM dataset of the hSKI sample is outlined. This resulted in the final 3D reconstruction with a global resolution estimate of 3.1 Å for the closed RNA-bound state (red frame). The local resolution estimates are plotted on the final reconstruction in **(C)**, the FSC of the masked independent half-maps of the final reconstruction and the model vs map FSC in **(D)**. Here the FSC cut-off criteria of 0.5 and 0.143 are indicated by dotted lines. The angular sampling distribution for the final reconstruction is in **(E).** Angular sampling data were plotted in 3º by 3º bins in this panel. Note the absence of any noticeable fraction of open state particles in these SPA data (see 3D classification in **(B)**). Comparisons of the closed RNA-bound hSKI2 helicase with

hMTR4. In **(F)** RNA-bound human MTR4 in grey is superposed onto the closed RNA-bound hSKI2 helicase in yellow (human MTR4 from PDB:6D6Q). The RNA is coloured in red. Note the very similar path of the RNA in both structures. **(G)** Details of the RNA channel in the helicase module of the RNA-bound hSKI apo closed state similar to Fig. 4C. Residues interacting with the RNA are shown in stick representation. Colour scheme as in **(F)**. **(H)** Superposition of the RNA bound hSKI2 with human MTR4 bound to $ZCCHC8_{CTD}$. Residues W146 of the $hSKI2_{wedge}$ and F673 of the $ZCCHC8_{CTD}$ occupy very similar positions at one end of the RNA channel in the two structures (MTR4: $ZCCHC8_{CTD}$ from PDB:6C90). In the RNA-bound closed hSKI structures this residue closes off the RNA channel of the hSKI2 helicase at the 3' end prohibiting the RNA from further threading through.

Supplementary Figure S6. Characteristics of the closed ribosome-bound hSKI single particle cryo-EM sample and data (related to Figure 6)

(A) Typical 15-40 % (w/v) Sucrose gradient profile used for reconstitution of the closed state ADP-BeF-treated human 80S-IRES-hSKI complex on the left (80S-IRES-hSKI black curve; 80S-IRES grey curve). Peak fractions (indicated by *) were run out on a 4-12 % SDS-PAGE and the Coomassie stained gel is in the right panel. hSKI8 is not clearly identifiable on the gel most likely due to the prevalence of human 80S ribosomal proteins between 30 and 40 kDa. These peak fractions were vitrified and imaged at 300 kV using a post-GIF K3 camera with a pixel size of 0.8512 Å/pix **(B)**. Reference-free 2D class averages of the closed state 80S-IRES-hSKI sample are shown in **(C)**. Density for the hSKI complex is discernible atop the 40S ribosomal subunit in most of these 2D class averages. **(D)** displays the processing workflow for the closed ribosome-bound hSKI single particle cryo-EM data resulting, after subtraction of the 80S density, in a reconstruction of the hSKI complex at 3.6 Å resolution. The red masks indicate the regions of the map not subtracted from the data. The angular sampling distribution, the FSC of the independent masked half-maps and the map vs model FSC as well as the local resolution estimates plotted on the final closed ribosome-bound hSKI structure are shown in **(E)**, **(F)** and **(G)** respectively. In **(F)** dotted lines indicate the FSC cut-off criteria of 0.5 and 0.143.

Supplementary Figure S7. Characteristics of the open ribosome-bound hSKI single particle cryo-EM data (related to Figure 7)

(A) The purified protein samples used in the RNase protection assays in Figure 7B are shown on a Coomassie-stained 4-12 % SDS-PAGE. The samples are peak fractions from size-exclusion chromatography purifications. In **(B)** a representative micrograph (at a pixel size of 0.8512 Å/pix) and in **(C)** reference-free 2D class averages of the ATP-treated 80S-IRES-hSKI complex in open state are shown. In most 2D class averages density for the hSKI2 helicase at the top of the 40S ribosomal subunit is identifiable. Note the distinct structure of the $hSKI2_{arch}$ in these 2D class averages. **(D)** shows the local resolution estimates for the open ribosome bound hSKI structure from which the density of the 80S has been subtracted. In **(E)** angular sampling distribution for the reconstruction in **(D)** is plotted and in **(F)** the FSC for the masked independent half-maps and the model vs map for said reconstruction with dotted lines indicate the FSC cut-off criteria of 0.5 and 0.143. **(G)** shows the processing scheme with the red masks indicating the region of the map not subtracted from the data. After subtraction both 3D classifications with either apo closed hSKI or open ribosome-bound hSKI as reference model identify sub-populations of both the open and the closed hSKI state in these data. Of the ~110 000 particles identified initially as hSKI-bound, approximately 65% appear clearly in the open state in the second round of focused 3D classification using the open state as start model. The hSKI2 helicase is well ordered in these data since the 80S serves as an anchor for its substrate engagement. This is in contrast and somewhat paradoxical to the apo open hSKI state, where the helicase domain appears flexible in relation to the rest of the complex.

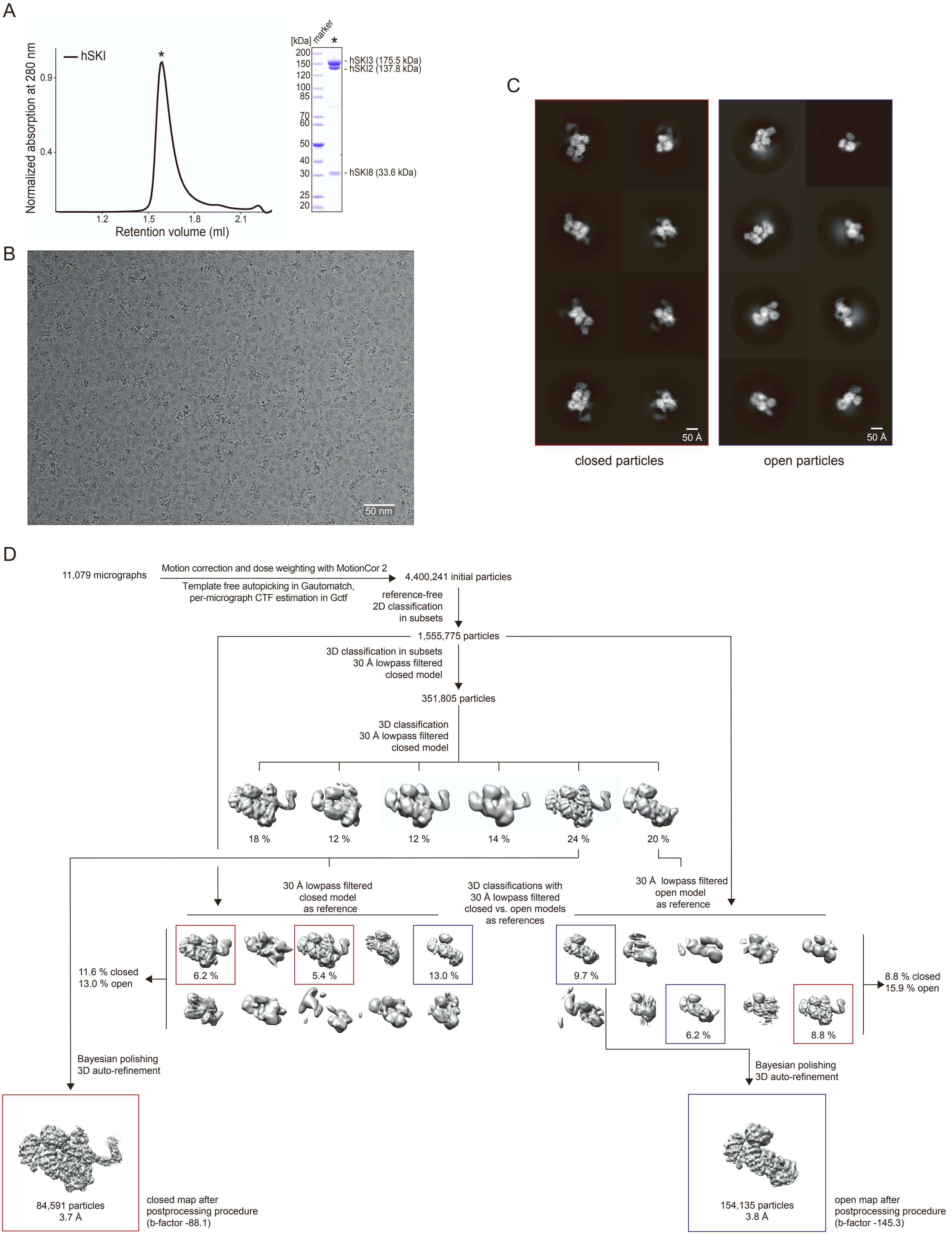
A
hSKI
Normalized absorption at 280 nm
0.9
0.4
1.2
1.5
1.8
2.1
Retention volume (ml)
[kDa]
marker
200
150
120
100
85
70
60
50
40
30
25
20
hSKI3 (175.5 kDa)
hSKI2 (137.8 kDa)
hSKI8 (33.6 kDa)
B
50 nm
C
50 Å
closed particles
open particles
D
11,079 micrographs
Motion correction and dose weighting with MotionCor 2
Template free autopicking in Gautomatch,
per-micrograph CTF estimation in Gctf
4,400,241 initial particles
reference-free
2D classification
in subsets
1,555,775 particles
3D classification in subsets
30 Å lowpass filtered
closed model
351,805 particles
3D classification
30 Å lowpass filtered
closed model
18 %
12 %
12 %
14 %
24 %
20 %
30 Å lowpass filtered
closed model
as reference
3D classifications with
30 Å lowpass filtered
closed vs. open models
as references
30 Å lowpass filtered
open model
as reference
6.2 %
5.4 %
13.0 %
11.6 % closed
13.0 % open
9.7 %
6.2 %
8.8 %
8.8 % closed
15.9 % open
Bayesian polishing
3D auto-refinement
84,591 particles
3.7 Å
closed map after
postprocessing procedure
(b-factor -88.1)
154,135 particles
3.8 Å
open map after
postprocessing procedure
(b-factor -145.3)

Figure S1

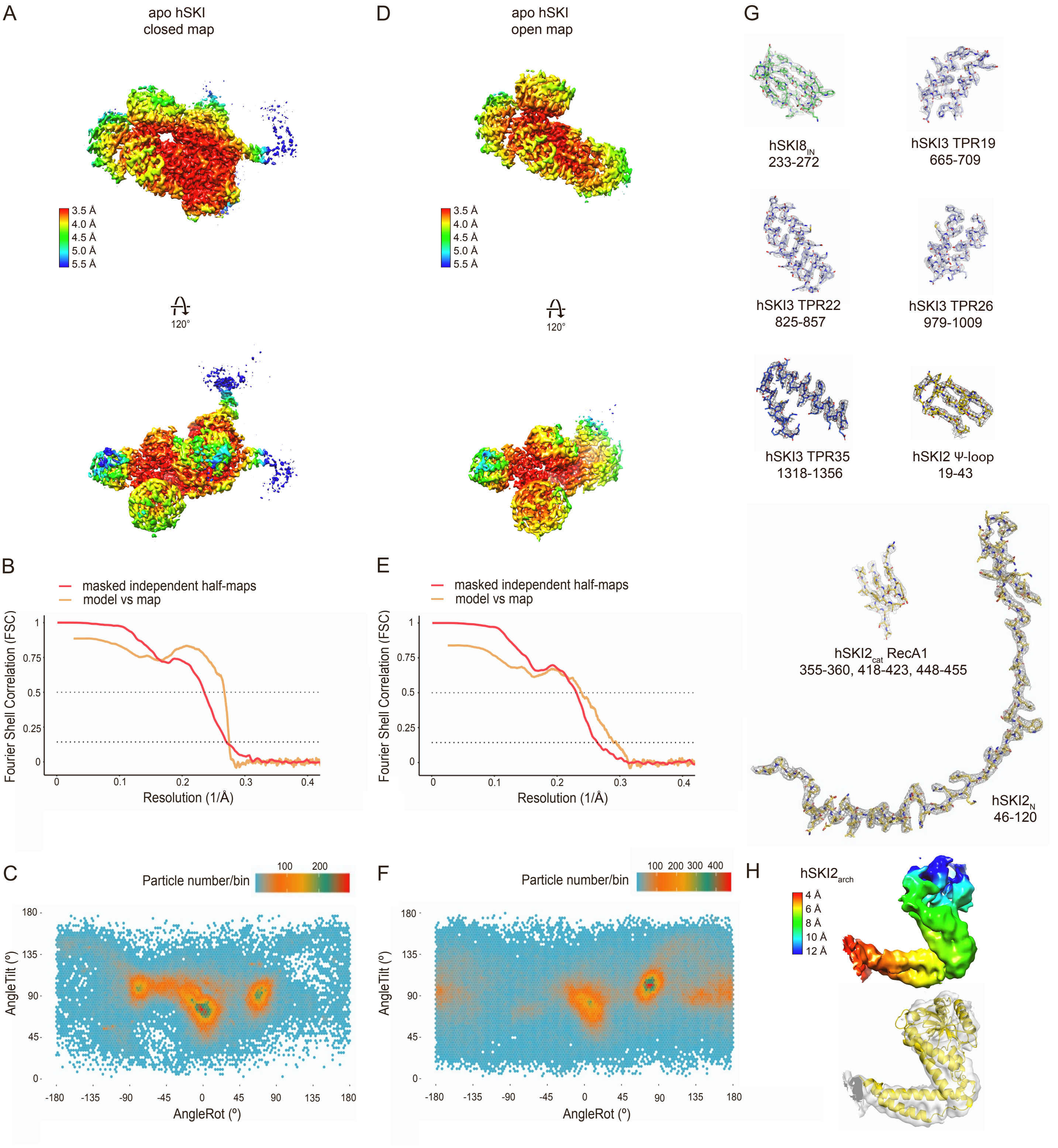
A
apo hSKI
closed map
3.5 Å
4.0 Å
4.5 Å
5.0 Å
5.5 Å
120°
D
apo hSKI
open map
3.5 Å
4.0 Å
4.5 Å
5.0 Å
5.5 Å
120°
G
hSKI8IN
233-272
hSKI3 TPR19
665-709
hSKI3 TPR22
825-857
hSKI3 TPR26
979-1009
hSKI3 TPR35
1318-1356
hSKI2 Ψ-loop
19-43
hSKI2cat RecA1
355-360, 418-423, 448-455
hSKI2N
46-120
B
masked independent half-maps
model vs map
Fourier Shell Correlation (FSC)
Resolution (1/Å)
E
masked independent half-maps
model vs map
Fourier Shell Correlation (FSC)
Resolution (1/Å)
C
Particle number/bin
100 200
AngleTilt (°)
AngleRot (°)
F
Particle number/bin
100 200 300 400
AngleTilt (°)
AngleRot (°)
H
hSKI2arch
4 Å
6 Å
8 Å
10 Å
12 Å

Figure S2

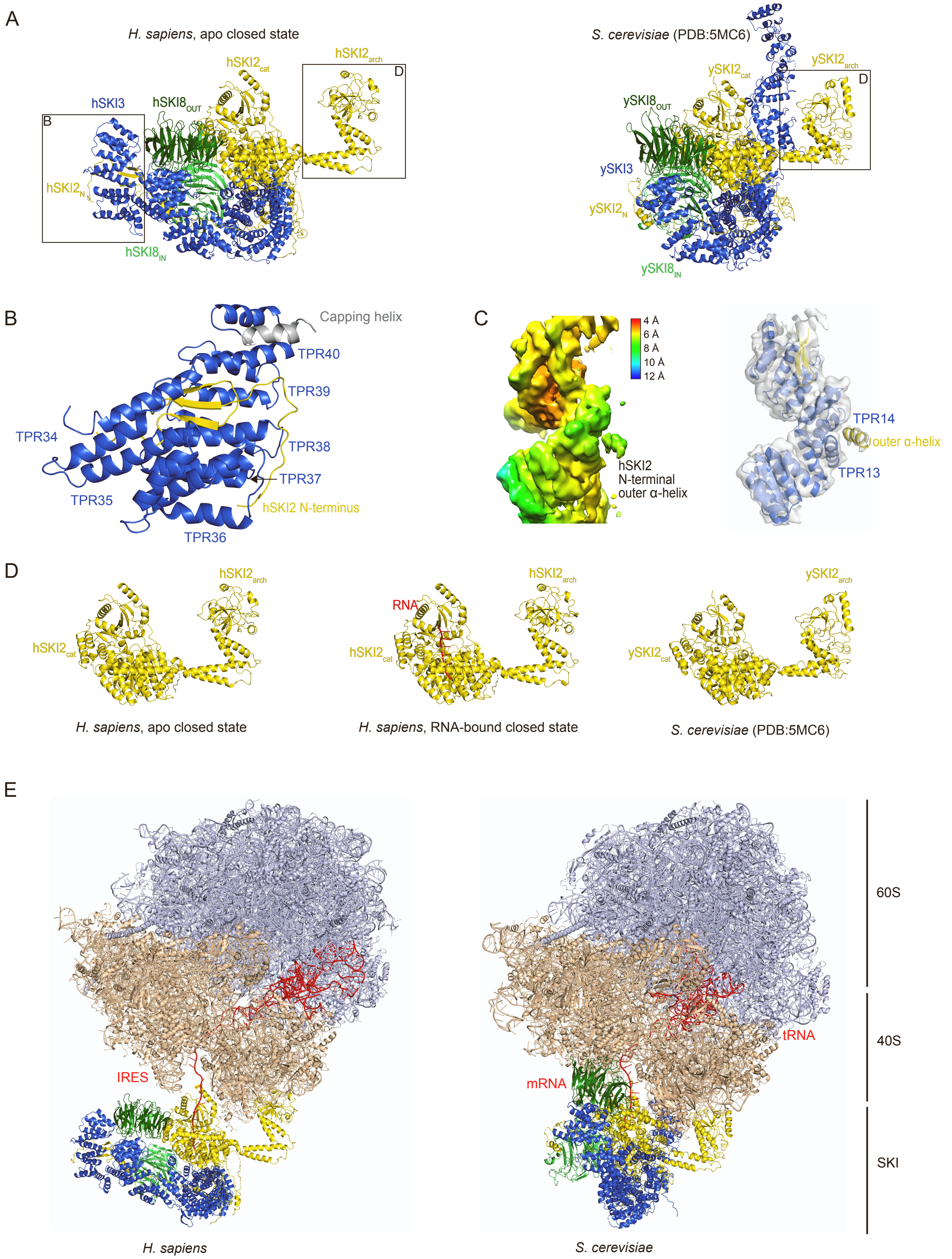
A
H. sapiens, apo closed state
hSKI2cat
hSKI2arch
hSKI3
hSKI8OUT
B
D
hSKI2N
hSKI8IN
S. cerevisiae (PDB:5MC6)
ySKI2cat
ySKI2arch
ySKI8OUT
ySKI3
ySKI2N
ySKI8IN
B
Capping helix
TPR40
TPR39
TPR34
TPR38
TPR37
TPR35
hSKI2 N-terminus
TPR36
C
4 Å
6 Å
8 Å
10 Å
12 Å
hSKI2
N-terminal
outer α-helix
TPR14
outer α-helix
TPR13
D
hSKI2arch
hSKI2cat
H. sapiens, apo closed state
RNA
H. sapiens, RNA-bound closed state
ySKI2arch
ySKI2cat
S. cerevisiae (PDB:5MC6)
E
IRES
mRNA
tRNA
60S
40S
SKI
H. sapiens
S. cerevisiae

Figure S3

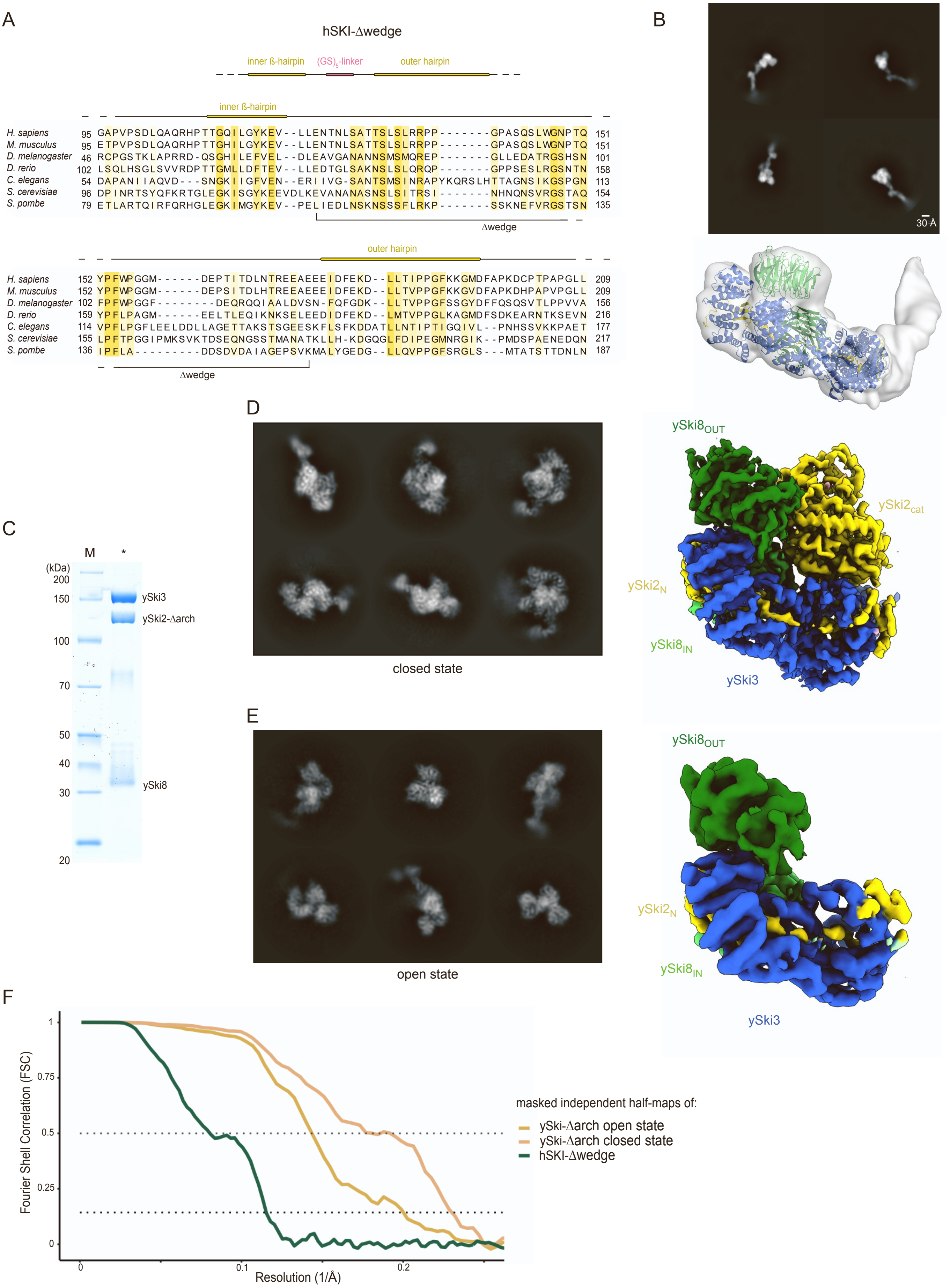
A
hSKI-Δwedge
inner ß-hairpin
(GS)5-linker
outer hairpin
inner ß-hairpin
H. sapiens 95 GAPVPSDLQAQRHPTTGQILGYKEV--LLENTNLSATTSLSLRRPP-------GPASQSLWGNPTQ 151
M. musculus 95 ETPVPSDLQAQRHPTTGHILGYKEV--LLENTNLSATTSLSLRRPP-------GPASQSLWGNPTQ 151
D. melanogaster 46 RCPGSTKLAPRRD-QSGHILEFVEL--DLEAVGANANNSMSMQREP-------GLLEDATRGSHSN 101
D. rerio 102 LSQLHSGLSVVRDPTTGMLLDFTEV--LLEDTGLSAKNSLSLQRQP-------GPPSESLRGSNTN 158
C. elegans 54 DAPANIIAQVD---SNGKIIGFVEN--ERIIVG-SANTSMSINRAPYKQRSLHTTAGNSIKGSPGN 113
S. cerevisiae 96 DPINRTSYQFKRTGLEGKISGYKEEVDLKEVANANASNSLSITRSI-------NHNQNSVRGSTAQ 154
S. pombe 79 ETLARTQIRFQRHGLEGKIMGYKEV--PELIEDLNSKNSSSFLRKP-------SSKNEFVRGSTSN 135
Δwedge
outer hairpin
H. sapiens 152 YPFWPGGM------DEPTITDLNTREEAEEEIDFEKD--LLTIPPGFKKGMDFAPKDCPTPAPGLL 209
M. musculus 152 YPFWPGGM------DEPSITDLHTREEAEEEIDFEKD--LLTVPPGFKKGVDFAPKDHPAPVPGLL 209
D. melanogaster 102 FPFWPGGF--------DEQRQQIAALDVSN-FQFGDK--LLTVPPGFSSGYDFFQSQSVTLPPVVA 156
D. rerio 159 YPFLPAGM------EELTLEQIKNKSELEEDIDFEKD--LMTVPPGLKAGMDFSDKEARNTKSEVN 216
C. elegans 114 VPFLPGFLEELDDLLAGETTAKSTSGEESKFLSFKDDATLLNTIPTIGQIVL--PNHSSVKKPAET 177
S. cerevisiae 155 LPFTPGGIPMKSVKTDSEQNGSSTMANATKLLH-KDGQGLFDIPEGMNRGIK--PMDSPAENEDQN 217
S. pombe 136 IPFLA---------DDSDVDAIAGEPSVKMALYGEDG--LLQVPPGFSRGLS---MTATSTTDNLN 187
Δwedge
B
30 Å
C
M
*
(kDa)
200
150
100
70
50
40
30
20
ySki3
ySki2-Δarch
ySki8
D
closed state
ySki8OUT
ySki2cat
ySki2N
ySki8IN
ySki3
E
open state
ySki8OUT
ySki2N
ySki8IN
ySki3
F
Fourier Shell Correlation (FSC)
1
0.75
0.5
0.25
0
0
0.1
0.2
Resolution (1/Å)
masked independent half-maps of:
ySki-Δarch open state
ySki-Δarch closed state
hSKI-Δwedge

Figure S4

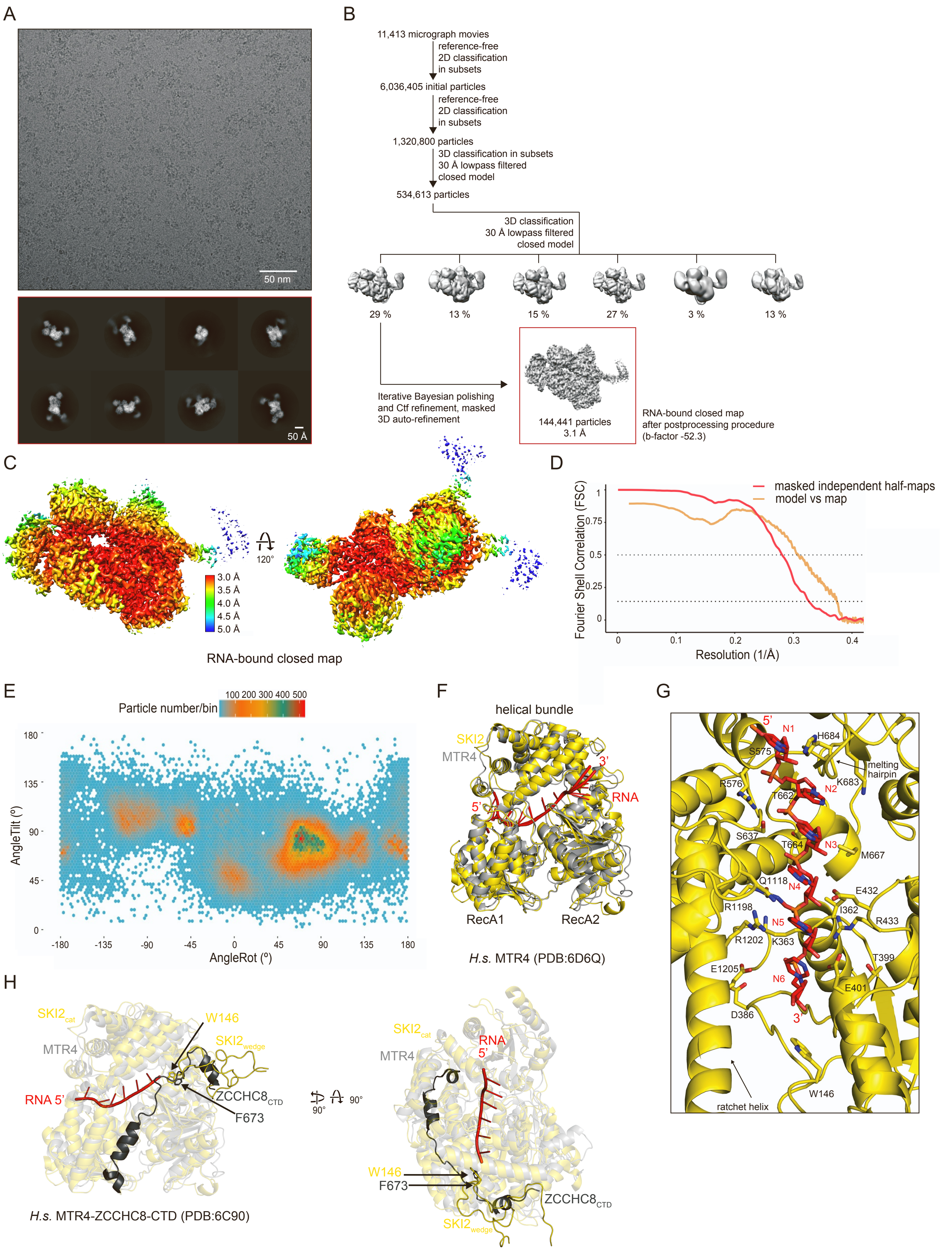
A
50 nm
50 Å
B
11,413 micrograph movies
reference-free 2D classification in subsets
6,036,405 initial particles
reference-free 2D classification in subsets
1,320,800 particles
3D classification in subsets
30 Å lowpass filtered closed model
534,613 particles
3D classification
30 Å lowpass filtered closed model
29 %
13 %
15 %
27 %
3 %
13 %
Iterative Bayesian polishing and Ctf refinement, masked 3D auto-refinement
144,441 particles
3.1 Å
RNA-bound closed map after postprocessing procedure (b-factor -52.3)
C
120°
3.0 Å
3.5 Å
4.0 Å
4.5 Å
5.0 Å
RNA-bound closed map
D
masked independent half-maps
model vs map
Fourier Shell Correlation (FSC)
Resolution (1/Å)
E
Particle number/bin
100 200 300 400 500
AngleTilt (°)
AngleRot (°)
F
helical bundle
SKI2
MTR4
3'
5'
RNA
RecA1
RecA2
H.s. MTR4 (PDB:6D6Q)
G
5'
N1
H684
S575
melting hairpin
K683
R576
N2
T662
S637
T664
N3
M667
Q1118
N4
E432
R1198
I362
R433
N5
R1202
K363
T399
E1205
N6
E401
D386
3'
ratchet helix
W146
H
SKI2cat
MTR4
W146
SKI2wedge
RNA 5'
ZCCHC8CTD
F673
90°
90°
RNA
5'
H.s. MTR4-ZCCHC8-CTD (PDB:6C90)

Figure S5

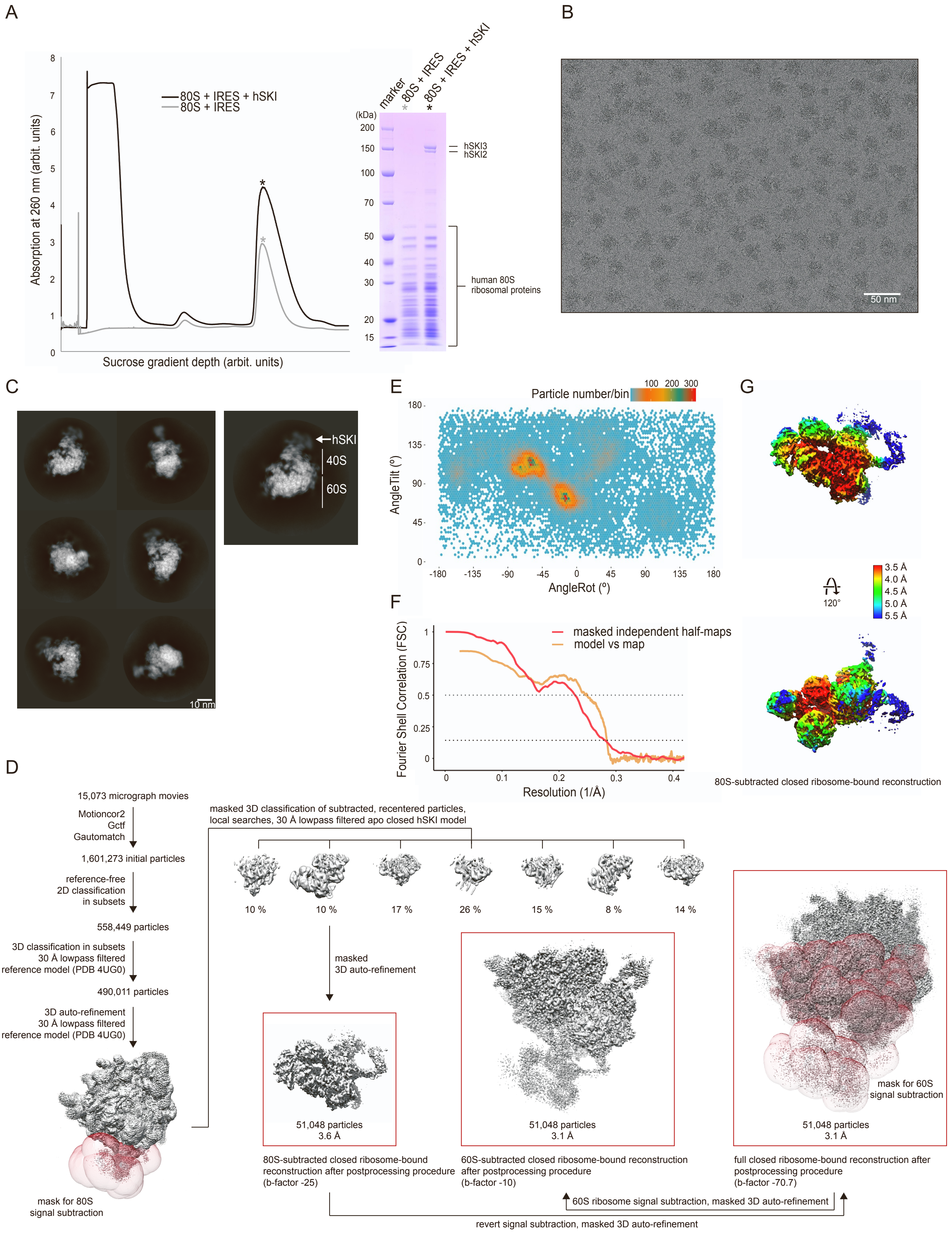
A
80S + IRES + hSKI
80S + IRES
Absorption at 260 nm (arbit. units)
Sucrose gradient depth (arbit. units)
marker
80S + IRES
80S + IRES + hSKI
(kDa)
hSKI3
hSKI2
human 80S
ribosomal proteins
B
50 nm
C
hSKI
40S
60S
10 nm
E
Particle number/bin
AngleTilt (°)
AngleRot (°)
F
Fourier Shell Correlation (FSC)
masked independent half-maps
model vs map
Resolution (1/Å)
G
120°
3.5 Å
4.0 Å
4.5 Å
5.0 Å
5.5 Å
80S-subtracted closed ribosome-bound reconstruction
D
15,073 micrograph movies
Motioncor2
Gctf
Gautomatch
1,601,273 initial particles
reference-free
2D classification
in subsets
558,449 particles
3D classification in subsets
30 Å lowpass filtered
reference model (PDB 4UG0)
490,011 particles
3D auto-refinement
30 Å lowpass filtered
reference model (PDB 4UG0)
mask for 80S
signal subtraction
masked 3D classification of subtracted, recentered particles,
local searches, 30 Å lowpass filtered apo closed hSKI model
10 %
10 %
17 %
26 %
15 %
8 %
14 %
masked
3D auto-refinement
51,048 particles
3.6 Å
80S-subtracted closed ribosome-bound
reconstruction after postprocessing procedure
(b-factor -25)
51,048 particles
3.1 Å
60S-subtracted closed ribosome-bound reconstruction
after postprocessing procedure
(b-factor -10)
mask for 60S
signal subtraction
51,048 particles
3.1 Å
full closed ribosome-bound reconstruction after
postprocessing procedure
(b-factor -70.7)
60S ribosome signal subtraction, masked 3D auto-refinement
revert signal subtraction, masked 3D auto-refinement

Figure S6

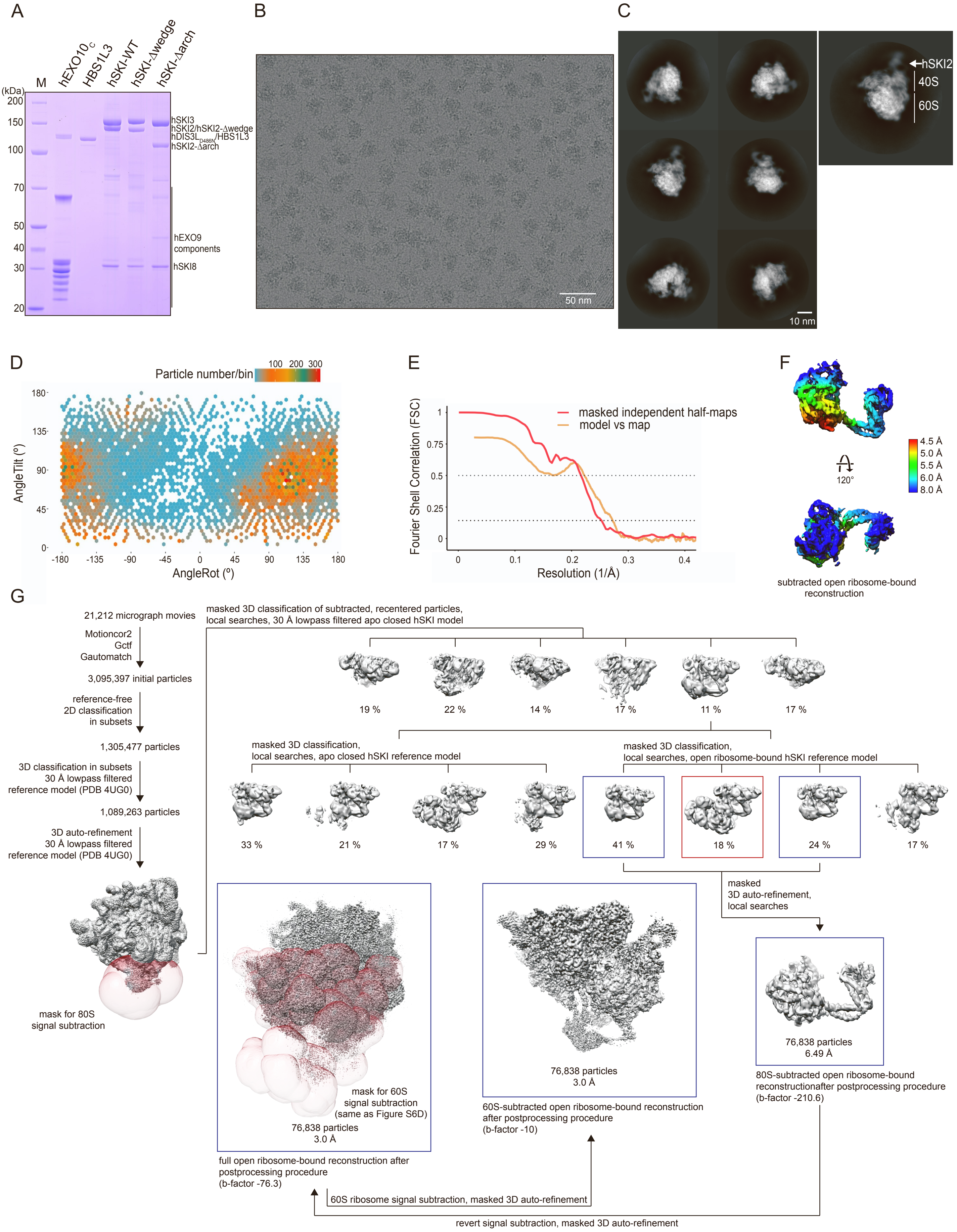

A
M
hEXO10C
HBS1L3
hSKI-WT
hSKI-Δwedge
hSKI-Δarch
(kDa)
200
150
100
70
50
40
30
20
hSKI3
hSKI2/hSKI2-Δwedge
hDIS3L D486N/HBS1L3
hSKI2-Δarch
hEXO9 components
hSKI8
B
50 nm
C
hSKI2
40S
60S
10 nm
D
Particle number/bin
100 200 300
AngleTilt (°)
AngleRot (°)
E
Fourier Shell Correlation (FSC)
masked independent half-maps
model vs map
Resolution (1/Å)
F
120°
4.5 Å
5.0 Å
5.5 Å
6.0 Å
8.0 Å
subtracted open ribosome-bound reconstruction
G
21,212 micrograph movies
Motioncor2
Gctf
Gautomatch
3,095,397 initial particles
reference-free 2D classification in subsets
1,305,477 particles
3D classification in subsets
30 Å lowpass filtered reference model (PDB 4UG0)
1,089,263 particles
3D auto-refinement
30 Å lowpass filtered reference model (PDB 4UG0)
mask for 80S signal subtraction
masked 3D classification of subtracted, recentered particles, local searches, 30 Å lowpass filtered apo closed hSKI model
19 %
22 %
14 %
17 %
11 %
17 %
masked 3D classification, local searches, apo closed hSKI reference model
33 %
21 %
17 %
29 %
masked 3D classification, local searches, open ribosome-bound hSKI reference model
41 %
18 %
24 %
17 %
masked 3D auto-refinement, local searches
mask for 60S signal subtraction (same as Figure S6D)
76,838 particles
3.0 Å
full open ribosome-bound reconstruction after postprocessing procedure (b-factor -76.3)
76,838 particles
3.0 Å
60S-subtracted open ribosome-bound reconstruction after postprocessing procedure (b-factor -10)
76,838 particles
6.49 Å
80S-subtracted open ribosome-bound reconstructionafter postprocessing procedure (b-factor -210.6)
60S ribosome signal subtraction, masked 3D auto-refinement
revert signal subtraction, masked 3D auto-refinement

Figure S7

Table S1. Cryo electron microscopy data collection summary, processing statistics and model quality indicators (related to Figures 1, 4, 5 and 6)

**Cryo electron microscopy data collection**					
Microscope	FEI Titan Krios GII				
Voltage (kV)	300				
Camera	Gatan K3-Summit				
Energy Filter	Gatan Quantum-LS (GIF)				
Pixel size (Å/pix)	1.094		0.851		
Preset target global defocus range (μm)	0.6 - 2.4				
**3D reconstruction**	**apo closed *H.s.* SKI**	**apo open *H.s.* SKI**	**RNA bound closed *H.s.* SKI**	**ribosome bound closed *H.s.* SKI**	**ribosome bound open *H.s.* SKI**
Number of movies	11,079		11,413	15,073	21,212
Exposure per sec (e^-/Å^2/s) /total exposure (e^-/Å^2)	11.86/47.44		22.77/68.31	11.16/55.8	11.27/67.62
Number of frames/movie	40		30	35	40
Initially selected particle candidates	4,400,241		6,036,405	1,601,273	3,095,397
Final number of particles	84,591	154,135	144,441	51,048	76,838
Resolution $_{\text{FSC independent halfmaps}}$ (Å)[a]	3.7	3.8	3.1	3.6	6.5
Local resolution range (Å)	3.3-15.8	3.4-13.1	2.8-12.6	3.1-15.6	4.6-10.7
Sharpening B-factor determined/applied (Å^2)	-88.1/-88.1	-145.3/-145.3	-52.3/-52.3	-73.6/-25	-210.6/-210.6
EMDB accession number	EMD-13923	EMD-13925	EMD-13927	EMD-13928	EMD-13929

Table S1 - continued.

**Refinement**	**apo closed *H.s.* SKI**	**apo open *H.s.* SKI**	**RNA bound closed *H.s.* SKI**	**ribosome bound closed *H.s.* SKI**	**ribosome bound open *H.s.* SKI**
PDB code	7QDR	7QDS	7QDY	7QDZ	7QE0
No atoms	22,827	13,798	22,933	22,598	7,619
Residues (protein)	2,991	1,771	2,991	2,924	949
Residues (RNA)	-	-	6	6	9
CC_{box}, CC_{mask}, CC_{volume}[b]	0.81, 0.86, 0.85	0.80, 0.82, 0.81	0.77, 0.80, 0.77	0.86, 0.81, 0.79	0.87, 0.74, 0.73
Resolution $_{\text{FSC map vs. model@0.143}}$ (Å)[b]	3.6	3.3	2.7	3.0	4.0
r.m.s. deviations					
Bond lengths (Å)	0.006	0.005	0.009	0.007	0.004
Bond angles (°)	0.819	0.852	1.101	0.969	0.998
Ramachandran favored (%)	94.25	94.32	94.39	95.05	96.19
Ramachandran allowed (%)	5.75	5.68	5.61	4.95	3.81
Ramachandran disallowed (%)	0.00	0.00	0.00	0.00	0.00
MolProbity score	1.99	2.04	1.96	2.09	2.07
Clash score	12.26	14.02	11.49	17.6	20.94

[a]according to the Fourier Shell Correlation (FSC) cut-off criterion of 0.143 defined in (Rosenthal and Henderson, 2003)
[b]according to the map-vs.-model Correlation Coefficient definitions in (Afonine et al., 2018)

Table S2. Kinetic parameters of hSKI ATP hydrolysis derived according to Michaelis-Menten (related to Figure 4A)

	**hSKI-WT**	**hSKI-Δarch**	**hSKI-Δwedge**	**hSKI-WT (no RNA)**	**hSKI-DEAD***	**hSKI-THES**
V_{max} (μM/s)	0.03427	0.04478	0.02990	0.002387	N/A	0.0009766
K_m (μM)	149.4	121.5	190.5	582.2	N/A	-2.574
k_{cat} (s^{-1})	0.6853	0.8957	0.5981	0.04775	N/A	0.01953
R^2	0.9817	0.9871	0.9822	0.2016	N/A	0.1477

*The hSKI-DEAD data could not be reconciled with the Michaelis-Menten equation. No kinetic parameters were derived (N/A, not applicable).
