# Supplementary material for: The human SKI complex regulates channeling of ribosome-bound RNA to the exosome via an intrinsic gatekeeping mechanism
Source: Mol Cell. 2022 Feb 17;82(4):756–769.e8. doi: 10.1016/j.molcel.2022.01.009 (PMC8860381; doi:10.1016/j.molcel.2022.01.009)
Supplement: Document S2. Article plus supplemental information [file mmc2.pdf]

Molecular Cell

Article

The human SKI complex regulates channeling of ribosome-bound RNA to the exosome via an intrinsic gatekeeping mechanism

Graphical abstract

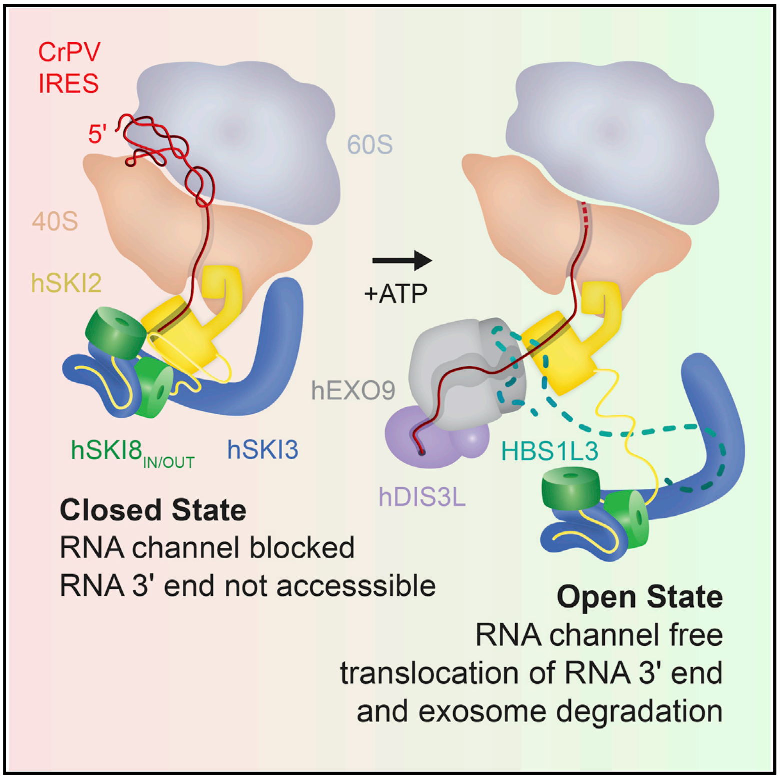

Highlights

- hSKI has closed and open states connected to different helicase conformations
- The intrinsic closed state traps the RNA 3′ end and blocks the RNA exit path
- ATP induces the open state of hSKI, allowing 80S ribosome-bound RNA extraction
- The hSKI open state primes hSKI2 for channeling RNA to the cytosolic exosome

Authors

Alexander Kögel, Achim Keidel, Fabien Bonneau, Ingmar B. Schäfer, Elena Conti

Correspondence

ischaefe@biochem.mpg.de (I.B.S.),
conti@biochem.mpg.de (E.C.)

In brief

Kögel et al. show that the human SKI complex adopts distinct conformational states to recognize and extract ribosome-bound RNA in a nucleotide-dependent manner. These functional states regulate access of the RNA 3′ end to the cytoplasmic human exosome for co-translational degradation.

Kögel et al., 2022, Molecular Cell *82*, 756–769
February 17, 2022 © 2022 The Author(s). Published by Elsevier Inc.
https://doi.org/10.1016/j.molcel.2022.01.009

CellPress

Molecular Cell

Article

The human SKI complex regulates channeling of ribosome-bound RNA to the exosome via an intrinsic gatekeeping mechanism

Alexander Kögel,[1] Achim Keidel,[1] Fabien Bonneau,[1] Ingmar B. Schäfer,[1,*] and Elena Conti[1,2,*]
[1]Department of Structural Cell Biology, Max Planck Institute of Biochemistry, Am Klopferspitz 18, 82152 Martinsried, Munich, Germany
[2]Lead Contact
*Correspondence: ischaefe@biochem.mpg.de (I.B.S.), conti@biochem.mpg.de (E.C.)
https://doi.org/10.1016/j.molcel.2022.01.009

SUMMARY

The superkiller (SKI) complex is the cytoplasmic co-factor and regulator of the RNA-degrading exosome. In human cells, the SKI complex functions mainly in co-translational surveillance-decay pathways, and its malfunction is linked to a severe congenital disorder, the trichohepatoenteric syndrome. To obtain insights into the molecular mechanisms regulating the human SKI (hSKI) complex, we structurally characterized several of its functional states in the context of 80S ribosomes and substrate RNA. In a prehydrolytic ATP form, the hSKI complex exhibits a closed conformation with an inherent gating system that effectively traps the 80S-bound RNA into the hSKI2 helicase subunit. When active, hSKI switches to an open conformation in which the gating is released and the RNA 3′ end exits the helicase. The emerging picture is that the gatekeeping mechanism and architectural remodeling of hSKI underpin a regulated RNA channeling system that is mechanistically conserved among the cytoplasmic and nuclear helicase-exosome complexes.

INTRODUCTION

The exosome complex is a conserved RNA-degradation machinery present in both the nucleus and the cytoplasm of all eukaryotes studied to date (Chlebowski et al., 2013; Januszyk and Lima, 2014; Mitchell et al., 1997). In the nucleus, the RNA exosome functions in the processing and decay of a large variety of noncoding transcripts as well as pre-mRNAs (Lingaraju et al., 2019b; Schmid and Jensen, 2019). In the cytoplasm, it primarily targets mRNAs (Schaeffer and van Hoof, 2011; Tuck et al., 2020). The processive ribonuclease module of the RNA-exosome complex is similar in both cellular compartments. Nine subunits form a catalytically inert cage that is traversed by an internal channel (Bonneau et al., 2009; Liu et al., 2006). This channel binds RNA and threads it to the 3′–5′ processive exoribonuclease in the complex: Rrp44 in yeast and the cytoplasmic and nuclear orthologs DIS3 and DIS3L in human (Dziembowski et al., 2007; Gerlach et al., 2018; Liu et al., 2006; Tomecki et al., 2010; Weick et al., 2018). The processive 10-subunit ribonuclease module (Exo-10) has an irreversible degrading action on the RNAs it has accessed. Exo-10 itself, however, lacks substrate specificity and requires different co-factors to target to different RNAs. The exosome co-factors exist in complexes that are compartment-specific and are centered around two RNA helicases, nuclear Mtr4 and cytoplasmic Ski2 (Lingaraju et al., 2019b; Olsen and Johnson, 2021; Weick and Lima, 2021). Together with their adaptors, the helicase co-factors of the exosome appear to control substrate access to the ribonuclease.

Ski2 and Mtr4 harbor similar biochemical properties. Both helicases hydrolyze ATP to power RNA unwinding in a processive manner and with 3′–5′ polarity, melting RNA secondary structures to create a progressively longer single-stranded 3′ end (Khemici and Linder, 2018). Both helicases also share a similar domain organization, with an N-terminal low-complexity region followed by a DExH-unwinding core and an additional domain with RNA-binding and protein-binding properties known as the "arch" (Halbach et al., 2012; Jackson et al., 2010; Weir et al., 2010). However, the two helicases differ in aspects that go beyond the mere presence (in Mtr4) or absence (in Ski2) of a nuclear localization signal. In particular, Mtr4/hMTR4 interacts with a variety of adaptor proteins, forming mutually exclusive complexes that target the different types of RNA substrates in the nucleus (Dobrev et al., 2021; Falk et al., 2014, 2017; Lingaraju et al., 2019a; Schuller et al., 2018; Thoms et al., 2015; Wang et al., 2019). In the cytoplasm, the Ski2/hSKI2 helicase instead mainly targets mRNAs and is part of a single assembly, the Ski complex (Anderson and Parker, 1998; Brown et al., 2000; Tuck et al., 2020).

The Ski proteins were discovered in yeast and named after the superkiller (SKI) phenotype—defined as increased susceptibility to a viral "killer" toxin in strains containing mutations in the *SKI* genes (Toh et al., 1978). The Ski2, Ski3, and Ski8 proteins assemble with a 1:1:2 stoichiometry to form a stable tetrameric

 © 2022 The Author(s). Published by Elsevier Inc.
This is an open access article under the CC BY license (http://creativecommons.org/licenses/by/4.0/).

assembly both *in vivo* and *in vitro* (Brown et al., 2000; Halbach et al., 2013; Synowsky and Heck, 2008). The crystal structure of a yeast Ski2-Ski3-Ski8 (Ski) complex has revealed a compact architecture, with the helicase core of Ski2 surrounded by the tetratricopeptide repeat (TPR) protein Ski3 and two WD40-repeat Ski8 subunits (Halbach et al., 2013). In biochemical assays, the RNA-dependent ATPase activity of the yeast Ski2 helicase is downregulated in the context of the Ski complex, but restored upon deletion of the Ski2 arch domain (Halbach et al., 2013). The Ski complex can also associate with translating 80S ribosomes, using the Ski2 arch domain as a major interaction site (Schmidt et al., 2016). The yeast 80S-Ski cryo-EM structure suggested that binding to ribosomes changes the conformation of the Ski2 arch domain, allowing 80S-bound mRNA to enter the helicase core of the Ski complex (Schmidt et al., 2016). The Ski complex also targets ribosome-free regions of mRNAs via the yeast-specific factor Ska1 (Zhang et al., 2019). Finally, the yeast Ski complex can bind Ski7, the adaptor that bridges the interaction to the Exo-10 exosome (Araki et al., 2001).

Orthologs of the yeast Ski2, Ski3, and Ski8 proteins can be identified in higher eukaryotes, such as the corresponding human proteins SKIV2L, TTC37, and WDR61, respectively. Deficiencies in SKIV2L and TTC37 cause trichohepatoenteric syndrome (THES), a congenital disease characterized by very early onset of chronic diarrhea and immune defects in children (Fabre et al., 2013). Several pathogenic mutations have been identified in THES patients, corresponding to either nonsense or missense mutations in SKIV2L or TTC37 (Fabre et al., 2011, 2012; Lee et al., 2016). Furthermore, human SKIV2L has also been linked to viral autoimmunity, as a mediator for the degradation of endogenous immuno-stimulatory RNAs that are produced by a cellular stress response (Eckard et al., 2014). SKIV2L activity has now been shown to act primarily in exosome-mediated degradation during co-translational mRNA surveillance pathways (Tuck et al., 2020) by extracting mRNA from stalled 80S ribosomes (Zinoviev et al., 2020). However, there is currently no structural information that would shed light on the physiological and pathological roles of the hSKI complex. In this work, we used biochemical and cryo-EM analysis to address the molecular mechanisms underlying the functions of the hSKI complex.

RESULTS AND DISCUSSION

The human SKI complex adopts closed and open states

For clarity, we will refer to the human orthologs of the yeast Ski complex subunits as hSKI2 (SKIV2L, ~137.8 kDa), hSKI3 (TTC37, ~175.5 kDa) and hSKI8 (WDR61, ~33.6 kDa) (Figure 1A). The hSKI2 subunit is a multidomain protein. In the text, we designate its individual domains as $hSKI2_N$ for the naturally unstructured N-terminal domain, $hSKI2_{cat}$ for the catalytic DExH core, and $hSKI2_{arch}$ for the arch domain (Figure 1A). In the case of hSKI3, analogous to the yeast ortholog, we refer to its N-terminal and C-terminal arch regions as $hSKI3_N$ and $hSKI3_C$ (Figure 1A; Halbach et al., 2013), respectively.

We co-expressed full-length hSKI2, hSKI3, and hSKI8 in insect cells and co-purified them as a homogeneous complex (Figure S1A). The purified hSKI complex was subjected to cryo-EM structural analysis (Figure S1B). Two-dimensional (2D) classifications of the cryo-EM images revealed the presence of two major subsets of particles (Figure S1C). Each subset was independently processed by three-dimensional (3D) classification followed by 3D refinement (Figure S1D). The subset with fewer particles (~40%) resulted in a reconstruction to a global resolution of ~3.7 Å and showed interpretable density for the majority of the complex (Figures 1B, 1C, and S2A–S2C). We will refer to this reconstruction as the "closed state" of hSKI. The second subset of particles (~60%) was refined to a similar resolution (~3.8 Å) and showed density for a smaller unit (Figures 1D, 1E, and S2D–S2F). We will refer to this reconstruction as the "open state" of hSKI.

The quality of the cryo-EM density map of the closed-state reconstruction enabled us to build most of the atomic model *de novo* (Figures 2A and S2G), with two exceptions. First, there was no ordered density for $hSKI3_N$ (predicted to contain TPRs 1–8); the N-terminal arm of hSKI3 was thus left unmodeled. Second, the density for the $hSKI2_{arch}$ domain displayed a local resolution of 4–12 Å in focused refinement (Figure S2H); therefore, this domain was built by docking a model generated via a structural prediction based on the yeast ortholog (Figure 2A; Halbach et al., 2012; Tunyasuvunakool et al., 2021). In the case of the open-state reconstruction there was no ordered density that would account for the $hSKI2_{cat}$ and $hSKI2_{arch}$ domains, suggesting that they detached from the rest of the complex (Figures 1D and 1E). Based on the structural analysis, we define $hSKI2_{cat}$ and $hSKI2_{arch}$ as the helicase module of hSKI, whereas $hSKI3_C$, $hSKI2_N$, and the two hSKI8 subunits are designated as the gatekeeping module (Figures 1C and 1E).

The gatekeeping module of hSKI forms the epicenter of the complex

We will start by describing the closed-state conformation of hSKI, which is generally similar to that observed in the structure of the *Saccharomyces cerevisiae* ortholog (Halbach et al., 2013; Figures 2A and S3). hSKI is scaffolded around its largest subunit, the TPR-containing protein hSKI3. TPRs are structural repeats consisting of two α-helices that arrange in tandem to form right-handed solenoids, with superhelical turns of approximately 8 TPRs each (Perez-Riba and Itzhaki, 2019). The TPRs 9–40 of the $hSKI3_C$ arm form a crescent-shaped solenoid with four superhelical turns (TPRs 9–16, 17–24, 25–32, 33–40) (Figures 1A and 2A). Most of the fourth superhelical turn is an extension of hSKI3 as compared with the yeast ortholog (Halbach et al., 2013; Figures S3A and S3B). This superhelical turn of hSKI3 is also a hotspot for disease-associated mutations in THES patients (L1485R, R1503C, and L1505S at TPR39—hereby defined as THES-1 hotspot, Figure 2B), suggesting that this region has physiological relevance. Since THES-associated mutations (Fabre et al., 2013) map to many different areas of the complex, we will point to them in the text when describing the corresponding structural features that are affected.

The $hSKI3_C$ arm wraps around the $hSKI2_N$ domain (Figure 2B, left panel). The $hSKI2_N$ domain is an extended region that can be subdivided into individual segments (Figure 2C). The first ("inner") segment (hSKI2 residues 1–121) binds inside the superhelical axis of $hSKI3_C$ with sparse secondary structure elements, spanning almost the entire length of the solenoid and forming

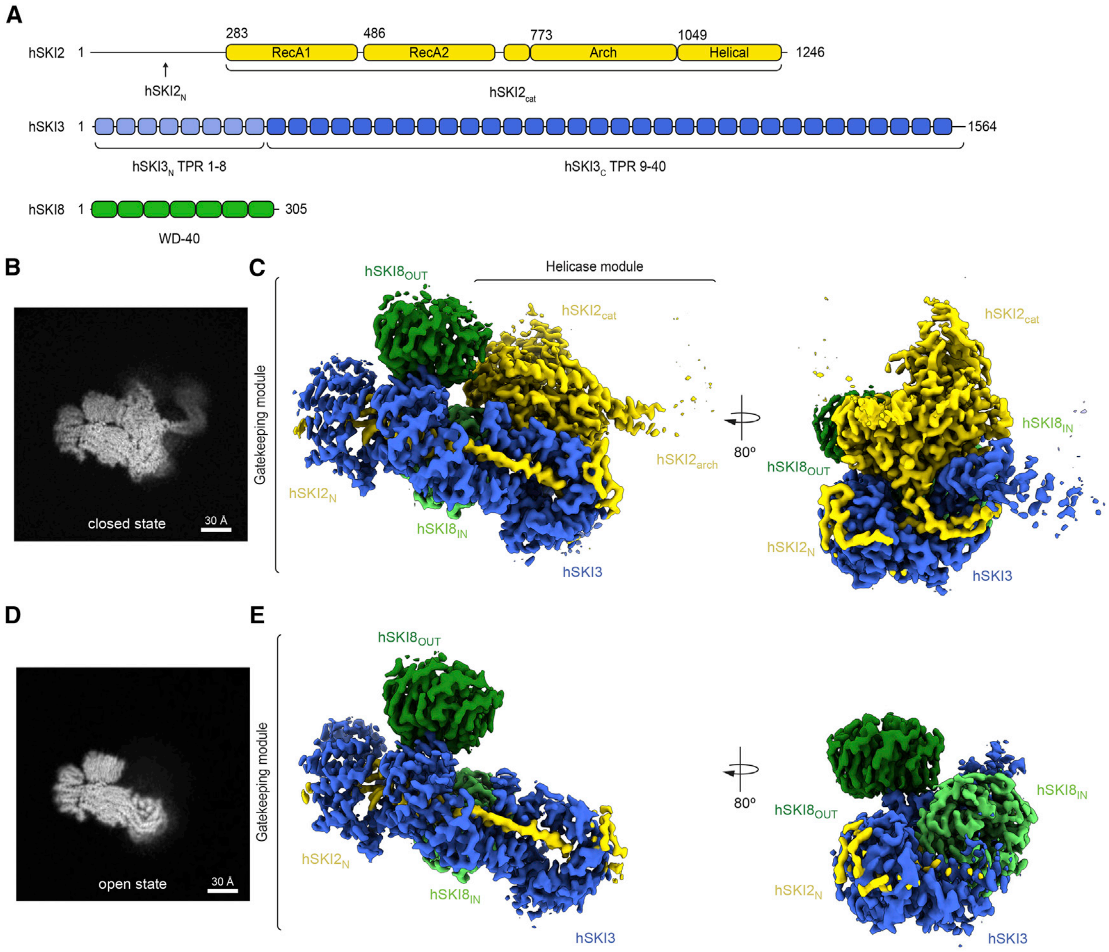

Figure 1. Structural organization and conformational states of hSKI

(A) Domain organization of the human SKI subunits hSKI2 (yellow), hSKI3 (blue), and hSKI8 (green). Predicted folds segments are indicated by rectangles and extended segments by lines. The N-terminal arm of hSKI3 (hSKI3$_N$; TPR 1–8) (light blue) is flexible in all current structural data.

(B and C) Single-particle cryo-EM reconstruction of the closed state of apo hSKI at a global resolution of 3.7 Å. 2D projection of the final reconstruction, showing the density for the hSKI2$_{arch}$ (B). Three-dimensional rendering of the reconstruction with the hSKI subunits in two orientations and colored as in (A). (C) The gatekeeping module (hSKI2$_N$-hSKI3-hSKI8$_{IN}$-hSKI8$_{OUT}$) and the helicase module (hSKI2$_{cat}$-hSKI2$_{arch}$) discussed in the text are indicated.

(D and E) Single-particle cryo-EM reconstruction of the open state of apo hSKI at a global resolution of 3.8 Å. The 2D projection (D) and the 3D reconstruction (E) are in a similar orientation as the closed state in (B) and (C). Only the gatekeeping module is visible in both 2D projection and 3D rendering.

See also Figures S1 and S2; Table S1.

an integral part of its hydrophobic core (Figure 2B, left panel, box 1). This segment ends with a highly conserved "inner ß-hairpin" that is embedded in the second superhelical turn of hSKI3$_C$ (at TPRs 17–19) (Figure 2B; box 2) and connects to an intricate loop structure that we will refer to as the "wedge" segment (hSKI2 residues 122–165) (Figure 2B; box 3). The hSKI2$_N$ wedge segment protrudes from the concave surface of hSKI3$_C$ at the second superhelical turn and, despite lacking secondary structure elements, is well structured by intra- and intermolecular interactions. hSKI2$_N$ continues by binding with an "outer hairpin" (hSKI2$_N$ residues 172–201) along the external convex surface of the solenoid, at the second superhelical turn of hSKI3$_C$ (Figure 2B; box 4). From here, the density of hSKI2$_N$ weakens as it reaches the first superhelical turn of hSKI3$_C$ with an "outer α-helix" (Figures 2C and S3C) and then fades at the linker segment that connects to the RecA1 domain of the well-ordered helicase module.

The hSKI3$_C$ arm also binds the hSKI8 subunits. hSKI8 is a seven-bladed ß-propeller with the wheel-like shape characteristic of WD40-repeat proteins (Stirnimann et al., 2010). The outer hSKI8 subunit (hSKI8$_{OUT}$) adopts an outward position at the third superhelical turn of hSKI3$_C$ (Figure 2A). The internal hSKI8 subunit (hSKI8$_{IN}$) is positioned at the inner concave surface of hSKI3$_C$, interacting with the third superhelical turn (Figure 2A, left panel). THES-associated mutations in hSKI3 map to the hSKI8$_{IN}$-binding site (P1270A and D1283N at TPR 33 and 34). These substitutions (THES-2 hotspot; Figure 2B) are expected to weaken the intermolecular interactions and/or stability of the gatekeeping module. Another cluster of THES-associated mutations in hSKI3 map to the region that wraps around the inner

CellPress
OPEN ACCESS

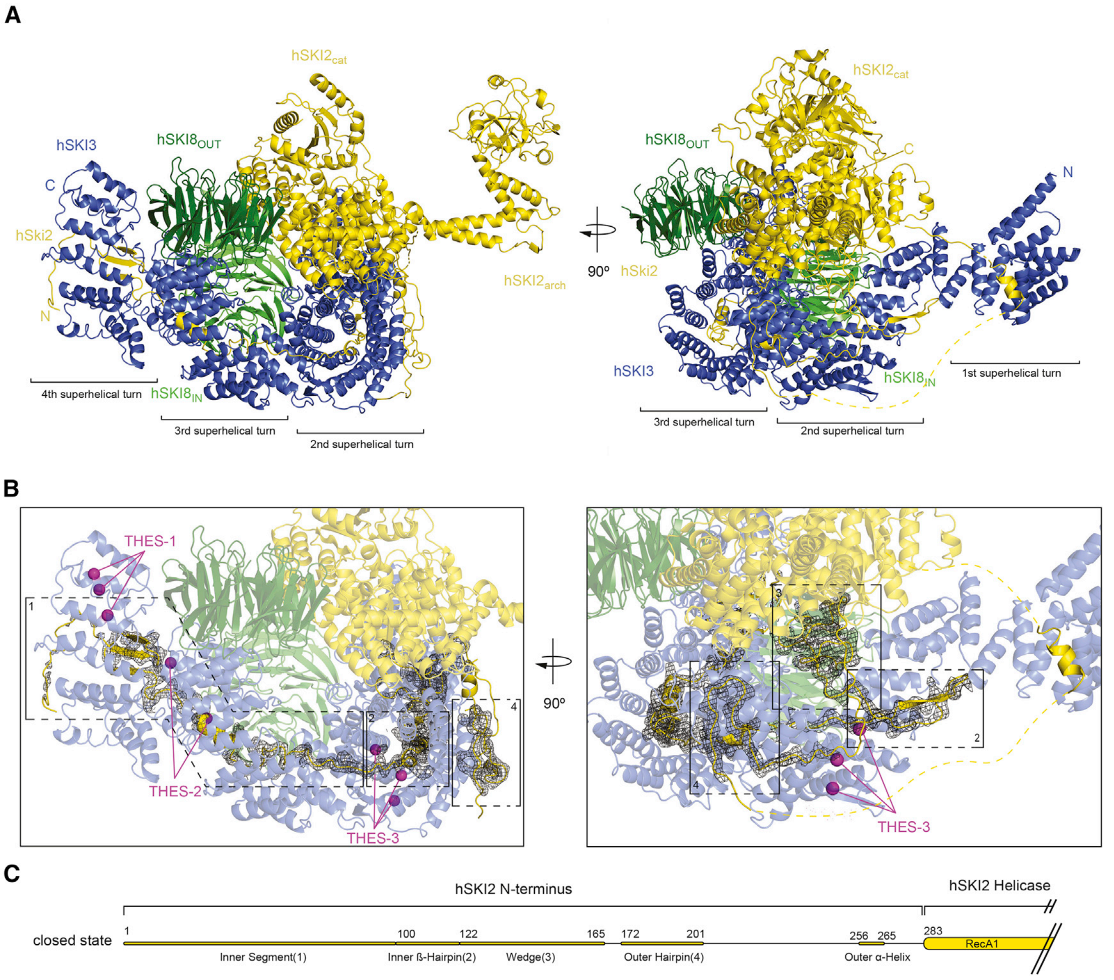

Figure 2. Closed-state conformation of hSKI

(A) Cartoon representations of hSKI in closed state, related by a 90° rotation around a vertical axis (left panel oriented as in Figure 1, same color scheme).
(B) Zoom-in of the gatekeeping module with the difference density for $SKI2_N$ displayed as black mesh. The two representations are oriented as those in (A). Numbering refers to the regions indicated in (B). Highlighted as purple spheres are the positions of THES disease mutants in the core of the gatekeeping module (clustered in the THES-1, THES-2 and THES-3 groups). The numerals 1–4 refer to the regions shown in (C).
(C) Schematic domain organization of $hSKI2_N$ highlighting distinct regions discussed in the text.
See also Figures S1, S2, and S3; Table S1.

segment and are expected to interfere with the proper folding of the superhelix (hSKI3 G673D, G721R, L761P at TPRs 19 and 20, THES-3 hotspot) (Figure 2B).

The helicase module can detach from the gatekeeping module

In the closed-state conformation of SKI, the basal surface of the $hSKI2_{cat}$ domain binds the hSKI3 solenoid at the first and second superhelical turns and hereby interacts with the adjacent $hSKI8_{OUT}$ and $hSKI8_{IN}$ subunits and with the wedge segment of $hSKI2_N$ (Figure 2). The $hSKI2_{arch}$ insertion instead protrudes from the top surface of the $hSKI2_{cat}$ domain, extending into solvent with a curved structure (Figure 2A). In the open-state conformation, not only is there no well-ordered density for the hSKI2 helicase module, but neither is there density for the wedge segment of $hSKI2_N$ or for the first superhelical turn of $hSKI3_C$ including the $hSKI2_N$ outer α-helix (Figures 3A and 3B). In contrast, the internal segment, both the inner ß-hairpin and the outer hairpin of $hSKI2_N$ are bound in the same manner to hSKI3. Thus, in this open-state conformation of hSKI, the hSKI2 helicase module appears to be flexible while it remains linked to the gatekeeping module via the $hSKI2_N$ domain.

The structural analysis suggests that the inner and outer hairpins of $hSKI2_N$ anchor the wedge segment as it undergoes conformational changes connected to the recruitment or detachment of the helicase core from the gatekeeping module. To test the impact of the wedge on the movement of the helicase module, we replaced this segment with a $(Gly\text{-}Ser)_5$ linker and purified the corresponding hSKI-Δwedge mutant complex for cryo-EM analysis (Figures S4A and S4B). The entire dataset of hSKI-Δwedge showed the presence of a complex in the open state (Figure S4B). Thus, the wedge segment of $hSKI2_N$ appears

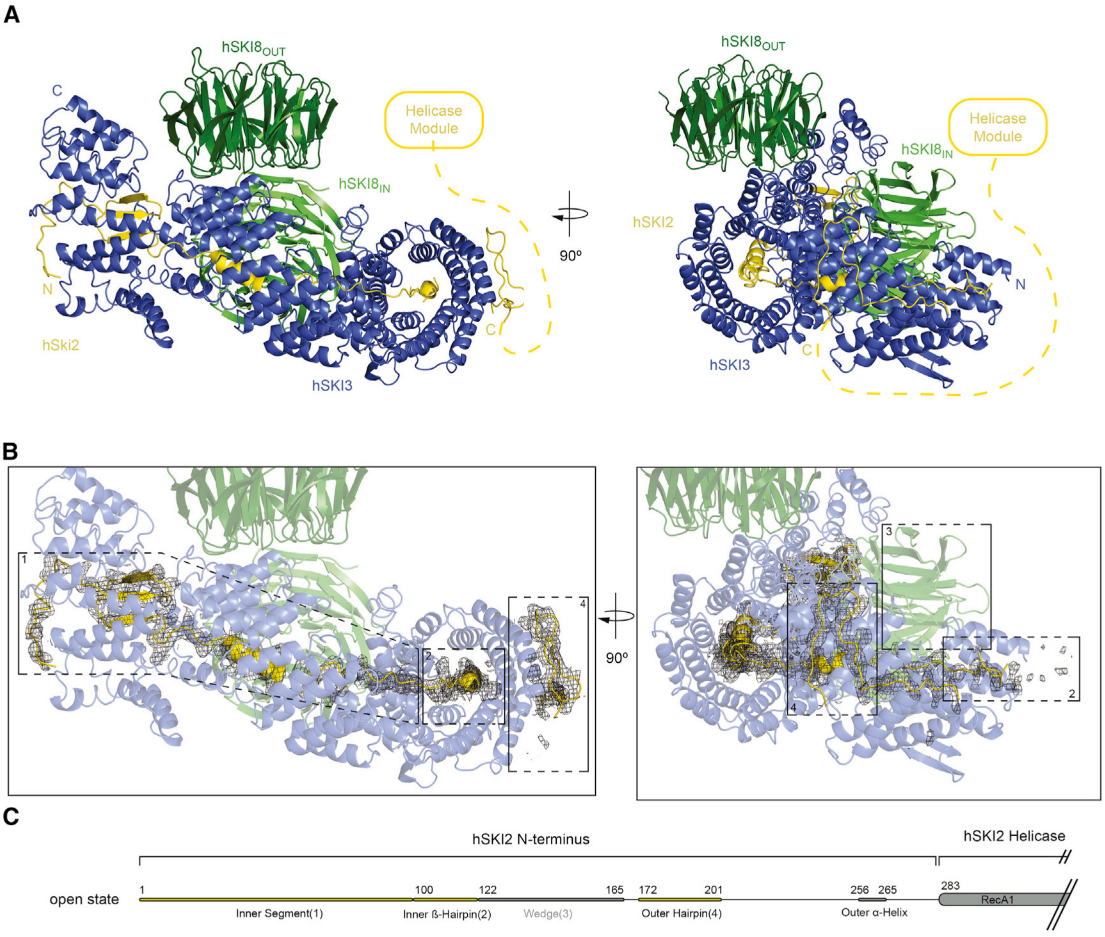

Figure 3. Open-state conformation of hSKI

(A) Cartoon representations of hSKI in open state, oriented as in Figure 2. The connection to the disengaged helicase module is indicated by a dotted yellow line.
(B) Zoom-in of the gatekeeping module with the difference density for $SKI2_N$ displayed as black mesh. The two representations are oriented as in (A). Note that the density for the $hSKI2_N$ wedge (3) is absent and the $hSKI2_N$ inner ß-hairpin (2) is less well-ordered compared with the closed-state reconstruction in Figure 2B.
(C) Schematic domain organization of $hSKI2_N$ highlighting the distinct regions discussed in the text.
See also Figures S1, S2, and S4; Table S1.

to stabilize the closed state. Notably, the structure of the apo *S. cerevisiae* Ski-Δarch complex also showed a similar closed-state architecture with an analogous wedge segment (RG motif, R149-G150) positioned at the bottom of the empty helicase channel (Halbach et al., 2013). However, it is likely that the crystallization procedure selected the most compact conformation for lattice formation, since cryo-EM analysis of the same complex indicates that it is present both in closed and open states (Figures S4C–S4E). Thus, we posit that the presence of the open and closed conformational states is an evolutionarily conserved feature of the Ski complex.

RNA is enclosed in the helicase core in closed-state hSKI

We proceeded to characterize how hSKI binds RNA. In biochemical spectrophotometric enzyme-coupled assays, recombinant hSKI showed RNA-dependent ATPase activity (hSKI-WT) (Figure 4A; Table S2). As control, the ATPase activity of hSKI was abolished in the case of a mutant complex with a glutamic acid to glutamine substitution in the helicase catalytic site (hSKI-DEAD containing the E424Q substitution in hSKI2) (Figure 4A; Table S2). We determined the kinetic parameters for the wild-type complex under steady-state conditions for varying ATP concentrations. Half-maximum velocity was reached at K_m = 149.4 μM ATP with a k_{cat} = 0.685 s^{-1}, which is well under physiological ATP concentrations, similar to *S. cerevisiae* Ski (Halbach et al., 2013).

For the structural analysis, we incubated wild-type recombinant hSKI with a 25-uracil (25U) RNA substrate and the nonhydrolyzable nucleotide analog ADP-BeF (which mimics a prehydrolytic ATP state). Cryo-EM data showed that essentially all particles were in the closed-state conformation, resulting in a 3D reconstruction of RNA-bound hSKI at a global resolution of 3.1 Å (Figures 4B and S5). The cryo-EM reconstruction revealed well-defined density for six ribonucleotides (N1–N6) bound in $hSKI2_{cat}$ in a single-stranded conformation (Figure 4B). $hSKI2_{cat}$

CellPress
OPEN ACCESS

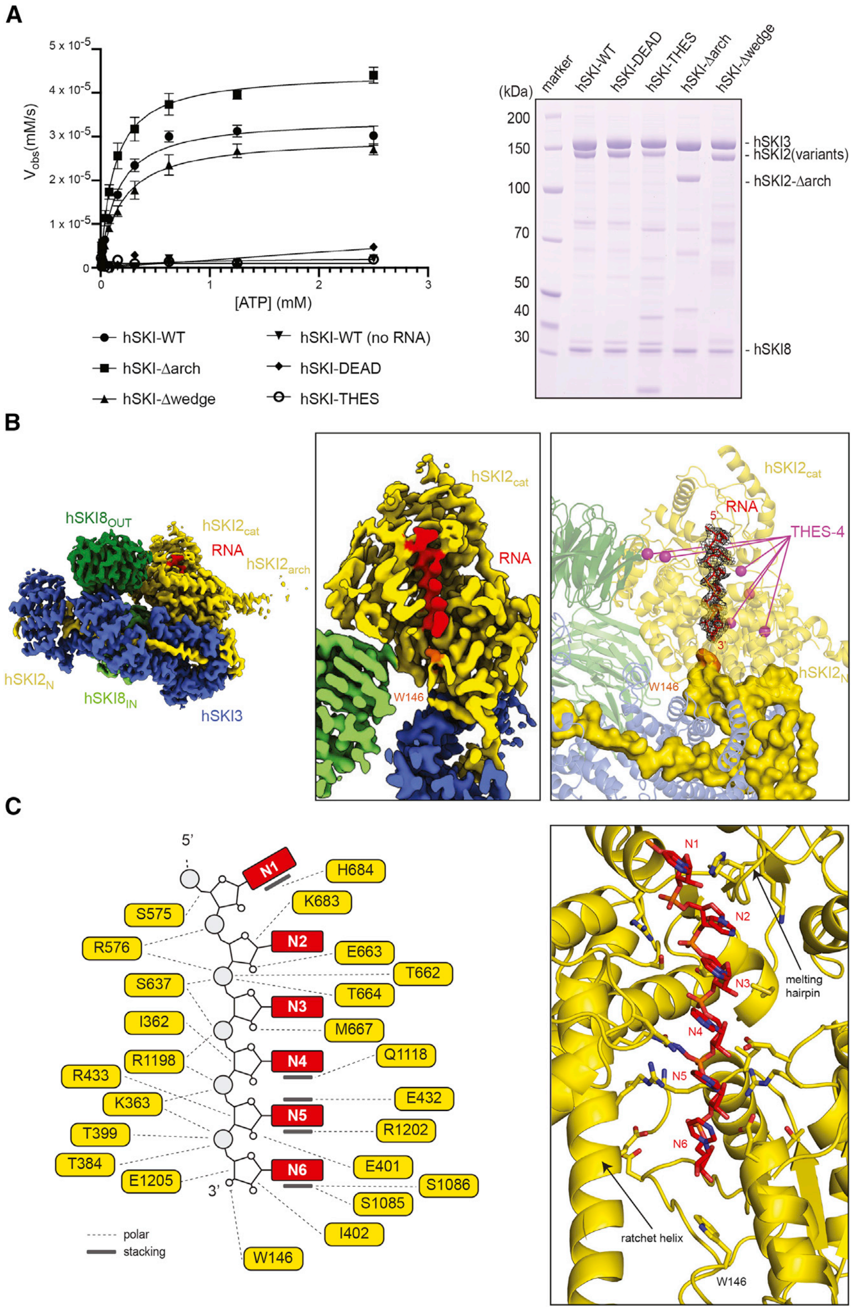

Figure 4. RNA-binding features of hSKI

(A) RNA-dependent ATPase activity of hSKI wild-type and mutants. The mutations Δarch, Δwedge, E424Q (DEAD), and V341G (THES) are in the hSKI2 subunit of the complex. (Left) Enzyme-coupled spectrophotometric assay. Initial ATPase rates are plotted against ATP concentration. Protein and poly(U) RNA concentrations were 50 nM and 10 μg/mL, respectively. Data were fitted according to Michaelis-Menten kinetics (see Table S2 for derived kinetic parameters). Error bar: ±1 standard deviation from three independent experiments. (Right) Coomassie-stained 4%–12% SDS-PAGE of the hSKI samples used in the assay. hSKI2 (variants) refers to all mutants except hSKI2Δarch.

(B) RNA path in the structure of RNA-bound hSKI. The central panel shows a slap view of the 3D reconstruction through the central plane of the RNA channel in $hSKI2_{cat}$. On the right is a zoom-in highlighting the path of the RNA (cartoon representation in red, difference density in black mesh) in hSKI2. The $hSKI2_N$ wedge is indicated in space-fill representation. The position of W146 of the $hSKI2_N$ wedge is indicated in orange. Highlighted as purple spheres are the positions of THES disease mutants in this area of the complex (clustered in the THES-4 group). Note that N1–N6 correspond to uracil nucleotides in the homopolymeric 25U RNA we used in the structural analysis.

(C) Detailed interactions between hSKI2 and the six ordered nucleotides at the RNA 3′ end. (Right) Zoom-in of the structure at the RNA-binding residues. (Left) Corresponding schematic of the interactions (polar and stacking contacts are indicated).

See also Figure S5; Table S1 and S2.

has the characteristic domain organization of DExH helicases: a pair of RecA domains (RecA1 and RecA2) contain the ATPase catalytic site and are juxtaposed to a helical domain, resulting in an overall globular shape with a central channel (Figure S5F) (Ozgur et al., 2015). The 5′ nucleotide (N1) binds at the top surface of $hSKI2_{cat}$, where it engages in base-stacking interactions with the so-called melting hairpin of RecA2 (Figures 4C and S5G). This motif is a conserved structural feature poised to melt RNA duplexes as they enter the DExH core of Ski2-related helicases (Büttner et al., 2007). The ribonucleotide chain then binds along the RNA-binding surface of RecA2 and continues into the helicase channel, threading between the RecA1 domain and the helical domain (at the so-called ratchet helix) (Büttner et al., 2007) (Figure 4C). Several disease-associated mutations in THES patients target the DExH core of hSKI2 in proximity to the ATP-binding site (A332P, V341G, E438K, and R483C substitutions) or adjacent to the RNA-binding surfaces (ΔG1187-Q1193 and ΔS1189-L1195 deletions) and are thus expected to affect the RNA-dependent ATPase properties of the complex (THES-4 hotspot) (Figure 4B, right panel). To test this prediction, we expressed and purified a recombinant hSKI complex mutant with the V341G substitution (hSKI-THES) and indeed found that it is inactive in ATPase assays *in vitro* (Figure 4A; Table S2).

The RNA-binding interactions of the DExH core are evolutionarily conserved not only in hSKI2 orthologs but also in the nuclear helicase Mtr4 (Figure S5F) (Gerlach et al., 2018; Schuller et al., 2018; Weick et al., 2018; Weir et al., 2010). However, the cryo-EM density of hSKI revealed an additional and unexpected feature: the most 3′ end ribonucleotide interacts with the wedge segment of the $hSKI2_N$ domain (Figures 4B and 4C). In particular,

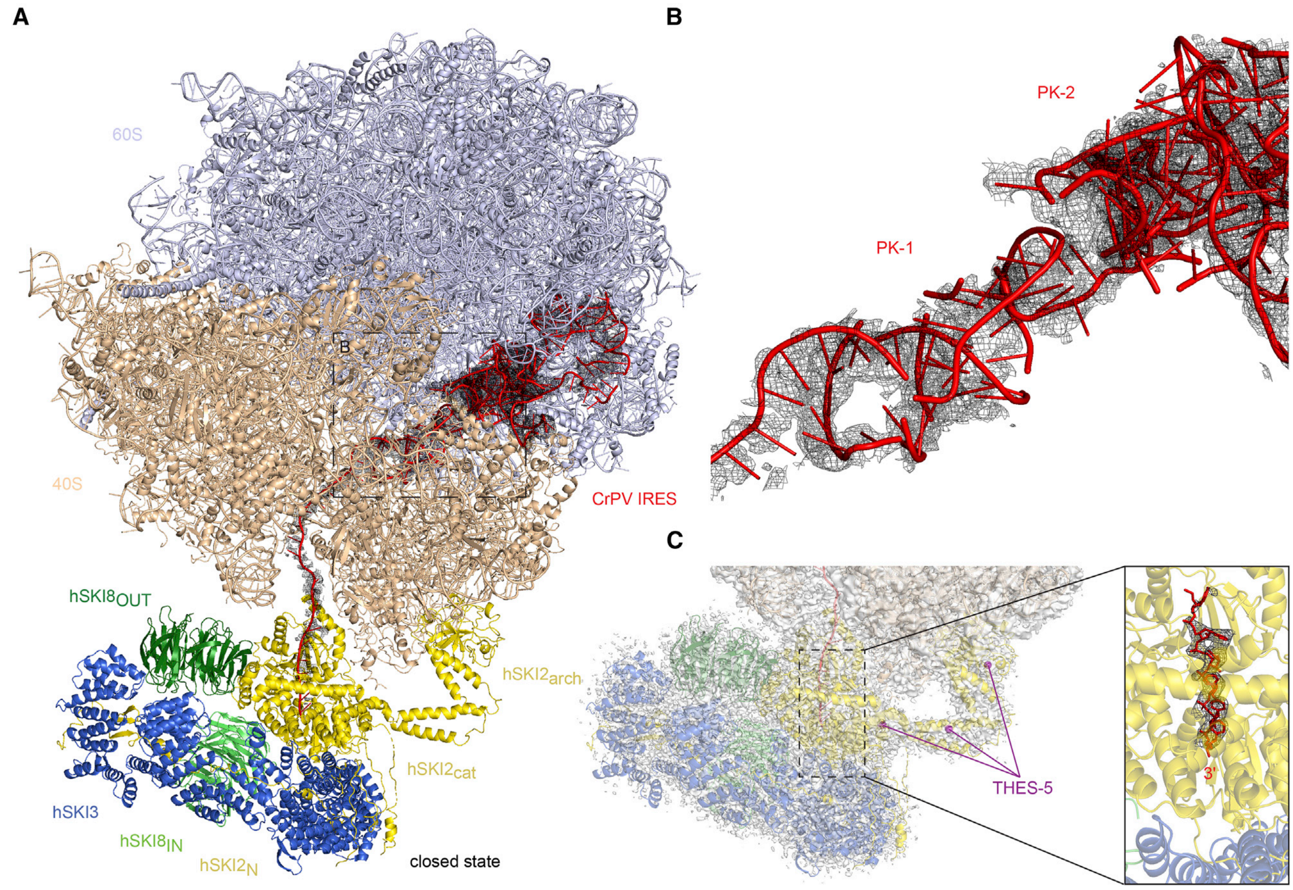

Figure 5. RNA blocked in the hSKI2 helicase in a close-state hSKI-80S complex

(A) Cartoon representation of hSKI bound in a closed state to a human 80S ribosome reconstituted with a CrPV IRES-29 nt RNA. The large and small ribosomal subunits are in light blue and light orange, respectively, and CrPV IRES-29 RNA is in red with corresponding density indicated by black mesh.

(B) Zoom-in view of the 80S-bound RNA at CrPV IRES pseudoknots 1 and 2 (PK-1 and PK-2) with corresponding density.

(C) Zoom-in view of the 80S-bound RNA at the 3′ end. The enlarged photo on the right panel displays the 6 nt that are traceable in hSKI2 in closed-state hSKI. See also Figure S6; Table S1.

a Trp-Gly motif (W146-G147) in the $hSKI2_N$ wedge segment contacts the 3′ hydroxyl group of the N6 ribonucleotide enclosed in the helicase channel. The interactions suggest the presence of a crosstalk between the $hSKI2_N$ wedge loop in the gatekeeping module and the 3′ end of an RNA substrate while in the $hSKI2_{cat}$ core, rationalizing the presence of a single class of particles/ states during 3D refinement of the RNA-bound data set as compared with the different subsets of closed and open states in the apo hSKI reconstructions.

80S-bound hSKI is in a closed state in the presence of a prehydrolytic ATP mimic

The observation that the hSKI gatekeeping module obstructs the end of the helicase channel in the closed state raised the hypothesis that in the open state the obstruction in hSKI2 is removed and the exit for the RNA substrate is opened. However, this prediction could not be verified in the context of hSKI complexes in isolation because it was not possible to visualize the detached hSKI2 in open-state hSKI (as the larger gatekeeping module dominates the particle alignment). We proceeded to visualize hSKI bound to a human ribosome (Figure 5).

To reconstitute an appropriate RNA-containing 80S sample, we took advantage of the internal ribosome entry site (IRES) from the cricket-paralysis virus (CrPV) and added a 29-nt sequence at the 3′ end (IRES29 RNA). The CrPV-IRES folds into a stable structure capable of binding directly to the intersubunit space on the ribosomal 40S subunit, which can then join a 60S subunit to form an 80S ribosome (Hellen, 2009). The additional sequence at the 3′ end was included to create a suitable binding platform for hSKI based on previous studies (Schmidt et al., 2016; Zinoviev et al., 2020). In human cells, the SKI complex has been shown to extract 80S-bound mRNAs that contain a 3′ terminal region of at least 19 nt from the P site (Zinoviev et al., 2020).

We incubated the reconstituted 80S-IRES29 with an excess of wild-type hSKI and the nucleotide analog ADP-BeF and purified the assembly by gradient centrifugation for cryo-EM data collection. Data processing yielded a reconstruction with an overall resolution of ~3.1 Å (Figure S6). The density showed significant

CellPress
OPEN ACCESS

flexibility for hSKI, thus requiring an adjustment to the center of mass by subtracting the ribosome signal from the data. The resulting map of 80S-bound hSKI reached a global resolution of 3.6 Å in the core of the helicase complex (Figure S6) and was interpreted by rigid-body fitting the model from the substrate-bound hSKI reconstruction, with no major differences with regard to the position of the four hSKI subunits. The structure of 80S-bound hSKI in the presence of a prehydrolytic ATP mimic thus shows that the hSKI2 helicase module is recruited to the hSKI gatekeeping module in the characteristic closed-state conformation (Figure 5A).

hSKI is flexibly tethered to 80S ribosomes via the hSKI2 helicase module

The hSKI complex binds the 80S ribosome via evolutionarily conserved interactions between hSKI2 and the small ribosomal subunit (Figure 5A). The hSKI2 RecA2 domain binds between the "head" and the "shoulder" of the 40S at ribosomal proteins uS3, uS12, eS10, and rRNA helix 16. The $hSKI2_{arch}$ domain binds the "head" of the 40S at ribosomal protein uS3, uS10, and rRNA helix 41. Several THES-associated disease mutations cluster in positions that would either directly or indirectly affect 40S recognition. For example, the in-frame deletion mutant ΔR888 is in the globular KOW (Kyrpides-Ouzounis-Woese) fold that binds rRNA, while other mutations are within the stalk (the in-frame deletion ΔQ1034) or at the base of the arch (W466G) (THES-5 hotspots; Figure 5C), suggesting the integrity of the arch domain is physiologically important.

Although the positioning of hSKI close to the entry of the ribosome mRNA channel is similar to that of yeast 80S-bound Ski (Schmidt et al., 2016), there are notable differences. First, rRNA helix 16 in the "shoulder" of the 40S is in the classical straight conformation instead of the unusual bent conformation observed in the reconstruction of the yeast complex (Figures S3D and S3E) (Schmidt et al., 2016). Moreover, there is no additional interaction between hSKI and the 40S: the $hSKI8_{OUT}$ subunit is at a distance of 40 Å from the ribosomal proteins uS2, uS5, and eS21 instead of interacting directly with them as was the case for yeast $Ski8_{out}$ (Figure S3E) (Schmidt et al., 2016). Indeed, hSKI is more flexibly tethered to the 40S, rather than packing closely against it as seen in the yeast reconstruction.

In the yeast system, ribosome binding is thought to modulate the conformation of the Ski2 arch domain, thus releasing it from an autoinhibitory state (Schmidt et al., 2016). Although the arch domain of hSKI2 adopts a conformation similar to that observed in the yeast 80S-bound Ski, the same conformation is also observed in the reconstruction of hSKI in isolation (Figure S3D). We therefore conclude that binding to 80S ribosomes does not have a major influence on the hSKI2 arch domain. The differences at the structural level between the two orthologs are consistent with differences at the biochemical level. In yeast, the Ski complex in isolation has only modest ATPase activity that is significantly increased upon removal of the Ski2 arch domain (Halbach et al., 2013). In contrast, removal of the hSKI2 arch domain led only to a minor increase in the ATPase activity of hSKI in isolation (hSKI-Δarch) (Figure 4A; Table S2), suggesting that hSKI is not in a significantly autoinhibited state prior to 80S binding. Whether this reflects physiological differences between the yeast and human complexes is unclear. For example, while hSKI is thought to be exclusively bound to ribosomes (Tuck et al., 2020), yeast Ski is found in mutually exclusive 80S-bound and Ska1-bound complexes (Zhang et al., 2019) and may, accordingly, require additional regulation in this respect.

80S-bound hSKI switches to an open state when in the ATPase active form

The 80S-hSKI (with hSKI in the closed state) shows well-defined density for the ribosome-associated RNA (Figures 5A and 5B). In the structure, the CrPV-IRES RNA has the canonical features expected for the CrPV-IRES alone, with two nested pseudoknots (PK-2 and PK-3) close to the exit of the ribosome mRNA channel and another (PK-1) forming as an independent domain bound in the decoding center of the 40S (Fernández et al., 2014; Schüler et al., 2006). After the A site, the ribonucleotide chain continues until it reaches the surface of the ribosome and then becomes less defined as the RNA reaches solvent. After a distance of about 50 Å from the surface of the 80S, the RNA density becomes well-ordered again as the ribonucleotide chain enters the $hSKI2_{cat}$ core. Here, six nucleotides are bound in the same positions and with the same interactions described for the RNA-bound hSKI structure in isolation, including the interaction of the RNA 3′ end with the wedge segment of the gatekeeping module (Figure 5C). Thus, in the closed state of the 80S-hSKI complex, the RNA 3′ end is trapped in the helicase channel, and the exit site of the RNA substrate is blocked. Consistently, the RNA density also stopped inside the yeast Ski2 helicase core in the reconstruction of the compact RNase-treated yeast 80S-Ski complex (Schmidt et al., 2016).

Previous biochemical experiments have shown that the hSKI complex can extract mRNA from stalled ribosomes (Zinoviev et al., 2020). To recapitulate this scenario in our system, we reconstituted an 80S-CrPV-IRES assembly with wild-type hSKI, incubated with ATP for 15 min and collected cryo-EM data on a Titan Krios microscope (Figure S7). In the corresponding reconstruction, the interaction between 80S and hSKI2 is essentially unchanged with respect to that observed in the presence of a prehydrolytic ATP mimic. Importantly, a fraction of the particles (Figure S7G) showed no ordered density at the base of $hSKI2_{cat}$ for the entire gatekeeping module (Figure 6). Unlike hSKI in isolation, this alignment was dominated by the ribosome-bound hSKI2 in the open state, leaving the separate hSKI gatekeeping module unresolved. Thus, we could now visualize the RNA exiting from the helicase channel of hSKI in the open state (Figure 6C).

The cryo-EM reconstruction of the open state shows that the RNA in the helicase channel extends with well-defined density for three nucleotides more at the 3′ end (N7, N8, and N9) than detected in the closed state (Figure 6C). The base and sugar of ribonucleotide N7 makes polar and stacking interactions with RecA1, resulting in a twist of the ribonucleotide chain of $\sim$180°. The RNA 3′ end exits the helicase core in a bent conformation, with nucleotides N8 and N9 making only minor interactions with the ratchet helix (Figure 6D). Thus, when hSKI is in the open state as observed in the active assembly, the 3′ end of the ribosome-bound RNA traverses the helicase core and occupies the space where the wedge of the gatekeeping module

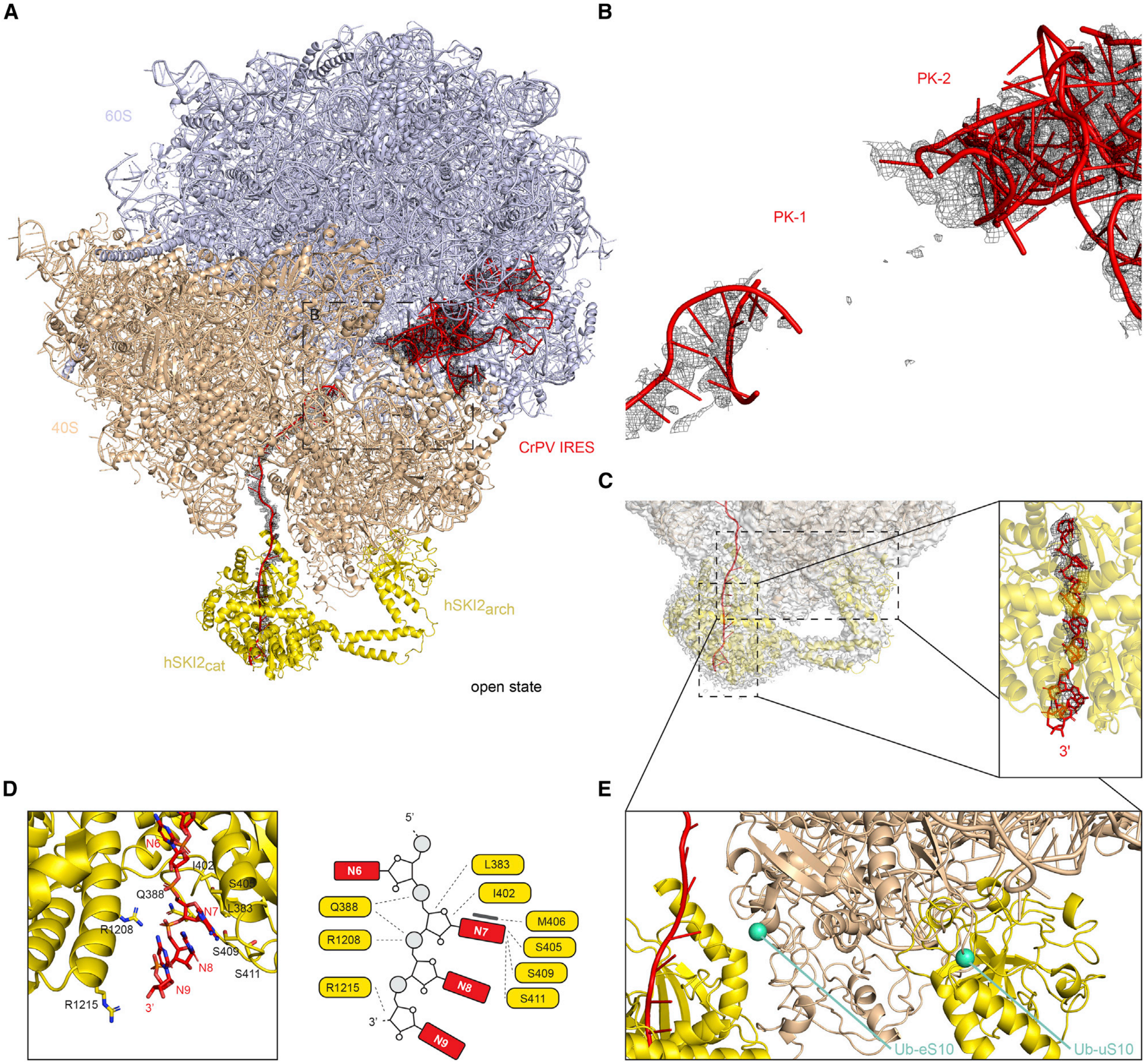

Figure 6. RNA traverses the hSKI2 helicase in an open-state hSKI-80S complex

(A) Cartoon representations of hSKI bound in an open state on a human 80S reconstituted with a CrPV IRES-29 nt RNA. Orientation and coloring as in Figure 5. Note that the gatekeeping module has detached from the helicase module.

(B) Zoom-in view of the 80S-bound RNA at CrPV IRES PK-1 and PK-2. Note that most density for PK-1 has disappeared (compare with Figure 5B), suggesting it has unfolded.

(C) Zoom-in view of the 80S-bound RNA at the 3′ end. The panel on the right shows an enlarged photo of the nine nucleot ides and the RNA 3′ end exiting from hSKI2 in open-state hSKI.

(D) Detailed interactions between hSKI2 and the additional three ordered nucleotides at the RNA 3′ end (downstream of those in Figure 4C). (Right) Zoom-in of the structure at the RNA-binding residues. (Left) Corresponding schematic of the interactions.

(E) N-terminal ubiquitination of eS10 and uS10 ribosomal proteins clashes with binding to the hSKI helicase module. Sites of ubiquitination are indicated as green spheres.

See also Figure S7; Table S1.

was bound in the closed state. Concomitantly, the density for the IRES structure disappears, suggesting that the PKs unfold as the ribosome-bound RNA is extracted and ejected by the hSKI2 helicase (Figure 6B).

The data are consistent with a model whereby the hSKI gatekeeping module detaches when the most 3′ end nucleotide traverses the helicase channel in an ATPase-dependent manner and stays open as the entire ribonucleotide chain is

threaded through, all in an ATPase-dependent manner. The model predicts that the removal of the wedge segment would only impact the initial opening step. Indeed, removal of the wedge segment in the hSKI-Δwedge mutant does not significantly affect the overall ATPase activity of the complex (Figure 4A; Table S2).

Open-state hSKI supports a conserved helicase-exosome RNA channeling mode

The RNA-binding mode observed in the open state of hSKI and, in particular, the bent conformation, with which the ribonucleotide chain traverses the RNA exit channel of hSKI2, are similar in conformation to those observed in the structure of the exosome-bound MTR4 (Weick et al., 2018). These observations suggest that the detached RNA-bound hSKI2 helicase may channel RNA into the cytoplasmic exosome in a manner similar to that observed in the case of MTR4 and the nuclear exosome (Gerlach et al., 2018; Schuller et al., 2018; Weick et al., 2018). To test this prediction, we first biochemically reconstituted the components of the human cytoplasmic exosome (Figure S7A). We purified the inactive 9-subunit core of the human exosome (hEXO9) as previously described for the nuclear complex (Gerlach et al., 2018; Weick et al., 2018). To incorporate the cytoplasmic-specific ribonuclease, we used a human-cell expression system and purified recombinant full-length hDIS3L (with the inactivating D486N mutation). The corresponding 10-subunit cytoplasmic exosome (hEXO10_c) binds HBS1L3, a spliced variant of HBS1-like that interacts with hEXO10_c in a similar way as the yeast Ski7 protein interacts with yExo10_c (Kalisiak et al., 2017; Kowalinski et al., 2016). Using size-exclusion chromatography (SEC) experiments, we demonstrated that HBS1L3 also binds hSKI and is required to bridge the interaction between hSKI and hEXO10_c (Figure 7A).

With these purified samples, we performed RNase protection assays (Figure 7B), similar to those we used previously to analyze the RNA-binding properties of exosome complexes (Bonneau et al., 2009; Gerlach et al., 2018). Briefly, a body-labeled, single-stranded RNA was incubated with (catalytically inactive) exosome complexes and treated with benzonase. The footprint (e.g., the size of the RNA fragments that upon protein binding become protected from RNase digestion) was analyzed by denaturing polyacrylamide gel electrophoresis (PAGE) (Figure 7B). The cytoplasmic ribonuclease core complex hEXO10_c gave rise to a characteristic footprint (Figure 7B, lane 1) similar to that previously observed for its nuclear counterpart, hEXO10_n (Gerlach et al., 2018). In the case of the cytoplasmic human exosome, removal of the arch domain was required to observe the increased RNA footprint of the holocomplex with respect to the ribonuclease core (Figure 7B, compare lanes 2 and 4), similarly to its yeast counterpart (Halbach et al., 2013). Remarkably, the difference in footprint between the core and holocomplexes of the cytoplasmic exosome (Figure 7B) is similar to that of the nuclear exosome (Gerlach et al., 2018). Notwithstanding the additional regulatory role of the hSKI2 arch domain, the nuclear and cytoplasmic holoexosomes thus embed a continuous RNA channel of similar length, supporting the notion of a conserved mode of interaction and a conserved channeling mechanism (Figure 7C).

CONCLUSIONS

The hSKI complex undergoes a major remodeling when switching from a closed state to an open state in which the helicase is active and released from the rest of the complex. This large-scale conformational change is underpinned by the structural organization of hSKI into two individual but connected modules: a helicase module and a gatekeeping module. The gatekeeping module can swing open or close toward the helicase module. In the following model, when the hSKI complex is inactive, the gate is closed and binds the helicase module via extensive intermolecular interactions made with the basal surface of the helicase domain. In this closed conformation, the wedge segment in the gate binds and occludes the exit of the helicase channel, enclosing the 3′ end of a bound RNA substrate. Upon activation, that is, in the context of RNA-dependent ATP hydrolysis of 80S-bound hSKI2, the gate opens and detaches from the helicase module, which remains bound to the ribosome. The open state of hSKI is also marked by a movement of the wedge segment, which dissociates from the exit channel of hSKI2, thereby allowing the RNA 3′ end to exit and the ribosome-bound mRNA to be extracted. The efficient interconversion between the large-amplitude conformational changes of the closed and open states is likely due to the covalent tethering of the regulatory and catalytic modules, which can thus remain in proximity even when they are detached from each other.

In contrast to the yeast system, the interaction between hSKI and the 40S ribosomal subunit is exclusively mediated by hSKI2 and is rather flexible in character. The positioning of hSKI2 on the ribosome in the cryo-EM structure explains how it can access a short 3′ overhang of a bound mRNA, a substrate that is expected to arise upon endonucleolytic cleavage after ribosome stalling (Schmidt et al., 2016; Tuck et al., 2020; Zinoviev et al., 2020). The minimal length of the 3′ overhang determined in biochemical assays (Zinoviev et al., 2020) is required to span the distance between the ribosome and the helicase core of hSKI2. However, the SKI complex can also efficiently extract ribosome-bound mRNAs with long overhanging 3′-terminal regions (Zinoviev et al., 2020). The finding that hSKI is flexible when attached to the 80S rationalizes how it can be reached by longer and flexible 3′ overhangs. The position of hSKI in the 80S-bound structure has also interesting repercussions for surveillance mechanisms. The hSKI2 interaction with ribosomal proteins eS10 and uS10 would not be compatible with the ubiquitination of these subunits (Figure 6E), as has been reported to occur on colliding ribosomes (Ikeuchi et al., 2019; Juszkiewicz et al., 2018; Winz et al., 2019).

The gatekeeping mechanism of hSKI does not have a significant inhibitory effect on the ATPase properties of the complex. Once the gate opens, when the most 3′ nucleotide traverses the helicase channel, it stays open until the extraction process is concluded. The arch domain of the helicase also does not have a significant autoinhibitory effect on the ATPase properties of hSKI and, consistently, its conformation is not modulated upon 80S binding, in contrast to what was observed for the yeast ortholog (Schmidt et al., 2016). However, the gatekeeping structure and the arch domain appear to serve an evolutionarily conserved role in regulating RNA channeling to the exosome

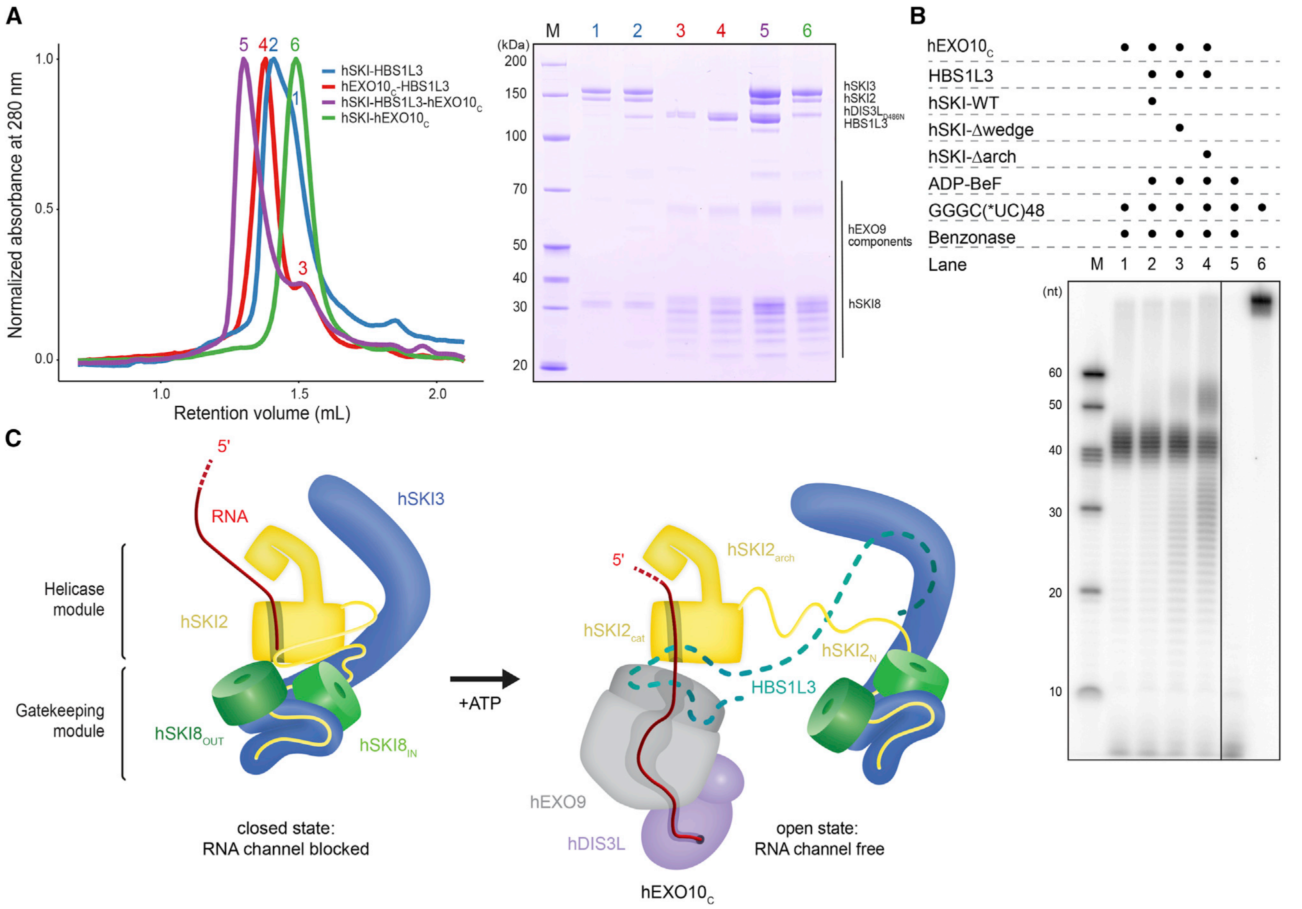

Figure 7. RNA channeling of hSKI to the cytoplasmic exosome

(A) Physical interaction of hSKI and the human cytoplasmic exosome. Size-exclusion chromatography (SEC) assays were carried out with hDIS3L$_{D486N}$ catalytic inactive mutant. The panel on the left is an overlay of the chromatograms, and the panel on the right a Coomassie-stained 4%–12% SDS-PAGE analyzing the peak fractions (as numbered and colored on the left). Note that in the hSKI-HBS1L3 (blue curve) and in the hEXO10$_C$-HBS1L3 (red curve) samples hSKI and hEXO10$_C$ were loaded in excess to control for differences between HBS1L3-bound and -unbound complexes.

(B) RNA path assessed by RNase protection assays of human SKI wild-type and mutants in the context of the human cytoplasmic exosome (human EXO10$_C$ and human HBS1L3). A single-stranded C(*UC)$_{48}$ RNA internally labeled with ^{32}P at the uridine 5′-phosphate was incubated with proteins and nucleotides as indicated and treated with benzonase. The reaction products were analyzed by denaturing urea-PAGE. Note the increase in length of the RNA fragments when the cytoplasmic exosome was incubated with hSKI-Δarch, similar to the increase in length observed in the case of hMTR4 and the nuclear exosome complex (Gerlach et al., 2018). An SDS-PAGE of the proteins and complexes used in this assay is shown in Figure S7A.

(C) Model of the RNA channeling mechanism of the cytoplasmic SKI-exosome holocomplex compatible with the RNA path from (A). In this model, channeling is achieved by hSKI2 in the open-state conformation of hSKI with a similar overall path as observed for the nuclear exosome holocomplex (Gerlach et al., 2018; Schuller et al., 2018; Weick et al., 2018). Note that in context of the wild type, we expect that ATP hydrolysis is required in addition to ATP binding for transition to the hSKI open state and channeling to the exosome.

See also Figure S7.

for degradation (see also Halbach et al., 2013). The current biochemical and structural data suggest that the gatekeeping module dissociates from the hSKI2 helicase module to allow the RNA to progress to the cytoplasmic exosome core with a similar channeling path observed for the nuclear exosome holocomplex. In the context of the nuclear 14-subunit exosome holocomplex, it is likely that a similar gatekeeping mechanism is at play in hMTR4 co-factor complexes. The NEXT (hMTR4-ZCCHC8-RBM7) complex, an assembly that targets noncoding RNAs that are generated upon faulty transcription processes, is a prime candidate for such regulation: the C-terminal domain of hZCCHC8 binds the basal surface of the hMTR4 helicase (Puno and Lima, 2018) and positions Phe673 at the exit of the helicase channel at the equivalent position of hSKI2 W146 (Figure S5H). The similarities suggest parallel evolution of conserved regulatory mechanisms of exosome-associated Ski2-like helicases in different cellular compartments.

Limitations of the study

Conceptual and technical limitations pertain to the ability to further dissect the role of the Trp-containing wedge in a meaningful manner. First, the Trp-RNA interaction is one of the many

RNA-binding interactions in the complex and by itself is unlikely to contribute significant in terms of K_d in biophysical measurements. Second, the wedge segment would only impact the initial opening step and as such it is not possible to measure the impact in bulk ATPase assays. Another limitation is that we cannot assign the exact nucleotides bound in hSKI2 when not using homopolymeric sequences (e.g., in the ribosome-bound structures) nor be sure of the identity of the 3′ end nucleotide. A further limitation is the multiple-days-long time used for the sample preparation in case of the ribosome-bound cryo-EM samples. Over this rather long processing time span, we cannot exclude potential dissociation of hSKI from the ribosome or reversal to the hSKI closed form.

STAR★METHODS

Detailed methods are provided in the online version of this paper and include the following:

- KEY RESOURCES TABLE
- RESOURCE AVAILABILITY
  - Lead contact
  - Materials availability
  - Data and code availability
- EXPERIMENTAL MODEL AND SUBJECT DETAILS
- METHOD DETAILS
  - Cloning, protein expression and purification
  - Ribosome subunit purification
  - *In vitro* transcription of RNA substrates
  - ATPase activity assays
  - Cytoplasmic exosome reconstitution
  - Size exclusion chromatography assays
  - RNase protection assays
  - Cryo-EM sample preparation
  - Cryo-EM data collection and processing
  - Density interpretation and model building
- QUANTIFICATION AND STATISTICAL ANALYSIS

SUPPLEMENTAL INFORMATION

Supplemental information can be found online at https://doi.org/10.1016/j.molcel.2022.01.009.

ACKNOWLEDGMENTS

We are grateful to Daniel Bollschweiler and Tillman Schäfer at the Max Planck Institute of Biochemistry (MPIB) cryo-EM facility for help with EM data collection and the MPIB core facilities for support. We thank Christian Benda and J. Rajan Prabu for maintenance and development of computational infrastructure for EM data processing, Courtney Long for help with the preparation of the manuscript, and members of the lab for discussions. This work was supported by funding from the Max-Planck-Gesellschaft, the European Commission (ERC Advanced Investigator Grant EXORICO), and the Deutsche Forschungsgemeinschaft (DFG SFB1035, GRK1721, SFB/TRR 237) to E.C.

AUTHOR CONTRIBUTIONS

E.C. and A. Kögel initiated the project; A. Kögel, A. Keidel, and F.B. designed and performed experiments; I.B.S. supervised the cryo-EM analyses; E.C., A. Kögel, A. Keidel, and I.B.S. wrote the manuscript.

DECLARATION OF INTERESTS

E.C. is a member of the *Molecular Cell* advisory board.

Received: September 24, 2021
Revised: December 1, 2021
Accepted: January 7, 2022
Published: February 3, 2022

REFERENCES

Afonine, P.V., Klaholz, B.P., Moriarty, N.W., Poon, B.K., Sobolev, O.V., Terwilliger, T.C., Adams, P.D., and Urzhumtsev, A. (2018a). New tools for the analysis and validation of cryo-EM maps and atomic models. Acta Crystallogr. D Struct. Biol. *74*, 814–840. https://doi.org/10.1107/S2059798318009324.

Afonine, P.V., Poon, B.K., Read, R.J., Sobolev, O.V., Terwilliger, T.C., Urzhumtsev, A., and Adams, P.D. (2018b). Real-space refinement in PHENIX for cryo-EM and crystallography. Acta Crystallogr. D Struct. Biol. *74*, 531–544. https://doi.org/10.1107/S2059798318006551.

Anderson, J.S., and Parker, R.P. (1998). The 3′ to 5′ degradation of yeast mRNAs is a general mechanism for mRNA turnover that requires the SKI2 DEVH box protein and 3′ to 5′ exonucleases of the exosome complex. EMBO J *17*, 1497–1506. https://doi.org/10.1093/emboj/17.5.1497.

Araki, Y., Takahashi, S., Kobayashi, T., Kajiho, H., Hoshino, S., and Katada, T. (2001). Ski7p G protein interacts with the exosome and the Ski complex for 3″-to-5″ mRNA decay in yeast. EMBO J *20*, 4684–4693. https://doi.org/10.1093/emboj/20.17.4684.

Bernstein, J., Ballin, J.D., Patterson, D.N., Wilson, G.M., and Toth, E.A. (2010). Unique properties of the Mtr4p-poly(A) complex suggest a role in substrate targeting. Biochemistry *49*, 10357–10370. https://doi.org/10.1021/bi101518x.

Bieniossek, C., Imasaki, T., Takagi, Y., and Berger, I. (2012). MultiBac: expanding the research toolbox for multiprotein complexes. Trends Biochem. Sci. *37*, 49–57. https://doi.org/10.1016/j.tibs.2011.10.005.

Biyani, N., Righetto, R.D., McLeod, R., Caujolle-Bert, D., Castano-Diez, D., Goldie, K.N., and Stahlberg, H. (2017). Focus: the interface between data collection and data processing in cryo-EM. J. Struct. Biol. *198*, 124–133. https://doi.org/10.1016/j.jsb.2017.03.007.

Bonneau, F., Basquin, J., Ebert, J., Lorentzen, E., and Conti, E. (2009). The yeast exosome functions as a macromolecular cage to channel RNA substrates for degradation. Cell *139*, 547–559. https://doi.org/10.1016/j.cell.2009.08.042.

Brown, J.T., Bai, X., and Johnson, A.W. (2000). The yeast antiviral proteins Ski2p, Ski3p, and Ski8p exist as a complex *in vivo*. RNA 6, 449–457.

Büttner, K., Nehring, S., and Hopfner, K.-P. (2007). Structural basis for DNA duplex separation by a superfamily-2 helicase. Nat. Struct. Mol. Biol. *14*, 647–652. https://doi.org/10.1038/nsmb1246.

Chen, V.B., Arendall, W.B., Headd, J.J., Keedy, D.A., Immormino, R.M., Kapral, G.J., Murray, L.W., Richardson, J.S., and Richardson, D.C. (2010). MolProbity: all-atom structure validation for macromolecular crystallography. Acta Crystallogr. D Biol. Crystallogr. *66*, 12–21. https://doi.org/10.1107/S0907444909042073.

Chlebowski, A., Lubas, M., Jensen, T.H., and Dziembowski, A. (2013). RNA decay machines: the exosome. Biochim. Biophys. Acta *1829*, 552–560. https://doi.org/10.1016/j.bbagrm.2013.01.006.

Dobrev, N., Ahmed, Y.L., Sivadas, A., Soni, K., Fischer, T., and Sinning, I. (2021). The zinc-finger protein Red1 orchestrates MTREC submodules and binds the Mtl1 helicase arch domain. Nat. Commun. *12*, 3456. https://doi.org/10.1038/s41467-021-23565-3.

Dziembowski, A., Lorentzen, E., Conti, E., and Séraphin, B. (2007). A single subunit, Dis3, is essentially responsible for yeast exosome core activity. Nat. Struct. Mol. Biol. *14*, 15–22. https://doi.org/10.1038/nsmb1184.

Eckard, S.C., Rice, G.I., Fabre, A., Badens, C., Gray, E.E., Hartley, J.L., Crow, Y.J., and Stetson, D.B. (2014). The SKIV2L RNA exosome limits activation of

the RIG-I-like receptors. Nat. Immunol. *15*, 839–845. https://doi.org/10.1038/ni.2948.

Emsley, P., Lohkamp, B., Scott, W.G., and Cowtan, K. (2010). Features and development of coot. Acta Crystallogr. D Biol. Crystallogr. *66*, 486–501. https://doi.org/10.1107/S0907444910007493.

Fabre, A., Charroux, B., Martinez-Vinson, C., Roquelaure, B., Odul, E., Sayar, E., Smith, H., Colomb, V., Andre, N., Hugot, J.-P., et al. (2012). SKIV2L mutations cause syndromic diarrhea, or trichohepatoenteric syndrome. Am. J. Hum. Genet. *90*, 689–692. https://doi.org/10.1016/j.ajhg.2012.02.009.

Fabre, A., Martinez-Vinson, C., Roquelaure, B., Missirian, C., Andre, N., Breton, A., Lachaux, A., Odul, E., Colomb, V., Lemale, J., et al. (2011). Novel mutations in TTC37 associated with tricho-hepato-enteric syndrome. Hum. Mutat. *32*, 277–281. https://doi.org/10.1002/humu.21420.

Fabre, A., Martinez-Vinson, C., Goulet, O., and Badens, C. (2013). Syndromic diarrhea/tricho-hepato-enteric syndrome. Orphanet J. Rare Dis. *8*, 5. https://doi.org/10.1186/1750-1172-8-5.

Falk, S., Tants, J.-N., Basquin, J., Thoms, M., Hurt, E., Sattler, M., and Conti, E. (2017). Structural insights into the interaction of the nuclear exosome helicase Mtr4 with the pre-ribosomal protein Nop53. RNA *23*, 1780–1787. https://doi.org/10.1261/rna.062901.117.

Falk, S., Weir, J.R., Hentschel, J., Reichelt, P., Bonneau, F., and Conti, E. (2014). The molecular architecture of the TRAMP complex reveals the organization and interplay of its two catalytic activities. Mol. Cell *55*, 856–867. https://doi.org/10.1016/j.molcel.2014.07.020.

Fernández, I.S., Bai, X.-C., Murshudov, G., Scheres, S.H.W., and Ramakrishnan, V. (2014). Initiation of translation by cricket paralysis virus IRES requires its translocation in the ribosome. Cell *157*, 823–831. https://doi.org/10.1016/j.cell.2014.04.015.

Gerlach, P., Schuller, J.M., Bonneau, F., Basquin, J., Reichelt, P., Falk, S., and Conti, E. (2018). Distinct and evolutionary conserved structural features of the human nuclear exosome complex. Elife *7*, e38686. https://doi.org/10.7554/eLife.38686.

Goddard, T.D., Huang, C.C., Meng, E.C., Pettersen, E.F., Couch, G.S., Morris, J.H., and Ferrin, T.E. (2018). UCSF ChimeraX: meeting modern challenges in visualization and analysis. Protein Sci *27*, 14–25. https://doi.org/10.1002/pro.3235.

Halbach, F., Reichelt, P., Rode, M., and Conti, E. (2013). The yeast ski complex: crystal structure and RNA channeling to the exosome complex. Cell *154*, 814–826. https://doi.org/10.1016/j.cell.2013.07.017.

Halbach, F., Rode, M., and Conti, E. (2012). The crystal structure of *S. cerevisiae* Ski2, a DExH helicase associated with the cytoplasmic functions of the exosome. RNA *18*, 124–134. https://doi.org/10.1261/rna.029553.111.

Hellen, C.U.T. (2009). IRES-induced conformational changes in the ribosome and the mechanism of translation initiation by internal ribosomal entry. Biochim. Biophys. Acta *1789*, 558–570. https://doi.org/10.1016/j.bbagrm.2009.06.001.

Hoh, S.W., Burnley, T., and Cowtan, K. (2020). Current approaches for automated model building into cryo-EM maps using Buccaneer with CCP-EM. Acta Crystallogr. D Struct. Biol. *76*, 531–541. https://doi.org/10.1107/S2059798320005513.

Ikeuchi, K., Tesina, P., Matsuo, Y., Sugiyama, T., Cheng, J., Saeki, Y., Tanaka, K., Becker, T., Beckmann, R., and Inada, T. (2019). Collided ribosomes form a unique structural interface to induce Hel2-driven quality control pathways. EMBO J *38*, e100276. https://doi.org/10.15252/embj.2018100276.

Jackson, R.N., Klauer, A.A., Hintze, B.J., Robinson, H., van Hoof, A., and Johnson, S.J. (2010). The crystal structure of Mtr4 reveals a novel arch domain required for rRNA processing. EMBO J *29*, 2205–2216. https://doi.org/10.1038/emboj.2010.107.

Januszyk, K., and Lima, C.D. (2014). The eukaryotic RNA exosome. Curr. Opin. Struct. Biol. *24*, 132–140. https://doi.org/10.1016/j.sbi.2014.01.011.

Juszkiewicz, S., Chandrasekaran, V., Lin, Z., Kraatz, S., Ramakrishnan, V., and Hegde, R.S. (2018). ZNF598 is a quality control sensor of collided ribosomes. Mol. Cell *72*, 469–481.e7. https://doi.org/10.1016/j.molcel.2018.08.037.

Kalisiak, K., Kuliński, T.M., Tomecki, R., Cysewski, D., Pietras, Z., Chlebowski, A., Kowalska, K., and Dziembowski, A. (2017). A short splicing isoform of HBS1L links the cytoplasmic exosome and SKI complexes in humans. Nucleic Acids Res *45*, 2068–2080. https://doi.org/10.1093/nar/gkw862.

Khatter, H., Myasnikov, A.G., Natchiar, S.K., and Klaholz, B.P. (2015). Structure of the human 80S ribosome. Nature *520*, 640–645. https://doi.org/10.1038/nature14427.

Khemici, V., and Linder, P. (2018). RNA helicases in RNA decay. Biochem. Soc. Trans. *46*, 163–172. https://doi.org/10.1042/BST20170052.

Kowalinski, E., Kögel, A., Ebert, J., Reichelt, P., Stegmann, E., Habermann, B., and Conti, E. (2016). Structure of a cytoplasmic 11-subunit RNA exosome complex. Mol. Cell *63*, 125–134. https://doi.org/10.1016/j.molcel.2016.05.028.

Lee, W.S., Teo, K.M., Ng, R.T., Chong, S.Y., Kee, B.P., and Chua, K.H. (2016). Novel mutations in SKIV2L and TTC37 genes in Malaysian children with trichohepatoenteric syndrome. Gene *586*, 1–6. https://doi.org/10.1016/j.gene.2016.03.049.

Li, X., Burnight, E.R., Cooney, A.L., Malani, N., Brady, T., Sander, J.D., Staber, J., Wheelan, S.J., Joung, J.K., McCray, P.B., et al. (2013). piggyBac transposase tools for genome engineering. Proc. Natl. Acad. Sci. USA *110*, E2279–E2287. https://doi.org/10.1073/pnas.1305987110.

Liebschner, D., Afonine, P.V., Baker, M.L., Bunkóczi, G., Chen, V.B., Croll, T.I., Hintze, B., Hung, L.-W., Jain, S., McCoy, A.J., et al. (2019). Macromolecular structure determination using X-rays, neutrons and electrons: recent developments in Phenix. Acta Crystallogr. D Struct. Biol. *75*, 861–877. https://doi.org/10.1107/S2059798319011471.

Lingaraju, M., Johnsen, D., Schlundt, A., Langer, L.M., Basquin, J., Sattler, M., Heick Jensen, T., Falk, S., and Conti, E. (2019a). The MTR4 helicase recruits nuclear adaptors of the human RNA exosome using distinct arch-interacting motifs. Nat. Commun. *10*, 3393. https://doi.org/10.1038/s41467-019-11339-x.

Lingaraju, M., Schuller, J.M., Falk, S., Gerlach, P., Bonneau, F., Basquin, J., Benda, C., and Conti, E. (2019b). To process or to decay: a mechanistic view of the nuclear RNA exosome. Cold Spring Harb. Symp. Quant. Biol. *84*, 155–163. https://doi.org/10.1101/sqb.2019.84.040295.

Liu, Q., Greimann, J.C., and Lima, C.D. (2006). Reconstitution, activities, and structure of the eukaryotic RNA exosome. Cell *127*, 1223–1237. https://doi.org/10.1016/j.cell.2006.10.037.

Mitchell, P., Petfalski, E., Shevchenko, A., Mann, M., and Tollervey, D. (1997). The exosome: a conserved eukaryotic RNA processing complex containing multiple 3″→5″ exoribonucleases. Cell 91, 457–466.

Olsen, K.J., and Johnson, S.J. (2021). Mtr4 RNA helicase structures and interactions. Biol. Chem. *402*, 605–616. https://doi.org/10.1515/hsz-2020-0329.

Ozgur, S., Buchwald, G., Falk, S., Chakrabarti, S., Prabu, J.R., and Conti, E. (2015). The conformational plasticity of eukaryotic RNA-dependent ATPases. FEBS J *282*, 850–863. https://doi.org/10.1111/febs.13198.

Perez-Riba, A., and Itzhaki, L.S. (2019). The tetratricopeptide-repeat motif is a versatile platform that enables diverse modes of molecular recognition. Curr. Opin. Struct. Biol. *54*, 43–49. https://doi.org/10.1016/j.sbi.2018.12.004.

Petrov, A., Grosely, R., Chen, J., O'Leary, S.E., and Puglisi, J.D. (2016). Multiple parallel pathways of translation initiation on the CrPV IRES. Mol. Cell *62*, 92–103. https://doi.org/10.1016/j.molcel.2016.03.020.

Pettersen, E.F., Goddard, T.D., Huang, C.C., Couch, G.S., Greenblatt, D.M., Meng, E.C., and Ferrin, T.E. (2004). UCSF Chimera—a visualization system for exploratory research and analysis. J. Comput. Chem. *25*, 1605–1612. https://doi.org/10.1002/jcc.20084.

Pisarev, A.V., Unbehaun, A., Hellen, C.U.T., and Pestova, T.V. (2007). Assembly and analysis of eukaryotic translation initiation complexes. Methods Enzymol *430*, 147–177. https://doi.org/10.1016/S0076-6879(07)30007-4.

Puno, M.R., and Lima, C.D. (2018). Structural basis for MTR4-ZCCHC8 interactions that stimulate the MTR4 helicase in the nuclear exosome-targeting

complex. Proc. Natl. Acad. Sci. USA *115*, E5506–E5515. https://doi.org/10.1073/pnas.1803530115.

Rosenthal, P.B., and Henderson, R. (2003). Optimal determination of particle orientation, absolute hand, and contrast loss in single-particle electron cryomicroscopy. J. Mol. Biol. *333*, 721–745. https://doi.org/10.1016/j.jmb.2003.07.013.

Schaeffer, D., and van Hoof, A. (2011). Different nuclease requirements for exosome- mediated degradation of normal and nonstop mRNAs. Proc. Natl. Acad. Sci. USA *108*, 2366–2371. https://doi.org/10.1073/pnas.1013180108.

Schmid, M., and Jensen, T.H. (2019). The nuclear RNA exosome and its cofactors. Adv. Exp. Med. Biol. *1203*, 113–132. https://doi.org/10.1007/978-3-030-31434-7_4.

Schmidt, C., Kowalinski, E., Shanmuganathan, V., Defenouillère, Q., Braunger, K., Heuer, A., Pech, M., Namane, A., Berninghausen, O., Fromont-Racine, M., et al. (2016). The cryo-EM structure of a ribosome-Ski2-Ski3-Ski8 helicase complex. Science *354*, 1431–1433. https://doi.org/10.1126/science.aaf7520.

Schneider, C.A., Rasband, W.S., and Eliceiri, K.W. (2012). NIH Image to ImageJ: 25 years of image analysis. Nat. Methods *9*, 671–675. https://doi.org/10.1038/nmeth.2089.

Schorb, M., Haberbosch, I., Hagen, W.J.H., Schwab, Y., and Mastronarde, D.N. (2019). Software tools for automated transmission electron microscopy. Nat. Methods *16*, 471–477. https://doi.org/10.1038/s41592-019-0396-9.

Schüler, M., Connell, S.R., Lescoute, A., Giesebrecht, J., Dabrowski, M., Schroeer, B., Mielke, T., Penczek, P.A., Westhof, E., and Spahn, C.M.T. (2006). Structure of the ribosome-bound cricket paralysis virus IRES RNA. Nat. Struct. Mol. Biol. *13*, 1092–1096. https://doi.org/10.1038/nsmb1177.

Schuller, J.M., Falk, S., Fromm, L., Hurt, E., and Conti, E. (2018). Structure of the nuclear exosome captured on a maturing preribosome. Science *360*, 219–222. https://doi.org/10.1126/science.aar5428.

Stirnimann, C.U., Petsalaki, E., Russell, R.B., and Müller, C.W. (2010). WD40 proteins propel cellular networks. Trends Biochem. Sci. *35*, 565–574. https://doi.org/10.1016/j.tibs.2010.04.003.

Synowsky, S.A., and Heck, A.J.R. (2008). The yeast Ski complex is a hetero-tetramer. Protein Sci *17*, 119–125. https://doi.org/10.1110/ps.073155908.

Thoms, M., Thomson, E., Baßler, J., Gnädig, M., Griesel, S., and Hurt, E. (2015). The exosome is recruited to RNA substrates through specific adaptor proteins. Cell *162*, 1029–1038. https://doi.org/10.1016/j.cell.2015.07.060.

Toh-E, A., Guerry, P., and Wickner, R.B. (1978). Chromosomal superkiller mutants of *Saccharomyces cerevisiae*. J. Bacteriol. *136*, 1002–1007.

Tomecki, R., Kristiansen, M.S., Lykke-Andersen, S., Chlebowski, A., Larsen, K.M., Szczesny, R.J., Drazkowska, K., Pastula, A., Andersen, J.S., Stepien, P.P., et al. (2010). The human core exosome interacts with differentially localized processive RNaseS: hDIS3 and hDIS3L. EMBO J *29*, 2342–2357. https://doi.org/10.1038/emboj.2010.121.

Tuck, A.C., Rankova, A., Arpat, A.B., Liechti, L.A., Hess, D., Iesmantavicius, V., Castelo-Szekely, V., Gatfield, D., and Bühler, M. (2020). Mammalian RNA decay pathways are highly specialized and widely linked to translation. Mol. Cell *77*, 1222–1236.e13. https://doi.org/10.1016/j.molcel.2020.01.007.

Tunyasuvunakool, K., Adler, J., Wu, Z., Green, T., Zielinski, M., Žídek, A., Bridgland, A., Cowie, A., Meyer, C., Laydon, A., et al. (2021). Highly accurate protein structure prediction for the human proteome. Nature *596*, 590–596. https://doi.org/10.1038/s41586-021-03828-1.

Wang, J., Chen, J., Wu, G., Zhang, H., Du, X., Chen, S., Zhang, L., Wang, K., Fan, J., Gao, S., et al. (2019). NRDE2 negatively regulates exosome functions by inhibiting MTR4 recruitment and exosome interaction. Genes Dev *33*, 536–549. https://doi.org/10.1101/gad.322602.118.

Weick, E.-M., and Lima, C.D. (2021). RNA helicases are hubs that orchestrate exosome-dependent 3″-5″ decay. Curr. Opin. Struct. Biol. *67*, 86–94. https://doi.org/10.1016/j.sbi.2020.09.010.

Weick, E.-M., Puno, M.R., Januszyk, K., Zinder, J.C., DiMattia, M.A., and Lima, C.D. (2018). Helicase-dependent RNA decay illuminated by a cryo-EM structure of a human nuclear RNA exosome-MTR4 complex. Cell *173*, 1663–1677.e21. https://doi.org/10.1016/j.cell.2018.05.041.

Weir, J.R., Bonneau, F., Hentschel, J., and Conti, E. (2010). Structural analysis reveals the characteristic features of Mtr4, a DExH helicase involved in nuclear RNA processing and surveillance. Proc. Natl. Acad. Sci. USA *107*, 12139–12144. https://doi.org/10.1073/pnas.1004953107.

Winz, M.L., Peil, L., Turowski, T.W., Rappsilber, J., and Tollervey, D. (2019). Molecular interactions between Hel2 and RNA supporting ribosome-associated quality control. Nat. Commun. *10*, 563. https://doi.org/10.1038/s41467-019-08382-z.

Zhang, E., Khanna, V., Dacheux, E., Namane, A., Doyen, A., Gomard, M., Turcotte, B., Jacquier, A., and Fromont-Racine, M. (2019). A specialised SKI complex assists the cytoplasmic RNAexosome in the absence of direct association with ribosomes. EMBO J *38*, e100640. https://doi.org/10.15252/embj.2018100640.

Zhang, K. (2016). Gctf: real-time CTF determination and correction. J. Struct. Biol. *193*, 1–12. https://doi.org/10.1016/j.jsb.2015.11.003.

Zheng, S.Q., Palovcak, E., Armache, J.-P., Verba, K.A., Cheng, Y., and Agard, D.A. (2017). MotionCor2: anisotropic correction of beam-induced motion for improved cryo-electron microscopy. Nat. Methods *14*, 331–332. https://doi.org/10.1038/nmeth.4193.

Zinoviev, A., Ayupov, R.K., Abaeva, I.S., Hellen, C.U.T., and Pestova, T.V. (2020). Extraction of mRNA from stalled ribosomes by the ski complex. Mol. Cell *77*, 1340–1349.e6. https://doi.org/10.1016/j.molcel.2020.01.011.

Zivanov, J., Nakane, T., Forsberg, B.O., Kimanius, D., Hagen, W.J., Lindahl, E., and Scheres, S.H. (2018). New tools for automated high-resolution cryo-EM structure determination in RELION-3. Elife *7*, e42166. https://doi.org/10.7554/eLife.42166.

STAR★METHODS

KEY RESOURCES TABLE

REAGENT or RESOURCE	SOURCE	IDENTIFIER
Bacterial and virus strains		
Escherichia coli BL21 (DE3) STAR pRARE	EMBL Heidelberg Core Facility	N/A
Chemicals, peptides, and recombinant proteins		
Sf-900 II SFM medium	Thermo Fisher Scientific	10902096
FreeStyle 293 Expression Medium	Thermo Fisher Scientific	12338018
Polyethylenimine	Polysciences, Inc.	23966
cOmplete protease inhibitor	Roche	5056489001
NuPAGE 4-12% Bis-Tris protein gels	Thermo Fisher Scientific	NP0336BOX
H. sapiens SKI2-SKI3-SKI8 and derivative complexes	This study	N/A
H. sapiens HBS1L3	This study	N/A
H. sapiens hEXO9	Kowalinski et al., 2016	N/A
H. sapiens $DIS3L_{D486N}$	This study	N/A
H. sapiens 40S/60S ribosomal subunits	This study	N/A
T7 RNA Polymerase	MPI Biochemistry Core Facility	N/A
ATP, CTP, GTP, UTP	Jena Bioscience	NU-1010 – 1013
[α^{32}P] UTP	PerkinElmer	NEG007X250UC
DNase I	Roche	4716728001
Benzonase	Merck	71206
ATP	Sigma-Aldrich	A3377
ADP	Sigma-Aldrich	A2754
NADH	Sigma-Aldrich	N4505
Pyruvate kinase/Lactate dehydrogenase	Sigma-Aldrich	P0294
Phosphoenolpyruvate	Sigma-Aldrich	P0564
poly(U) RNA	Sigma-Aldrich	P9528
n-octyl-ß-D-glucoside	Sigma-Aldrich	850511P
BS_3 (bis(sulfosuccinimidyl)suberate)	Thermo Fisher Scientific	A39266
Deposited data		
apo open hSKI cryoEM map and model	This paper	EMD-13925; PDB: 7QDS
apo closed hSKI cryoEM map and model	This paper	EMD-13923; PDB: 7QDR
RNA-bound closed hSKI cryoEM map and model	This paper	EMD-13927; PDB: 7QDY
ribosome-bound closed hSKI complex cryoEM map and model	This paper	EMD-13928; PDB: 7QDZ
ribosome-bound open hSKI complex cryoEM map and model	This paper	EMD-13929; PDB: 7QE0
Raw and analyzed data	This paper	Mendeley doi:10.17632/9w78m35s2v.1
Experimental models: Cell lines		
Spodoptera frugiperda 21 (*Sf21*)	Thermo Fisher Scientific	11497013
Human embryonic kidney 293T (HEK293T)	ATCC	CRL-3216
Oligonucleotides		
GGGC(*UC)$_{48}$_T7_fwd AATTTAATACGACTCACTATA GG	Sigma-Aldrich	N/A
GGGC(*UC)$_{48}$_rev GAGAGAGAGAGAGAGAGAGAGAG AGAGAGAGAGAGAGAGAGAGAGAGAGAGAGAGAGAGA GAGAGAGAGAGAGAGAGAGAGAGAGAGA GAGAGCCCTATAGTGAGTCGTATTAAATT	Sigma-Aldrich	N/A

(*Continued on next page*)

CellPress
OPEN ACCESS

Continued

REAGENT or RESOURCE	SOURCE	IDENTIFIER
IRES29_T7_fwd TAATACGACTCACTATAGGGAAAAA TGTGATCTTGCTTGTAAATAC	Sigma-Aldrich	N/A
IRES29_rev GCGTCTTCCATGGTATCTTG	Sigma-Aldrich	N/A
25U RNA	Sigma-Aldrich	N/A
Recombinant DNA		
pACEBac1-hSKI2-10xHIS-3C-hSKI3-hSKI8 and derivative plasmids	This study	N/A
pEC-6HIS-3C-HBS1L3-$(GS)_3$-eGFP-TS	This study	N/A
pPB-TS-3C-hDIS3L_{D486N}	This study	N/A
pUC-CrPV-IRES-luciferase	This study	N/A
Software and algorithms		
SerialEM	Schorb et al., 2019	https://bio3d.colorado.edu/SerialEM/
Focus	Biyani et al., 2017	https://www.focus-em.org
MotionCor2	Zheng et al., 2017	https://msg.ucsf.edu/software/
Gctf	Zhang, 2016	https://www2.mrc-lmb.cam.ac.uk/kzhang/
Gautomatch	N/A	https://www2.mrc-lmb.cam.ac.uk/kzhang/
RELION 3.0/3.1	Zivanov et al., 2018	https://www3.mrc-lmb.cam.ac.uk/relion/
AlphaFold	Tunyasuvunakool et al., 2021	https://alphafold.ebi.ac.uk/
Buccaneer	Hoh et al., 2020	https://phenix-online.org/
Phenix.real_space_refine	Afonine et al., 2018a	https://phenix-online.org/
Phenix.mtriage	Afonine et al., 2018b	https://phenix-online.org/
Coot	Emsley et al., 2010	https://www2.mrc-lmb.cam.ac.uk/personal/pemsley/coot/
PyMOL2	N/A	https://pymol.org/2/
UCSF Chimera	Pettersen et al., 2004	https://www.cgl.ucsf.edu/chimera/
UCSF ChimeraX	Goddard et al., 2018	https://www.cgl.ucsf.edu/chimerax/
ImageJ	Schneider et al., 2012	https://imagej.nih.gov/ij/
Tidyverse	N/A	https://www.tidyverse.org/
Illustrator	Adobe	https://www.adobe.com
Prism9	GraphPad	https://www.graphpad.com
Other		
HisTrap HP 5ml	Cytiva	17524805
Mono Q 5/50 GL	Cytiva	17516601
Superose 6 Increase 10/300 GL	Cytiva	29091596
StrepTrap HP 5 mL	Cytiva	28907548
Mono S 5/50 GL	Cytiva	17516801
Superdex 200 Increase 10/300 GL	Cytiva	28990944
Superose 6 Increase 3.2/300	Cytiva	29091598
Amicon Ultra MWCO100	Merck	UFC9100
Quantifoil R2/1 Cu 200 mesh	Quantifoil	Q210CR1
Quantifoil R2/1 Cu 200 mesh carbon supported	Quantifoil	Q210CR1-2nm

RESOURCE AVAILABILITY

Lead contact

Further information and requests for resources and reagents should be directed to and will be fulfilled by the lead contact, Elena Conti (conti@biochem.mpg.de).

Materials availability

This study did not generate new unique reagents.

Data and code availability

- Cryo-EM density maps have been deposited in the Electron Microscopy Data Bank (EMDB) and the Protein Data Bank (PDB), respectively, under the accession numbers: EMD-13923 (apo human SKI complex in the closed state, PDB: 7QDR), EMD-13925 (apo human SKI complex in the open state, PDB: 7QDS), EMD-13927 (RNA-bound human SKI complex, PDB: 7QDY), EMD-13928 (80S-bound human SKI complex in the closed state, PDB: 7QDZ) and EMD-13929 (80S-bound human SKI complex in the open state, PDB: 7QE0). Data are available at time of publication.
- Unprocessed and uncompressed imaging data is available at Mendeley Data: https://doi.org/10.17632/9w78m35s2v.1.
- This paper does not report original code.
- Any additional information required to reanalyse the data reported in this work/paper is available from the lead contact upon request.

EXPERIMENTAL MODEL AND SUBJECT DETAILS

All bacterial and eukaryotic cell lines in this study were used for protein production for in vitro experiments, rather than being experimental models in the typical sense. They are listed in the key resources table.

Recombinant proteins were either cloned or synthesized as described in the method details. *Spodoptera frugiperda 21* (*Sf21*) cells were maintained in Sf-900 II SFM medium (Thermo Fisher Scientific) at 27°C. *Escherichia coli* expression strains BL21 STAR pRARE (Thermo Fisher Scientific) were grown in TB medium at 37°C under antibiotic selection to an OD_{600nm} = 2 before inducing protein expression by adding 500 μM IPTG for 16 h at 18°C. HEK 293T cells were adapted to grow in suspension and stable transfected using the piggyBac transposon system (Li et al., 2013). Cells were maintained in FreeStyle 293 Expression medium (Thermo Fisher Scientific) at 37°C and 5% CO_2 and protein expression induced by adding 1 μg/mL doxycycline.

METHOD DETAILS

Cloning, protein expression and purification

The complete open reading frames of *SKIV2L*, *TTC37*, and *WDR61* (UniProt: Q6PGP7, Q15477, Q9GZS3) were cloned from a human cDNA library (MegaMan Human Transcriptome Library, Agilent Technologies) into separate expression cassettes on a single pACEBac1 vector (Bieniossek et al., 2012). A 3C protease cleavable 10xHis-tag on the *TTC37* N-terminus for immobilized metal affinity chromatography (IMAC) was added to all human SKI complex constructs (wild-type, E424Q, V341G, Δwedge, Δarch). The vector was integrated into an engineered baculovirus genome (Bieniossek et al., 2012) and cultured in *Sf21* cells for virus production. *Sf21* cells suspended at 10^6 cells/mL were infected with virus and cultured in Sf-900 II SFM medium at 27°C. Cultures were harvested after 72 h by centrifugation at 2000 g. Cell pellets were resuspended in lysis buffer containing 20 mM Hepes pH7.5, 200 mM NaCl, 25 mM Imidazole and 2mM ß-mercaptoethanol (ß-ME), and supplemented with 200 U/mL benzonase (Merck), 500 μM AEBSF protease inhibitor, and cOmplete EDTA-free protease inhibitor cocktail (Roche). The cells were lysed by ultrasonication (Bandelin, Sonopuls basic). The lysate was cleared by centrifugation at 75,000 g and subjected to a HisTrap HP 5ml column (GE Healthcare) for IMAC. The column was washed with 15 column volumes of 20 mM Hepes pH7.5, 1000 mM NaCl, 200 mM KCl, 10 mM $MgCl_2$, 25 mM Imidazole and 2 mM ß-ME, followed by washing with 5 column volumes of lysis buffer. Protein was eluted in lysis buffer supplemented with 350 mM Imidazole. The eluate was treated with 10 μg/mL His-tagged 3C protease (MPI Biochemistry Core Facility) during overnight dialysis against 20 mM Hepes pH7.5, 100 mM NaCl, 25 mM Imidazole and 2mM ß-ME. 3C protease and His-tags were removed by running the eluate over IMAC. The unbound protein fraction was further purified by ion exchange (Mono Q 5/50 GL, GE Healthcare) and size exclusion chromatography (Superose 6 Increase 10/300 GL, GE Healthcare), and eluted in a final buffer containing 20 mM Hepes pH7.5, 100 mM NaCl, 2 mM Dithiothreitol (DTT).

The complete open reading frame of the HBS1L3 (UniProt: Q9Y450) was commercially synthesized (Eurofins Genomics) and cloned with N-terminal 6xHis-thioredoxin and C-terminal eGFP-TwinStrep tags under a IPTG-inducible promotor for expression in *E. coli*. Transformed *E. coli* BL21 STAR pRARE cells were grown in TB medium at 37°C under antibiotic selection to OD_{600nm} = 2. The temperature was reduced to 18°C and protein expression was induced by addition of 500 μM IPTG. Cells were harvested after 16 h by centrifugation at 8,500 g. Cells were ultrasonicated in the same lysis buffer as above, except it contained 300 mM NaCl. The recombinant protein was kept strictly at 4°C and the purification procedure was processed quickly to avoid degradation. The lysate was cleared by centrifugation and loaded on a HisTrap HP 5 mL column (GE Healthcare) for IMAC. Washing was performed as above, and the protein was eluted and equilibrated with lysis buffer supplemented with 350 mM Imidazole into a StrepTrap HP 5 mL column (GE Healthcare) for a second affinity step. The bound protein was washed with 10 column volumes of buffer containing 20 mM Hepes pH7.5, 300 mM NaCl, 2 mM DTT and addition of 5 mM Desthiobiotin (DTB) eluted the protein. The protein was concentrated in 10 % Glycerol (v/v), flash frozen in liquid nitrogen (LN_2), and stored at -80°C.

The complete open reading frame human DIS3L (UniProt: Q8TF46) with the inactivating D486N mutation ($DIS3L_{D486N}$) was tagged with TwinStrep at the N-terminus and expressed in a HEK 293T stable cell line adapted to grow in suspension. Briefly, HEK 293T cells at 10^6 cells/mL were transfected in FreeStyle 293 Expression Medium (Thermo Fisher Scientific) with hDIS3L cloned into the piggyBac vector with a doxycycline-dependant inducible promoter (Li et al., 2013), the piggyBac transactivator and the hyperactive piggyBac transposase using polyethylenimine (PEI). After a 24 h recovery period, cells were positively selected for 21 days using puromycin and geneticin. 400-800 mL of stably transfected cells (10^6 cells/mL) were induced with 1 μg/mL doxycycline for 48 h and harvested by centrifugation at 800 g. Cells were resuspended in buffer containing 20 mM Hepes pH7.5, 300 mM NaCl, and 2 mM DTT and lysed using a dounce homogeniser. After clearing the lysate by centrifugation, it was loaded on a StrepTrap HP 5 mL column (GE Healthcare) and washed with high salt and lysis buffer (similar to hSKI). The protein was eluted in buffer containing 20 mM Hepes pH7.5, 100 mM NaCl, 5 mM DTB, and 2 mM DTT.

Ribosome subunit purification

Ribosomal 40S and 60S subunits were obtained from untransfected HEK 293T cells (using adapted protocols from Fernández et al. (2014) and Pisarev et al. (2007). 600 x 10^6 cells were pelleted and resuspended on ice for 30 min in lysis buffer containing 20 mM Hepes pH7.5, 300 mM NaCl, 6 mM $MgCl_2$, 0.5 % NP40 (v/v), 2 mM DTT, 500 μM AEBSF. The lysate was cleared by centrifugation at 10,000 g for 15 min and loaded on a 30% sucrose cushion in buffer containing 20 mM Hepes pH7.5, 150 mM KCl, 10 mM $MgCl_2$, 2 mM DTT, followed by ultracentrifugation in a TLA-100.3 rotor (Beckman Coulter) at 86,000 RPM for 90 min. The ribosomal pellets were resuspended in buffer containing 20 mM Hepes pH7.5, 75 mM KCl, 5 mM $MgCl_2$, 2 mM DTT, supplemented with 2 mM puromycin and incubated at 37°C for 15 min. The ribosome solution was then adjusted to 500 mM KCl and subjected to gradient centrifugation in a SW-40 Ti rotor (Beckman Coulter). The sucrose gradients (10-30% w/v) used for centrifugation were diluted in buffer containing 20 mM Hepes pH7.5, 500 mM KCl, 4 mM $MgCl_2$, and 2 mM DTT and mixed using a Gradient Station (Biocomp). After 17 h centrifugation at 22,8000 RPM, the gradients were fractionated from the top (Gradient Station, Biocomp). Fractions corresponding to 40S and 60S ribosomal subunits were pooled separately and concentrated in buffer containing 20 mM Hepes pH7.5, 50 mM KCl, 4 mM $MgCl_2$, and 2 mM DTT.

In vitro transcription of RNA substrates

The sequence of the CrPV IRES construct was taken from the intergenic region of the Cricket paralysis virus genome (NCBI GeneBank: 6025-6232 nt, NC_003924.1). The CrPV IRES was transcribed *in vitro* with T7 RNA polymerase by run-off transcription. The genomic sequence of the CrPV IRES was fused to a portion of the firefly luciferase open reading frame (modified from Petrov et al. [2016]). The CrPV-IRES29 construct was amplified by PCR and then gel purified. *In vitro* transcription of 100 nM CrPV IRES DNA template was carried out in 40 mM Tris-HCl pH8.0, 28 mM $MgCl_2$, 0.01% Triton X-100, 1 mM Spermidine, 5 mM DTT in the presence of 25 mM of each ribonucleotide (ATP, CTP, GTP, UTP, Jena Bioscience) and 100 U/μL T7 RNA Polymerase (MPI Biochemistry Core Facility) at 37°C for 4 h. The DNA template was digested with 1 U/μL DNase I (Roche) and purified by $LiCl_2$ precipitation.

The radioactive body-labelled GGGC(*UC)$_{48}$ RNA was transcribed similarly using an annealed duplex of two DNA oligos (5'-AATT TAATACGACTCACTATAGG-3' and 5'-TTAAATTATGCTGAGTGATATCCCGAGAGAGAGAGAGAGAGAGAGAGAGA GAGAGA GAGAGAGAGAGAGAGAGAGAGAGAGAGAGAGAGAGAGAGAGAGAGAGAGAGAGAGAGAG-3') as a template. Transcription of 0.1 μM template DNA was carried out in the same buffer containing nucleotides as above but with the addition of 1.5 μM radioactive [α^{32}P] UTP (PerkinElmer). After 4 h at 37°C and DNase I treatment, the reaction was phenol extracted, ethanol precipitated and gel purified from an 8% 7M Urea PAGE. The incorporation rate of [α^{32}P] UTP and final transcript concentration were quantified using a liquid scintillation counter (Hidex 300 SL).

ATPase activity assays

ATPase activity of human SKI complex and derivates were measured using a pyruvate kinase/lactate dehydrogenase enzyme-coupled assay (Bernstein et al., 2010). Reactions were prepared as serial two-fold dilutions of 2.5 mM ATP in buffer containing 50 mM Hepes pH7.5, 50 mM KAc, 5 mM $MgAc_2$, 2 mM DTT and 10 μg/mL poly(U) RNA (Sigma). After 20 min equilibration at 37°C, the reactions were started by adding 50 nM hSKI complex. NADH oxidation was monitored at 1 min intervals over the time course of 20 min by measuring absorbance at 340 nm using an infinite M1000PRO 96-well Plate reader (Tecan). All measurements were done in triplicates. A baseline oxidation level of NADH was established by measuring duplicate reactions without the addition of protein (see below for quantification and statistical analysis).

Cytoplasmic exosome reconstitution

Human cytoplasmic exosome ($hEXO10_c$ with the inactivated $hDIS3L_{D486N}$ nuclease) was reconstituted from equal molecular amounts of pre-assembled hEXO9 (purified as previously described) and freshly purified $hDIS3L_{D486N}$. After incubation for 30 min on ice, the salt concentration was adjusted to 50 mM NaCl. The reconstituted $hEXO10_c$ wild-type and mutant complexes were subjected to ion-exchange chromatography (Mono S 5/50 GL, GE Healthcare) and size exclusion chromatography (Superdex 200 Increase 10/300 GL, GE Healthcare) in a final buffer containing 20 mM Hepes pH7.5, 100 mM NaCl, and 2 mM DTT.

Size exclusion chromatography assays

Size exclusion chromatography studies were carried out sequentially over the course of one day using a Superose 6 Increase 3.2/300 (GE Healthcare) column and a buffer containing 20 mM Hepes pH7.5, 150 mM NaCl, and 2 mM DTT. The samples were prepared by mixing substoichiometric amounts of HBS1L3 with either 100 pmol hSKI (blue chromatogram in Figure 7A), or 100 pmol hEXO10$_c$ (red chromatogram in Figure 7A), or 100 pmol hSKI and 100 pmol hEXO10$_c$ (purple chromatogram in Figure 7A). In order to show that HBS1L3 is required to bridge the interaction between hSKI and hEXO10$_c$ and to from a higher order complex, 100 pmol hSKI with 100 pmol hEXO10$_c$ were mixed in absence of HBS1L3 (green chromatogram in Figure 7A). The samples were adjusted to 35 µl with buffer and incubated at 4°C for 15 min before injecting them onto the column. The absorbance at 280 nm was recorded in the chromatograms. For comparison of the retention volumes of the different complexes, the data was normalised and plotted in R using the tidyverse collection of R packages. Peak fractions of the individual gel filtration runs were TCA precipitated and analysed by SDS-PAGE on a NuPAGE 4-12% Bis-Tris gel (Thermo Fisher Scientific).

RNase protection assays

The cytoplasmic exosome complexes (Figure S7A) were concentrated at 500 nM and incubated with 125 nM radioactive body-labelled GGGC(*UC)$_{48}$ substrate in buffer containing 50 mM Hepes pH7.5, 50 mM NaCl, 5 mM $MgCl_2$, 10% Glycerol, 0.1% NP40, 2 mM DTT, and 1 mM ADP-BeF (premixed 1 mM ADP with 1 mM $BeCl_2$ and 5 mM NaF). After 60 min at 4°C to allow for the formation of the ribonucleoprotein complexes, the samples were RNase treated with 37.5 U/µL nM benzonase (Merck) at 25°C for 20 min. The reaction was stopped by adding 10x excess buffer containing 100 mM Tris-HCl pH7.5, 150 mM NaCl, 300 mM NaAc pH.5.2, 10 mM EDTA, and 1% SDS. The RNase protected RNA fragments were purified by phenol extraction and ethanol precipitation, separated on a denaturing 12% polyacrylamide gel containing 7M Urea, and analysed by phosphorimaging (Typhoon FLA7000, Cytiva).

Cryo-EM sample preparation

The substrate-free human SKI complex sample was prepared by concentrating 600 pmol of purified hSKI in buffer containing 20 mM Hepes pH7.5, 100 mM NaCl, and 2 mM DTT to approximately 30 µl. The sample was then crosslinked with 1.5 mM bissulfosuccinimidyl suberate (BS_3) at RT for 20 min and quenched with 5 mM Tris-HCl pH7.5. After centrifugation at 18,000 RCF for 10 min to pellet larger aggregates, the sample was injected onto a Superose 6 Increase 3.2/300 column (GE Healthcare) for size exclusion chromatography by an Aekta micro (GE Healthcare). A single peak fraction (containing approximately 500 nM) was collected and mixed with 0.04 % (v/v) n-octyl-ß-D-glucoside. 4-5 µl of the sample were applied to holey carbon grids (R2/1, 200 mesh, Quantifoil) and glow discharged with negative polarity at 20 mA for 30 sec using an EMS GloQube (MiTeGen). The sample was plunge frozen in a liquid ethane/propane mix using a Vitrobot Mark IV (Thermo Fisher Scientific) operated at 4°C and 95% humidity. Although we only show data from the samples prepared as above, we also prepared the substrate-free human SKI complex without addition of BS3 crosslinking and found similar distributions of closed and open state particles.

Substrate-bound human SKI was prepared in a similar manner as the substrate-free complex above with a few exceptions. Comparable amounts of purified hSKI were concentrated in buffer containing 20 mM Hepes pH7.5, 100 mM NaCl, 2 mM $MgCl_2$, 2 mM DTT, and supplemented with 1.5x molar excess of a 25-uracil RNA (Sigma) and 2 mM ADP-BeF. After incubation at 37°C for 15 min, the reconstituted complex was subjected to size exclusion chromatography and a single peak fraction was processed further without any BS3 crosslinking as described above.

The samples containing the 80S ribosome bound to human SKI complexes were prepared as follows: 100 pmol of purified human 40S was mixed with 150 pmol CrPV IRES carrying a 29 nt 3' overhang in buffer containing 20 mM Hepes pH7.5, 50 mM KCl, 4 mM $MgCl_2$, and 2 mM DTT. After incubation at 37°C for 5 min, 120 pmol human 60S was added and incubation proceeded for another 10 min. 200 pmol of purified substrate-free human SKI complex was added to the 80S-CrPV-IRES-29nt complex. The total volume was adjusted to 200 µl with the above buffer and incubated at 37°C for another 15 min before the sample was placed at 4°C for further treatment. The samples were either incubated with 2 mM ADP-BeF or 1 mM ATP at 37°C for 15 min. ATP treatment was followed by addition of 2 mM ADP-BeF for another 15 min at 37°C to block the ATPase activity of hSKI. The samples were subjected to gradient centrifugation in a SW-40 Ti rotor (Beckman Coulter) at 4°C. 15-40% sucrose gradients (w/v) in buffer containing 20 mM Hepes pH7.5, 50 mM KCl, 4 mM $MgCl_2$, and 2 mM DTT were mixed using the Gradient Master (Biocomb). After 17 h centrifugation at 22,800 RPM, the gradients were fractionated using a Piston Gradient Fractionator (Biocomb). Fractions corresponding to the 80S-CrPV-IRES-SKI complex were concentrated in buffer containing 20 mM Hepes pH7.5, 50 mM KCl, 4 mM $MgCl_2$, and 2 mM DTT by centrifugation at 3,000 RCF using an Amicon Ultra MWCO100 centricon (Millipore). The concentrated samples at an OD_{260} of approximately 8 was supplemented with 0.04 % (v/v) n-octyl-ß-D-glucoside and subsequently used for plunge freezing. The sample was incubated twice for 2 min on carbon supported grids (R2/1, 200 mesh, Quantifoil), previously glow discharged with negative polarity at 20 mA for 20 sec using an EMS GloQube (MiTeGen). Plunge freezing was otherwise carried out as for the substrate-free sample above.

Cryo-EM data collection and processing

The cryo-EM data from substrate-free human SKI were collected on a FEI Titan Krios microscope (Thermo Fisher Scientific) at 300 kV equipped with a Gatan K3 direct electron detector operating in electron counting mode. The microscope is equipped with a post column energy filter set to slit-width of 20 eV. Images were collected by under-focused acquisition (target range

CellPress
OPEN ACCESS

of -0.6 and -2.4 μm) at a nominal magnification of 81,000x set up in SerialEM (Schorb et al., 2019) utilising a beam-tilt based multi-shot acquisition scheme for faster imaging. This resulted in 11,079 micrograph movies (40 movie frames each) acquired at a pixel size of 1.094 Å/pixel with a total exposure of 47.44 e/Å^2 over 4 sec. The collected data were processed in RELION 3.0 and 3.1 (Zivanov et al., 2018). To correct for beam-induced motions and minimize effects of radiation damage, the raw movie frames were aligned using MotionCor2 (Zheng et al., 2017). The aligned micrographs were used to estimate per-micrograph contrast transfer function (CTF) parameters with GCTF (Zhang, 2016). Initial particles (4,400,241) were selected using Gautomatch (http://www.mrc-lmb.cam.ac.uk/kzhang/). Four times down-sampled particles were extracted from the aligned, exposure-weighted micrographs. Reference-free 2D classification and classification in 3D using a 40 Å low-pass filtered starting model based on the crystal structure of the yeast SKI complex (PDB: 4BUJ) resulted in an intermediate subset of 350,147 well-aligning hSKI particles. 3D classification of this particle subset, extracted at the original pixel size, into six classes gave three reconstructions of human SKI complex in the closed state (global resolutions ranging from 8.4 to 10.6 Å), one reconstruction in the open state (9.2 Å), and two at lower resolution. A 40 Å low-pass filtered reconstruction of the closed state complex served as a starting model for the classification. Accuracy and resolution of the reconstructions could be improved by extending to 40 iterations of classification. However, it proved difficult to quantify an equilibrium between open and closed state particles from this classification, in part because a large portion of the particles, e.g. the gatekeeping module, adopts a very similar conformation in the two states. Therefore, we deliberately biased the classification of a particle subset after an initial round of 2D classification using 30 Å low-pass filtered open and closed state starting models (Figure S1). These classifications suggest a particle distribution of roughly 40% in closed and 60% in open state. The classification procedures resulted in two homogenous subsets of particles for open and closed state human SKI complex. Their reconstructions reached a global nominal resolution of 3.7 Å and 3.8 Å after masked 3D auto-refinement and automatic b-factor sharpening (-88.1 and -145.3) in the RELION post-processing routine (Zivanov et al., 2018) according to the Fourier shell correlation (FSC) cut-off criterion for the independent half maps of 0.143 (Rosenthal and Henderson, 2003). Peripheral regions of the reconstructions, particularly for the closed state, could be improved by focused refinements with local searches. Masks were applied to TPRs 34-40 of hSKI3$_C$, hSKI2$_{cat}$, hSKI2$_{arch}$ and the hSKI2$_N$ outer segments to improve local resolutions and volume connectivity.

Substrate-bound human SKI data were collected similar to the substrate-free complex above, but at 105,000x nominal magnification. A total of 11,413 micrograph movies were acquired at a pixel size 0.8512 Å/pixel with a total exposure of 68.31 e/Å^2 over 3 sec and spread over 30 movie frames. Beam-induced motion correction, CTF estimation, particle picking, and processing were done in a similar way as for the substrate-free data. 6,036,405 down-scaled particles were initially extracted from the aligned, exposure-weighted micrographs and particles that appeared to be non-hSKI discarded by 2D and 3D classification. A resulting subset of 534,613 human SKI particles were found and extracted at the original pixel size of 0.8512 Å/pixel. Further 3D classification into 6 classes, using a starting model based on these data, lead to the selection of a final subset of 144,441 particles. Initial 3D refinement estimated an overall nominal resolution of 3.5 Å. The quality of the reconstruction and the level of resolved detail was further improved by Bayesian polishing to correct for per-particle motion and by stepwise refinement of the per-particle CTF (taking into account the beam-tilted data acquisition scheme). This resulted in a final reconstruction with an overall global resolution estimated as 3.1 Å according to the gold-standard Fourier shell correlation (0.143) criterion. Masking and map sharpening using an automatically determined global b-factor of -52.3 was carried out in the RELION post-processing routine (Zivanov et al., 2018).

ADP-BeF-treated hSKI-ribosome data were measured on a FEI Titan Krios microscope at 105,000x nominal magnification. The K3 detector was used in correlated-double sampling mode (CDS) and the energy filter set to slit width of 10 eV. 15,073 micrographs movies were acquired with a total exposure of 55.8 e/Å ^{2} equally spread over 35 frames during 5 sec. On-the-fly micrograph movie processing was assisted by Focus (Biyani et al., 2017), which ran Motioncor2 (Zheng et al., 2017), GCTF (Zhang, 2016), and Gautomatch (https://www2.mrc-lmb.cam.ac.uk/research/locally-developed-software/zhang-software/#gauto) on individual images while the data were being collected. Subsequent particle processing was carried out in RELION 3.1 (Zivanov et al., 2018). Down-scaled particles were extracted from the aligned, exposure-weighted micrographs and classified in 2D and 3D to discard non-ribosomal and low-resolution particles from the data. The cleaned ribosomal particles (1,601,273) were extracted at the original pixel size of 0.8512 Å/pixel and aligned in a 3D auto-refinement with a spherical mask using a 40 Å down-filtered starting model based on PDB: 4UG0 (Khatter et al., 2015). The refined particles were then used to subtract a large portion of the 80S ribosome signal from the corresponding particle images. To improve alignment precision and the quality of the reconstructions in the subsequent steps, the particle images were then recentred on the remaining hSKI signal and re-extracted in a smaller box in RELION 3.1 (Zivanov et al., 2018). The subtracted particle images were 3D classified with local search into 7 classes using a wide mask. The final subset of consisted of 51,048 particles. The ribosome-signal-subtracted particles were aligned by 3D auto-refinement, which resulted in a reconstruction of hSKI in the closed state at an overall nominal resolution of 3.6 Å according to the FSC cut-off criterion of 0.143. For masking and map sharpening in the RELION 3.1 post-processing procedure (Zivanov et al., 2018), an ad-hoc b-factor of -20 was applied. The automatically determined b-factor of -122.6 resulted in an over-sharpened map with loss of connectivity. This discrepancy might be due to successive subtraction of a large portion of the total signal in the particle stacks. Next, the signal subtraction was reverted and the corresponding 80S-IRES-hSKI particles refined to 3.1 Å global resolution according to the FSC cut-off criterion of 0.143. A b-factor of -70.7 was automatically estimated in the RELION post-processing routine (Zivanov et al., 2018). The quality of the map in areas of the

CrPV IRES and hSKI complex, however, was not satisfactory. Therefore, we subtracted the signal of the 60S ribosomal subunit from the reconstruction and aligned the remaining 40S-IRES-hSKI particles by 3D auto-refinement, which yielded a reconstruction at 3.1 Å global resolution according to the FSC cut-off criterion of 0.143. Masking and map sharpening in RELION post-processing (Zivanov et al., 2018) was performed using an ad-hoc b-factor of -10 (automatically determined b-factor -73.6). While the map quality for hSKI in this reconstruction improved only marginally, the resolution and volume connectivity for the CrPV IRES in the intersubunit space improved significantly.

ATP-treated hSKI-ribosome data were collected on a FEI Titan Krios microscope at 105,000x nominal magnification. 21,212 micrograph movies were acquired with a total dose of 67.6 e/Å^2 equally spread over 40 movie frames during 6 sec exposure time. On-the-fly data processing was assisted by Focus (Biyani et al., 2017) as described above and continued in RELION 3.1 (Zivanov et al., 2018). 2D and 3D classification resulted in 1,089,263 clean ribosomal particles, which were aligned in 3D auto-refinement for subsequent subtraction of a large portion of the 80S ribosome signal. The subtracted particle images of the ATP-treated hSKI-ribosome data were 3D classified with local searches into 6 classes using a wide mask. The classification resulted in one class that shows well-aligning density for the hSKI complex. In a second round of 3D classification using a 30 Å low-pass filtered starting model of the open state, separation of open and closed particles was possible and resulted in selection of a final subset of 76,838 open state particles. 3D auto-refinement of the subset was exclusively possible with local searches and resulted in a reconstruction at 6.5 Å global resolution according to the FSC cut-off criterion of 0.143. A b-factor of -210.6 was estimated automatically using a tight mask in the RELION post-processing procedure (Zivanov et al., 2018). For the corresponding ribosome-bound reconstructions, signal reversion and subtraction procedures similar to the ADP-BeF-treated hSKI-ribosome data above were applied. This led to an open 80S-IRES-hSKI reconstruction at 3.0 Å global resolution using a -76.3 b-factor and an open 40S-IRES-hSKI reconstruction at 3.0 Å global resolution using an ad-hoc b-factor of -10 (automatically determined b-factor -79.2).

Data processing on both hSKI-ribosome data sets required signal subtraction of the ribosome to yield reconstructions of comparable interpretability and quality as those described in the manuscript. Classical focused classification and refinement procedures did not yield results of comparable quality and interpretability.

Density interpretation and model building

Resolution and quality of the substrate-free hSKI reconstruction in closed state enabled us to build its structure de novo. Structure building was guided by a Buccaneer initial model within the CCP-EM suite (Hoh et al., 2020) based on published high-resolution yeast Ski structures (Halbach et al., 2012, 2013). The structure was then further completed and refined in Coot (Emsley et al., 2010) and iteratively finalised using real-space refinement in the Phenix suite (Liebschner et al., 2019). Rigid-body fitting using UCSF Chimera (Pettersen et al., 2004) into focussed refined maps enabled us to build peripheral regions of the complex: TPRs 34-40 in the C-terminus of hSKI3$_C$; TPRs 10-14 in the N-terminal region of hSKI3$_C$ (α-helices without sequence); parts of the solvent exposed areas of hSKI2$_N$ (with register defined by AlphaFold prediction); the globular part of hSKI2$_{arch}$ (which was interpreted with an AlphaFold prediction) (Figure S2H**)**. The reconstruction of substrate-free hSKI in open state was interpreted by rigid-body fitting (UCSF Chimera) a hSKI2$_{cat/arch}$ depleted version of substrate-free hSKI structure in closed state followed by modulating and refinement of the open state hSKI structure in Coot (Emsley et al., 2010) and real-space refinement (Phenix). Both closed and open state substrate-free human SKI reconstructions were validated using MolProbity (Chen et al., 2010) within the cryo-EM validation tool in the phenix suite (Liebschner et al., 2019).

The substrate-bound hSKI reconstruction was interpreted by rigid-body fitting (UCSF Chimera) of the substrate-free hSKI structure in the closed state described above. The six ribonucleotides of the 25-uracil RNA substrate were built into the structure of substrate-bound hSKI using Coot, and then refined and finalised by real-space refinement (Phenix). The final substrate-bound human SKI complex reconstruction was validated using MolProbity (within the cryo-EM validation tool in the phenix suite).

The reconstruction of closed state hSKI bound to ribosome was interpreted by rigid-body fitting (UCSF Chimera) the structure of substrate-bound hSKI. Small variations in hSKI2$_{cat}$ and the six ribonucleotides within hSKI2$_{cat}$ were adjusted and refined using Coot and real-space refinement (Phenix). This model along with the structure of the 40S ribosomal subunit taken from PDB: 4UG0 (Khatter et al., 2015) and the structure of the CrPV IRES mimicking a pre-translocated ribosomal state (PDB: 4V92 [Fernández et al., 2014]) were all used to interpret the corresponding 40S-IRES-hSKI reconstruction in closed state by rigid-body fitting (UCSF Chimera). The corresponding 80S-IRES-hSKI reconstruction in closed state was subsequently interpreted by rigid-body fitting the above mentioned 40S-IRES-hSKI structure in closed state together with the structure of the 60S ribosomal subunit from the structure of the human 80S ribosome (PDB: 4UG0).

The reconstruction of open state hSKI bound to ribosome was interpreted by rigid-body fitting (UCSF Chimera) the structure of the helicase module from the structure of substrate-bound hSKI detailed above. The structure was further completed in Coot by building additional downstream ribonucleotides traversing hSKI2$_{cat}$. Small variations with respect to hSKI2$_{cat}$ and the ribonucleotides inside hSKI2$_{cat}$ were adjusted and refined in Coot and real-space refinement. The corresponding 40S-IRES-hSKI reconstruction in open state was interpreted similar to the closed state above by rigid-body fitting the ribosome-bound structure of hSKI in open state and the structures of human 40S ribosomal subunit (PDB: 4UG0) and CrPV IRES (PDB: 4V92). The PK-1 nucleotides of the CrPV IRES in the intersubunit space of the ribosome were omitted from the structure where there was no apparent density. The corresponding full 80S-IRES-hSKI reconstruction in open state was interpreted similarly as the one in the closed state above by rigid-body

fitting the 40S-IRES-hSKI structure in the open state together with the structure of the 60S ribosomal subunit (PDB: 4UG0). In the closed and open state 40S-IRES-hSKI reconstructions the same volume level was applied to compare the PK-1 CrPV IRES densities in the intersubunit space without bias.

QUANTIFICATION AND STATISTICAL ANALYSIS

The kinetic parameters of ATP hydrolysis by human SKI were calculated according to Michaelis-Menten theory at various ATP substrate concentrations under steady-state conditions. The baseline corrected initial velocities (v_0) as a function of substrate concentration were used to approximate the Michaelis-Menten equation ($v_0 = (v_{max} \cdot [S]) / (K_m + [S])$). The kinetic parameters v_{max}, K_m and k_{cat} were derived from the approximation using a total enzyme concentration $[E_{tot}] = 0.05$ μM. The approximation of the Michaelis-Menten equation was done using a non-linear regression model in the Prism9 (GraphPad) software.

Molecular Cell, Volume *82*

Supplemental information

The human SKI complex regulates channeling of ribosome-bound RNA to the exosome via an intrinsic gatekeeping mechanism

Alexander Kögel, Achim Keidel, Fabien Bonneau, Ingmar B. Schäfer, and Elena Conti

Supplementary Figure S1. Cryo-EM data analysis of recombinant apo hSKI (related to Figures 1, 2 and 3)

Characteristics of the apo hSKI single particle cryo-EM sample and data. **(A) On the left,** analytical size exclusion chromatography profile on a S6 increase column (Cytiva) of a representative hSKI preparation, and on the right is a 4-12 % Coomassie stained SDS-PAGE of a peak fraction of the SEC run (labelled with *, M is Marker). In **(B)** a representative cryo-EM micrograph of the apo hSKI sample (A) is shown. This image was recorded at 300 kV with a pixel size of 1.024 Å/pix using a post-GIF K3 direct detector. **(C)** 2D class averages of the apo hSKI sample in the closed (left hand panel, red frame) and open (right hand panel, blue frame) state conformations. **(D)** Processing scheme of the single particle cryo-EM dataset of the apo hSKI sample resulting in 3D reconstructions for the closed (red frame) and open (blue frame) states.

Supplementary Figure S2. Cryo-EM reconstruction analysis of apo hSKI (related to Figures 1, 2 and 3)

Quality indicators of the apo hSKI closed and open 3D reconstructions. Local resolution estimates and Fourier Shell Correlations (FSC) of the closed **((A)** and **(B))** and the open **((D)** and **(E))** reconstructions of the apo hSKI cryo-EM SPA data. The FSC of the masked independent half maps were calculated in the RELION 3.1 postprocessing routine and the map vs model FSC using phenix.mtriage. The FSC cut-off criteria of 0.5 and 0.143 are indicated by dotted lines. Angular sampling distributions of the closed **(C)** and open **(F)** reconstructions of the apo hSKI data. Sampling angle data were plotted in 3º by 3º bins and sampling bins coloured according to particle number with red indicating more and blue fewer particles. **(G)** Selected model and map regions of the closed state reconstructions showing the quality of the structural data. **(H)** Local resolution estimate of a focused 3D reconstruction of the $hSKI2_{arch}$ (panel on the left) used for rigid fitting of a homology model of this domain (panel on the right) and completion of the closed state model.

Supplementary Figure S3. Structural conservation between human SKI and yeast Ski (related to Figures 2, 3, 5 and 6)

Comparisons of the hSKI apo and the *S. cerevisiae* Ski structures. In **(A)** the model of hSKI closed apo state is juxtaposed to the yeast Ski model. SKI2 is coloured in yellow,

SKI3 in blue, $SKI8_{IN}$ in green and $SKI8_{OUT}$ in dark green. The SKI3 C-terminal module is absent in the yeast structure (box (B)). **(B)** Detailed view of the C-terminal module of hSKI3 in complex with the N-terminus of the hSKI2. **(C)** The hSKI2 N-terminal outer a-helix is sandwiched between hSKI3 TPR13 and TPR14. Note that the residue numbering of the $Ski2_N$ a-helix (see also Figure 2C) is based on prediction from AlphaFold (Tunyasuvunakool et al., 2021). A focused 3D reconstruction on the first superhelical turn of hSKI3 in the hSKI apo closed state is coloured according to local resolution on the left and displayed together with the respective part of the model on the right. **(D)** hSKI2 helicase modules of the apo hSKI closed state, the RNA-bound hSKI closed state and the *S.c.* Ski structure in comparison. Note the similar orientation and conformation of the $SKI2_{arch}$ with respect to the $SKI2_{cat}$ in all three structures. **(E)** Comparison of the human and the yeast 80S bound by human and yeast SKI complex respectively (*S. cerevisiae* 80S-Ski complex from PDB:5MC6). SKI2 is coloured in yellow, SKI3 in blue, $SKI8_{IN}$ in green, $SKI8_{OUT}$ in dark green, the CrPV IRES in the human case and the mRNA and tRNA in the yeast case in red, the 60S ribosomal subunit is in light blue and the 40S ribosomal subunit in light orange. The yeast and human SKI complexes sit in different orientations on the respective 80S and the RNAs appear differently bound to the helicase modules. In the yeast case this leaves 2-3 nucleotide positions to ySki2 R149 unoccupied (ySki2 R149 is structurally in the equivalent position as hSKI2 W146).

Supplementary Figure S4. SKI2 elements impacting the open/closed state (related to Figure 2, 3 and 4)

Details of selected elements in the $hSKI2_N$. In **(A)** a multiple sequence alignment (MSA) of eukaryotic SKI2 homologs focussed on the N-terminal wedge and a schematic representation of this region in the hSKI2 construct used for the hSKI-Δwedge complex is plotted. Degrees of conservation are indicated by shades of yellow in the MSA with darker shades highlighting stronger conservation. The organisation of the different motives of interest in proximity to the hSKI2 wedge is indicated in the cartoon atop the MSA by thicker yellow lines. A Coomassie stained SDS-PAGE of this sample is shown in Fig. 7B. These samples were vitrified on holey-carbon Cu grids and imaged at 200kV using a K2 direct detector in counting model at a pixel size of 1.885 Å/pix. In the top panel in **(B)** reference-free 2D class averages of these cryo-EM SPA data of the

hSKIΔwedge complex are displayed. These 2D class averages are overall similar to those of the apo open hSKI particles in Fig. S1C. The apo open hSKI complex was fitted into the low-resolution 3D reconstruction of the hSKIΔwedge complex in the panel at the bottom. The unmodelled density on the right with corkscrew-like appearance corresponds to the unmodelled hSKI3 N-terminal TPRs. Note the absence of any interpretable density for the hSKI2 helicase module. hSKI2 is coloured in yellow, hSKI3 in blue, $hSKI8_{IN}$ in green and $hSKI8_{OUT}$ in dark green. In single particle cryo-EM, a *S. cerevisiae* Ski-Δarch complex adopts an open and closed conformation. **(C)** Coomassie stained 4-12 % SDS-PAGE of the yeast SkiΔarch complex. These samples were vitrified on holey-carbon Cu grids and imaged at 200kV using a K2 direct detector operated as above. Reference-free 2D class averages and the final 3D reconstruction of the closed state of the yeast Ski-Δarch complex are in **(D)**. **(E)** shows reference-free 2D class averages and the final 3D reconstruction of the open state of the yeast Ski-Δarch complex. Ski2 is coloured in yellow, Ski3 in blue, $Ski8_{IN}$ in green and $Ski8_{OUT}$ in dark green. **(F)** Fourier shell correlation of the masked independent half-maps for the 3D reconstructions of hSKIΔwedge (shown in **(B)**, forest green) as well as the closed and open states of the yeast Ski-Δarch complex (shown in **(D)** and **(E),** coloured in salmon and olive, respectively)**.**

Supplementary Figure S5. Characteristics of the RNA-bound closed hSKI single particle cryo-EM data (related to Figure 4)

(A) shows a representative cryo-EM micrograph and reference-free 2D class averages of the RNA-bound closed hSKI sample recorded at 300 kV with a pixel size of 0.8512 Å/pix using a Gatan K3 camera. In **(B)** the processing scheme of the single particle cryo-EM dataset of the hSKI sample is outlined. This resulted in the final 3D reconstruction with a global resolution estimate of 3.1 Å for the closed RNA-bound state (red frame). The local resolution estimates are plotted on the final reconstruction in **(C)**, the FSC of the masked independent half-maps of the final reconstruction and the model vs map FSC in **(D)**. Here the FSC cut-off criteria of 0.5 and 0.143 are indicated by dotted lines. The angular sampling distribution for the final reconstruction is in **(E).** Angular sampling data were plotted in 3º by 3º bins in this panel. Note the absence of any noticeable fraction of open state particles in these SPA data (see 3D classification in **(B)**). Comparisons of the closed RNA-bound hSKI2 helicase with

hMTR4. In **(F)** RNA-bound human MTR4 in grey is superposed onto the closed RNA-bound hSKI2 helicase in yellow (human MTR4 from PDB:6D6Q). The RNA is coloured in red. Note the very similar path of the RNA in both structures. **(G)** Details of the RNA channel in the helicase module of the RNA-bound hSKI apo closed state similar to Fig. 4C. Residues interacting with the RNA are shown in stick representation. Colour scheme as in **(F)**. **(H)** Superposition of the RNA bound hSKI2 with human MTR4 bound to ZCCHC8$_{CTD}$. Residues W146 of the hSKI2$_{wedge}$ and F673 of the ZCCHC8$_{CTD}$ occupy very similar positions at one end of the RNA channel in the two structures (MTR4: ZCCHC8$_{CTD}$ from PDB:6C90). In the RNA-bound closed hSKI structures this residue closes off the RNA channel of the hSKI2 helicase at the 3' end prohibiting the RNA from further threading through.

Supplementary Figure S6. Characteristics of the closed ribosome-bound hSKI single particle cryo-EM sample and data (related to Figure 6)

(A) Typical 15-40 % (w/v) Sucrose gradient profile used for reconstitution of the closed state ADP-BeF-treated human 80S-IRES-hSKI complex on the left (80S-IRES-hSKI black curve; 80S-IRES grey curve). Peak fractions (indicated by *) were run out on a 4-12 % SDS-PAGE and the Coomassie stained gel is in the right panel. hSKI8 is not clearly identifiable on the gel most likely due to the prevalence of human 80S ribosomal proteins between 30 and 40 kDa. These peak fractions were vitrified and imaged at 300 kV using a post-GIF K3 camera with a pixel size of 0.8512 Å/pix **(B)**. Reference-free 2D class averages of the closed state 80S-IRES-hSKI sample are shown in **(C)**. Density for the hSKI complex is discernible atop the 40S ribosomal subunit in most of these 2D class averages. **(D)** displays the processing workflow for the closed ribosome-bound hSKI single particle cryo-EM data resulting, after subtraction of the 80S density, in a reconstruction of the hSKI complex at 3.6 Å resolution. The red masks indicate the regions of the map not subtracted from the data. The angular sampling distribution, the FSC of the independent masked half-maps and the map vs model FSC as well as the local resolution estimates plotted on the final closed ribosome-bound hSKI structure are shown in **(E)**, **(F)** and **(G)** respectively. In **(F)** dotted lines indicate the FSC cut-off criteria of 0.5 and 0.143.

Supplementary Figure S7. Characteristics of the open ribosome-bound hSKI single particle cryo-EM data (related to Figure 7)

(A) The purified protein samples used in the RNase protection assays in Figure 7B are shown on a Coomassie-stained 4-12 % SDS-PAGE. The samples are peak fractions from size-exclusion chromatography purifications. In **(B)** a representative micrograph (at a pixel size of 0.8512 Å/pix) and in **(C)** reference-free 2D class averages of the ATP-treated 80S-IRES-hSKI complex in open state are shown. In most 2D class averages density for the hSKI2 helicase at the top of the 40S ribosomal subunit is identifiable. Note the distinct structure of the $hSKI2_{arch}$ in these 2D class averages. **(D)** shows the local resolution estimates for the open ribosome bound hSKI structure from which the density of the 80S has been subtracted. In **(E)** angular sampling distribution for the reconstruction in **(D)** is plotted and in **(F)** the FSC for the masked independent half-maps and the model vs map for said reconstruction with dotted lines indicate the FSC cut-off criteria of 0.5 and 0.143. **(G)** shows the processing scheme with the red masks indicating the region of the map not subtracted from the data. After subtraction both 3D classifications with either apo closed hSKI or open ribosome-bound hSKI as reference model identify sub-populations of both the open and the closed hSKI state in these data. Of the ~110 000 particles identified initially as hSKI-bound, approximately 65% appear clearly in the open state in the second round of focused 3D classification using the open state as start model. The hSKI2 helicase is well ordered in these data since the 80S serves as an anchor for its substrate engagement. This is in contrast and somewhat paradoxical to the apo open hSKI state, where the helicase domain appears flexible in relation to the rest of the complex.

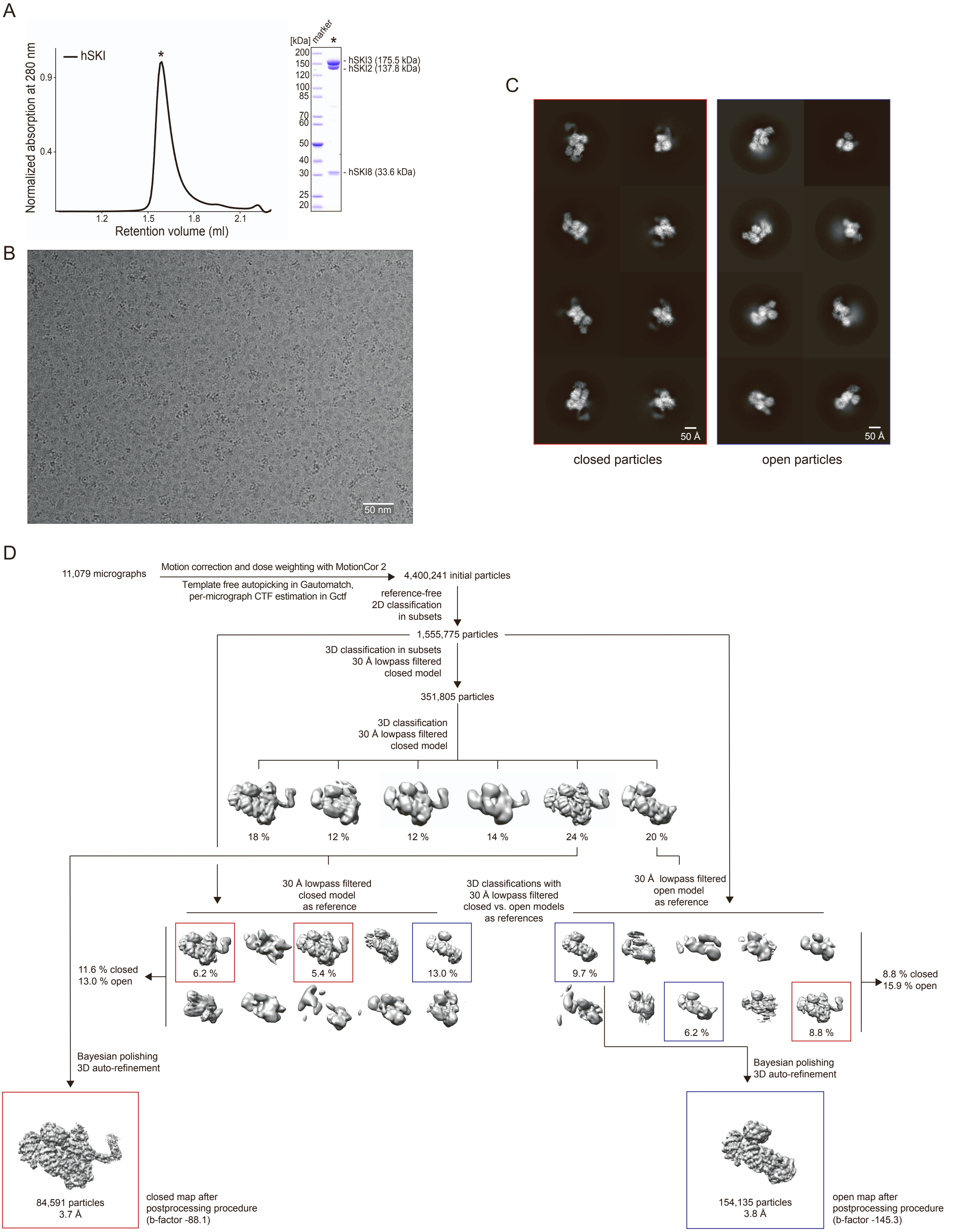
A
hSKI
Normalized absorption at 280 nm
Retention volume (ml)
[kDa] marker
hSKI3 (175.5 kDa)
hSKI2 (137.8 kDa)
hSKI8 (33.6 kDa)
B
50 nm
C
50 Å
closed particles
open particles
D
11,079 micrographs
Motion correction and dose weighting with MotionCor 2
Template free autopicking in Gautomatch, per-micrograph CTF estimation in Gctf
4,400,241 initial particles
reference-free 2D classification in subsets
1,555,775 particles
3D classification in subsets 30 Å lowpass filtered closed model
351,805 particles
3D classification 30 Å lowpass filtered closed model
18 %
12 %
12 %
14 %
24 %
20 %
30 Å lowpass filtered closed model as reference
3D classifications with 30 Å lowpass filtered closed vs. open models as references
30 Å lowpass filtered open model as reference
6.2 %
5.4 %
13.0 %
9.7 %
6.2 %
8.8 %
11.6 % closed
13.0 % open
8.8 % closed
15.9 % open
Bayesian polishing
3D auto-refinement
84,591 particles
3.7 Å
closed map after postprocessing procedure (b-factor -88.1)
154,135 particles
3.8 Å
open map after postprocessing procedure (b-factor -145.3)

Figure S1

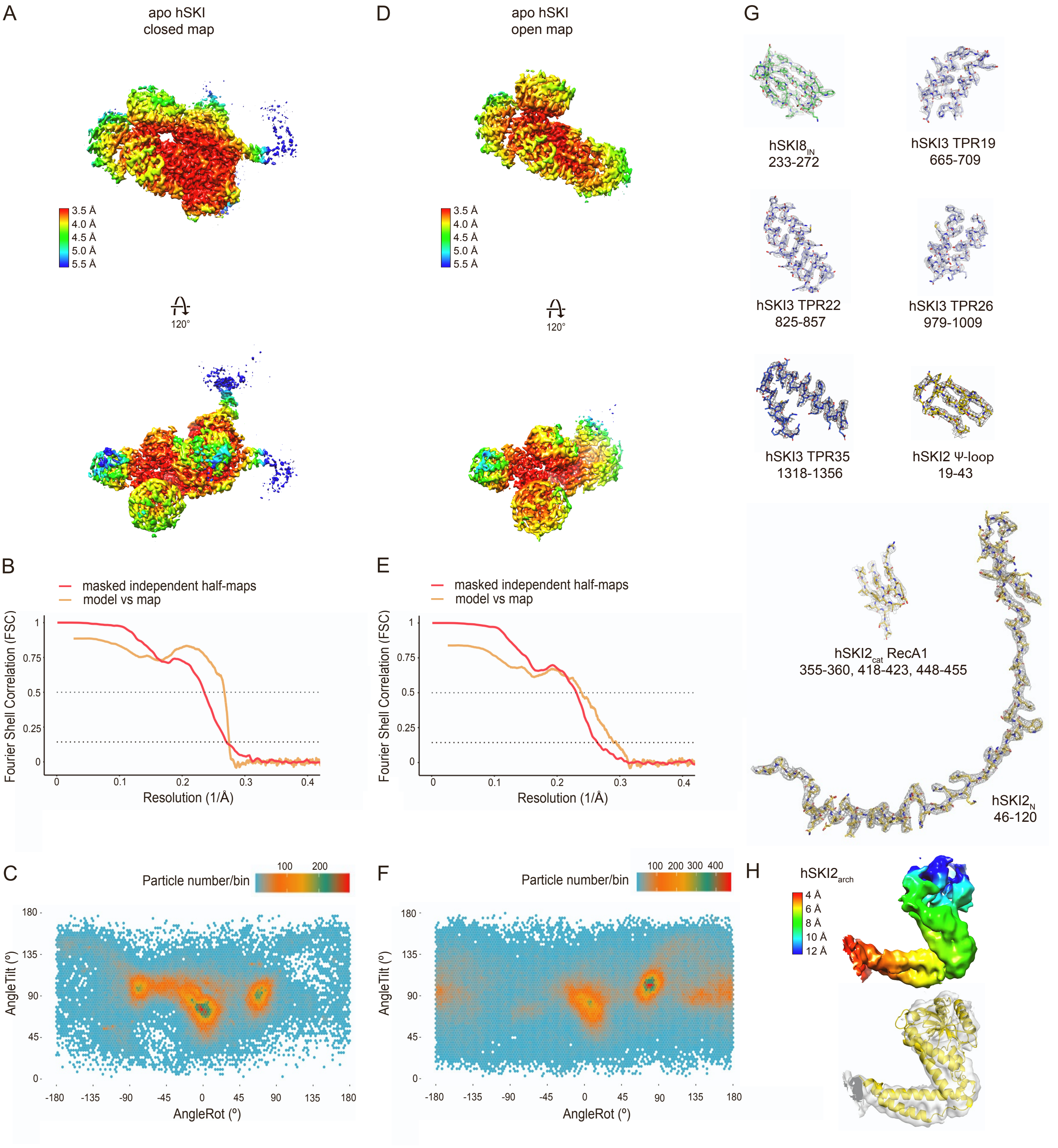

A
apo hSKI
closed map
3.5 Å
4.0 Å
4.5 Å
5.0 Å
5.5 Å
120°
D
apo hSKI
open map
3.5 Å
4.0 Å
4.5 Å
5.0 Å
5.5 Å
120°
G
hSKI8IN
233-272
hSKI3 TPR19
665-709
hSKI3 TPR22
825-857
hSKI3 TPR26
979-1009
hSKI3 TPR35
1318-1356
hSKI2 Ψ-loop
19-43
hSKI2cat RecA1
355-360, 418-423, 448-455
hSKI2N
46-120
B
masked independent half-maps
model vs map
Fourier Shell Correlation (FSC)
Resolution (1/Å)
E
masked independent half-maps
model vs map
Fourier Shell Correlation (FSC)
Resolution (1/Å)
C
Particle number/bin
100 200
AngleTilt (°)
AngleRot (°)
F
Particle number/bin
100 200 300 400
AngleTilt (°)
AngleRot (°)
H
hSKI2arch
4 Å
6 Å
8 Å
10 Å
12 Å

Figure S2

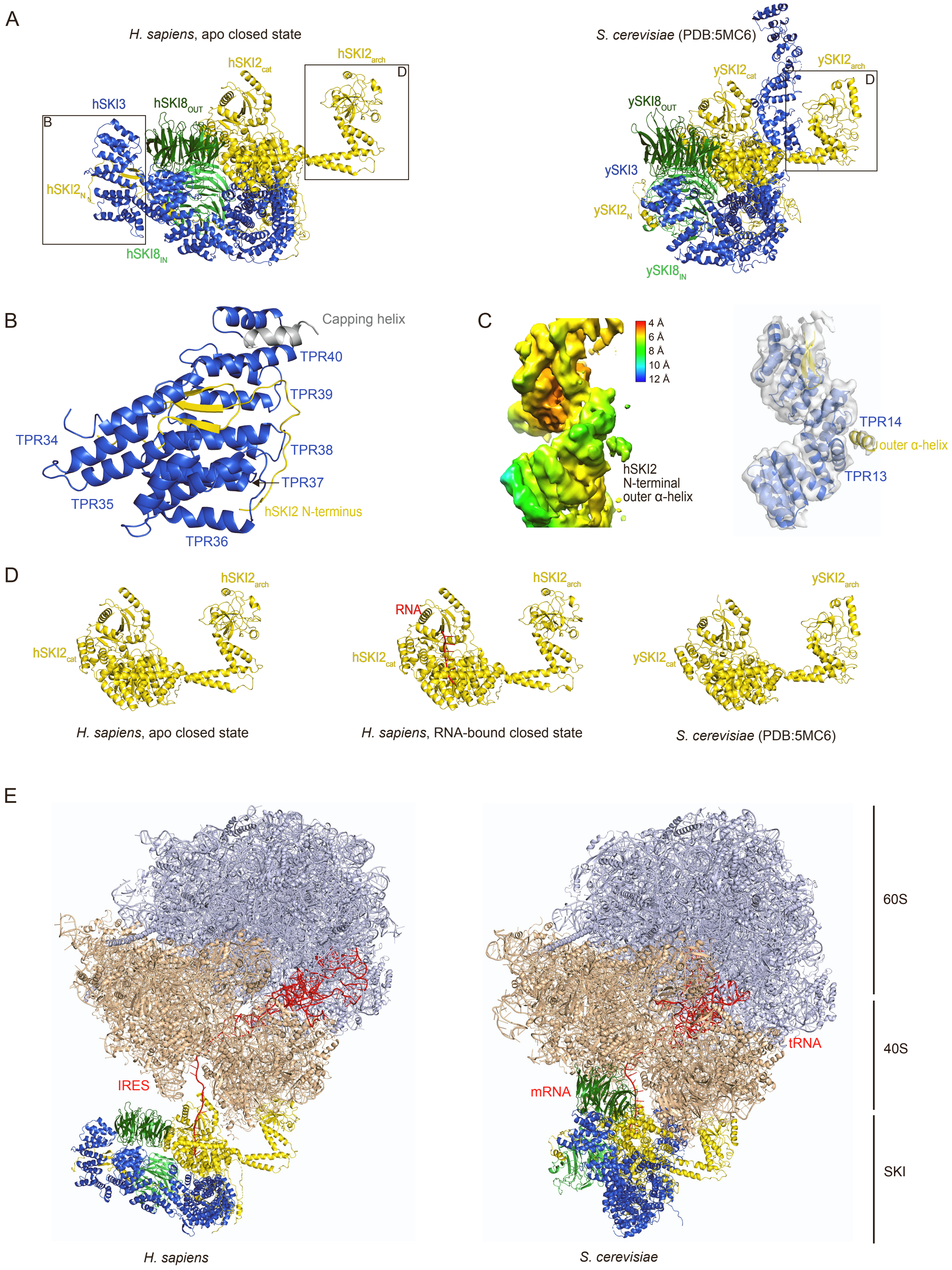

A
H. sapiens, apo closed state
hSKI2cat
hSKI2arch
hSKI3
hSKI8OUT
hSKI2N
hSKI8IN
B
D
S. cerevisiae (PDB:5MC6)
ySKI2cat
ySKI2arch
ySKI8OUT
ySKI3
ySKI2N
ySKI8IN
B
Capping helix
TPR40
TPR39
TPR34
TPR38
TPR37
TPR35
hSKI2 N-terminus
TPR36
C
4 Å
6 Å
8 Å
10 Å
12 Å
hSKI2
N-terminal
outer α-helix
TPR14
outer α-helix
TPR13
D
hSKI2arch
hSKI2cat
H. sapiens, apo closed state
RNA
H. sapiens, RNA-bound closed state
ySKI2arch
ySKI2cat
S. cerevisiae (PDB:5MC6)
E
IRES
mRNA
tRNA
60S
40S
SKI
H. sapiens
S. cerevisiae

Figure S3

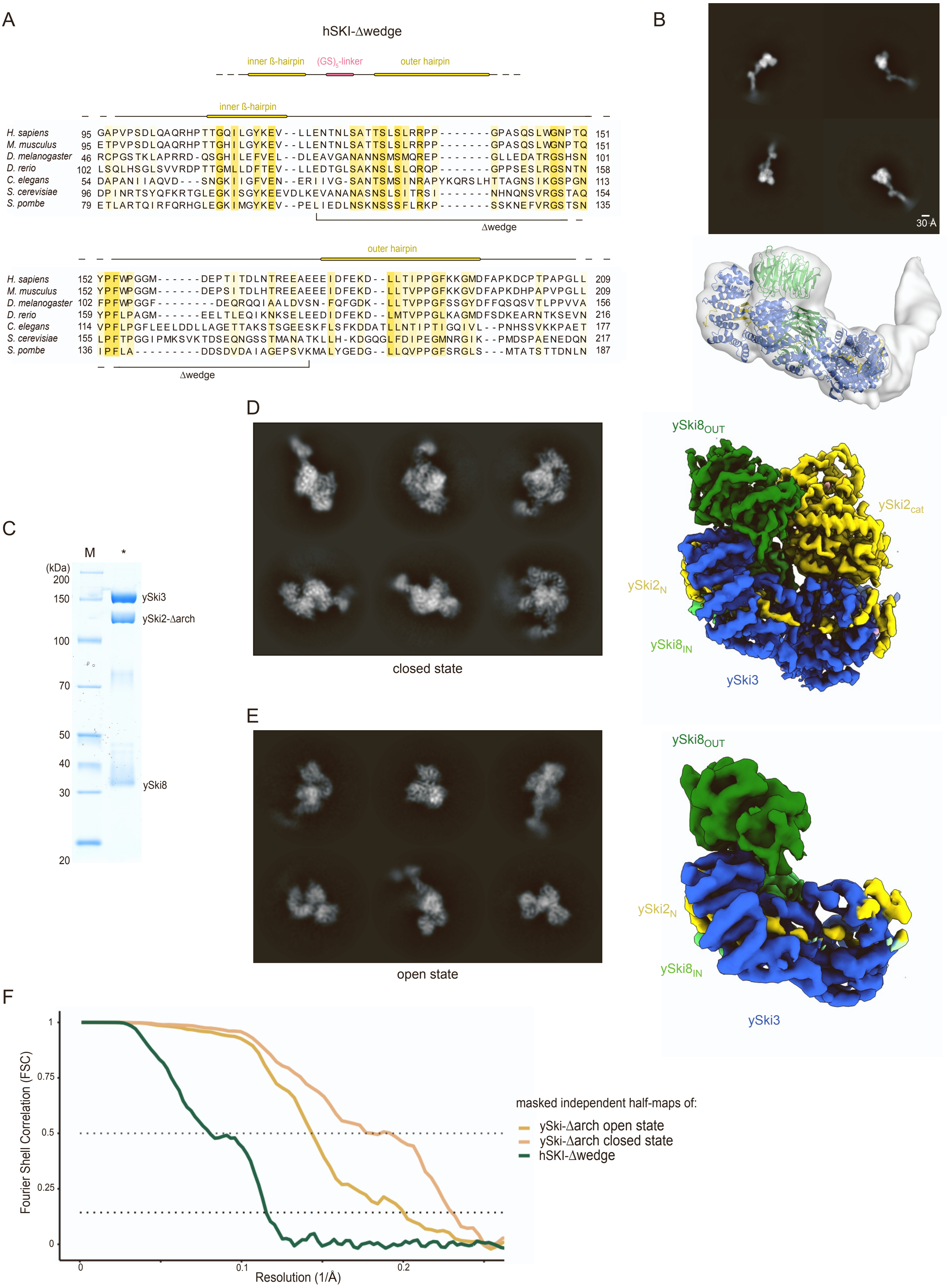

A
hSKI-Δwedge
inner ß-hairpin
(GS)5-linker
outer hairpin
inner ß-hairpin
H. sapiens 95 GAPVPSDLQAQRHPTTGQILGYKEV--LLENTNLSATTSLSLRRPP-------GPASQSLWGNPTQ 151
M. musculus 95 ETPVPSDLQAQRHPTTGHILGYKEV--LLENTNLSATTSLSLRRPP-------GPASQSLWGNPTQ 151
D. melanogaster 46 RCPGSTKLAPRRD-QSGHILEFVEL--DLEAVGANANNSMSMQREP-------GLLEDATRGSHSN 101
D. rerio 102 LSQLHSGLSVVRDPTTGMLLDFTEV--LLEDTGLSAKNSLSLQRQP-------GPPSESLRGSNTN 158
C. elegans 54 DAPANIIAQVD---SNGKIIGFVEN--ERIIVG-SANTSMSINRAPYKQRSLHTTAGNSIKGSPGN 113
S. cerevisiae 96 DPINRTSYQFKRTGLEGKISGYKEEVDLKEVANANASNSLSITRSI-------NHNQNSVRGSTAQ 154
S. pombe 79 ETLARTQIRFQRHGLEGKIMGYKEV--PELIEDLNSKNSSSFLRKP-------SSKNEFVRGSTSN 135
Δwedge
outer hairpin
H. sapiens 152 YPFWPGGM------DEPTITDLNTREEAEEEIDFEKD--LLTIPPGFKKGMDFAPKDCPTPAPGLL 209
M. musculus 152 YPFWPGGM------DEPSITDLHTREEAEEEIDFEKD--LLTVPPGFKKGVDFAPKDHPAPVPGLL 209
D. melanogaster 102 FPFWPGGF--------DEQRQQIAALDVSN-FQFGDK--LLTVPPGFSSGYDFFQSQSVTLPPVVA 156
D. rerio 159 YPFLPAGM------EELTLEQIKNKSELEEDIDFEKD--LMTVPPGLKAGMDFSDKEARNTKSEVN 216
C. elegans 114 VPFLPGFLEELDDLLAGETTAKSTSGEESKFLSFKDDATLLNTIPTIGQIVL--PNHSSVKKPAET 177
S. cerevisiae 155 LPFTPGGIPMKSVKTDSEQNGSSTMANATKLLH-KDGQGLFDIPEGMNRGIK--PMDSPAENEDQN 217
S. pombe 136 IPFLA---------DDSDVDAIAGEPSVKMALYGEDG--LLQVPPGFSRGLS---MTATSTTDNLN 187
Δwedge
B
30 Å
C
M
*
(kDa)
200
150
100
70
50
40
30
20
ySki3
ySki2-Δarch
ySki8
D
closed state
ySki8OUT
ySki2cat
ySki2N
ySki8IN
ySki3
E
open state
ySki8OUT
ySki2N
ySki8IN
ySki3
F
Fourier Shell Correlation (FSC)
1
0.75
0.5
0.25
0
0
0.1
0.2
Resolution (1/Å)
masked independent half-maps of:
ySki-Δarch open state
ySki-Δarch closed state
hSKI-Δwedge

Figure S4

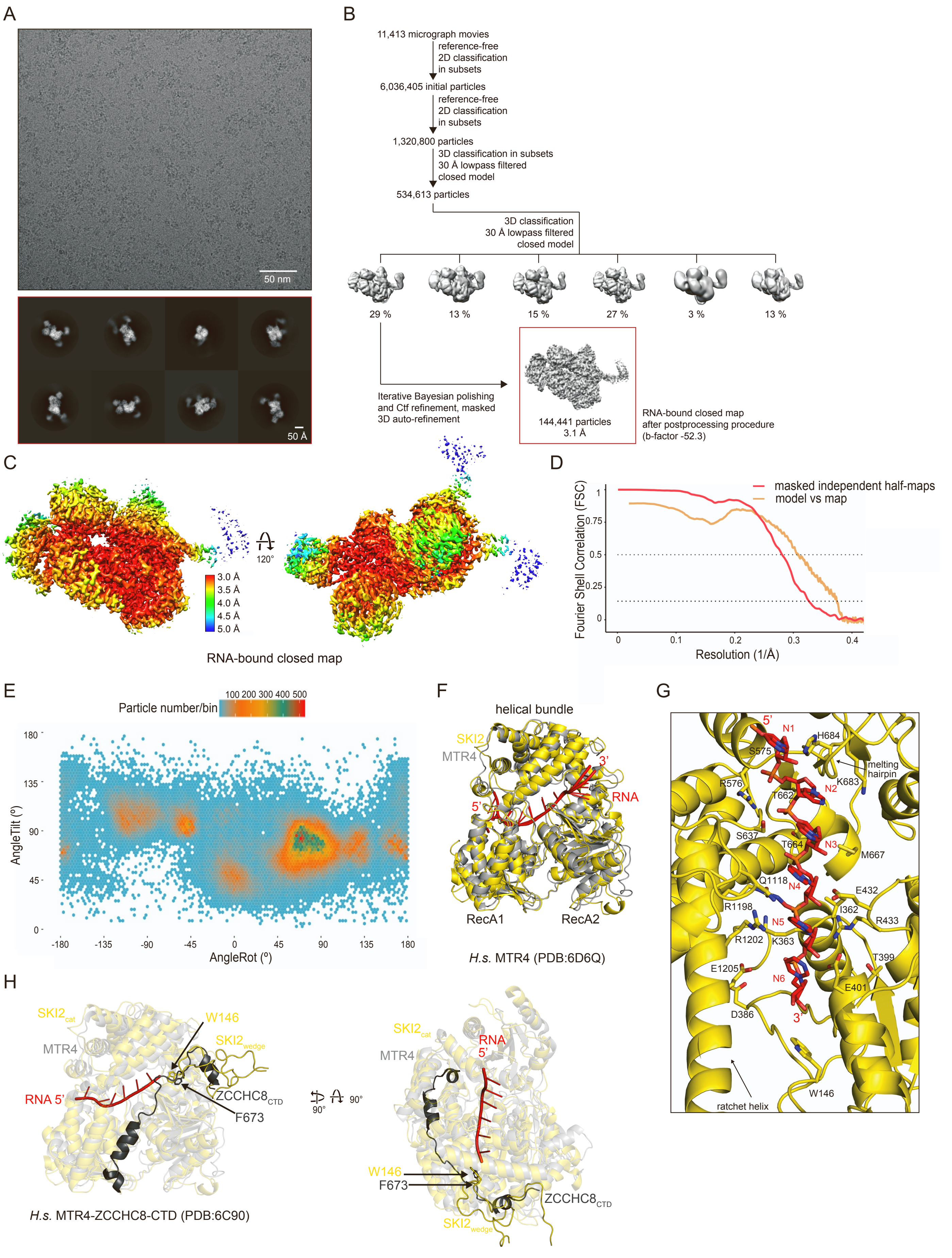

A
50 nm
50 Å
B
11,413 micrograph movies
reference-free 2D classification in subsets
6,036,405 initial particles
reference-free 2D classification in subsets
1,320,800 particles
3D classification in subsets
30 Å lowpass filtered closed model
534,613 particles
3D classification
30 Å lowpass filtered closed model
29 %
13 %
15 %
27 %
3 %
13 %
Iterative Bayesian polishing and Ctf refinement, masked 3D auto-refinement
144,441 particles
3.1 Å
RNA-bound closed map after postprocessing procedure (b-factor -52.3)
C
120°
3.0 Å
3.5 Å
4.0 Å
4.5 Å
5.0 Å
RNA-bound closed map
D
masked independent half-maps
model vs map
Fourier Shell Correlation (FSC)
Resolution (1/Å)
E
Particle number/bin
100 200 300 400 500
AngleTilt (°)
AngleRot (°)
F
helical bundle
SKI2
MTR4
RNA
RecA1
RecA2
H.s. MTR4 (PDB:6D6Q)
G
5'
N1
H684
S575
melting hairpin
K683
R576
N2
T662
S637
T664
N3
M667
Q1118
N4
E432
R1198
I362
R433
N5
R1202
K363
T399
E1205
N6
E401
D386
3'
ratchet helix
W146
H
SKI2cat
MTR4
W146
SKI2wedge
RNA 5'
ZCCHC8CTD
F673
90°
H.s. MTR4-ZCCHC8-CTD (PDB:6C90)

Figure S5

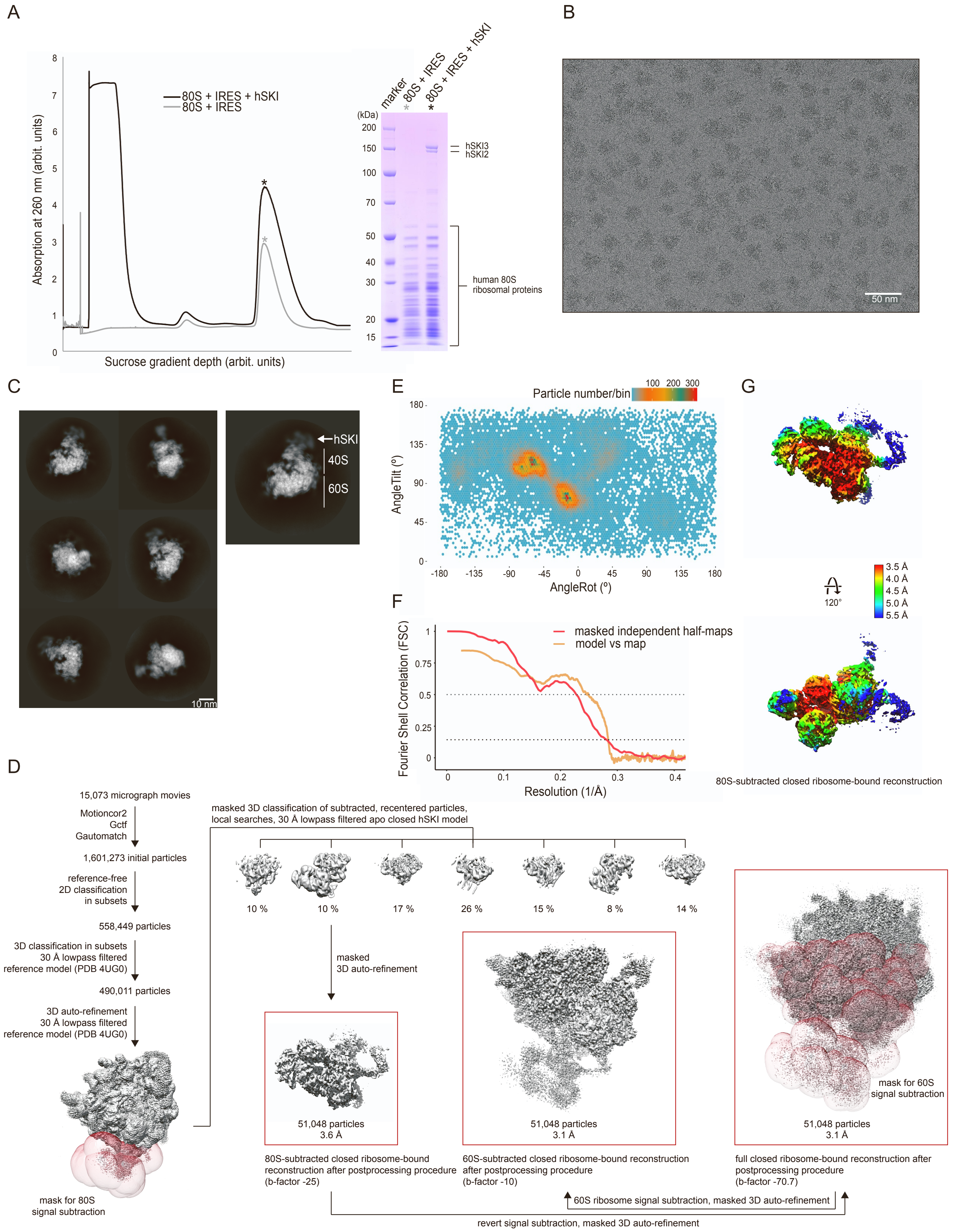
A
B
C
D
E
F
G
80S + IRES + hSKI
80S + IRES
Absorption at 260 nm (arbit. units)
Sucrose gradient depth (arbit. units)
marker
80S + IRES
80S + IRES + hSKI
(kDa)
hSKI3
hSKI2
human 80S
ribosomal proteins
50 nm
hSKI
40S
60S
10 nm
Particle number/bin
AngleTilt (°)
AngleRot (°)
Fourier Shell Correlation (FSC)
Resolution (1/Å)
masked independent half-maps
model vs map
3.5 Å
4.0 Å
4.5 Å
5.0 Å
5.5 Å
120°
80S-subtracted closed ribosome-bound reconstruction
15,073 micrograph movies
Motioncor2
Gctf
Gautomatch
1,601,273 initial particles
reference-free
2D classification
in subsets
558,449 particles
3D classification in subsets
30 Å lowpass filtered
reference model (PDB 4UG0)
490,011 particles
3D auto-refinement
30 Å lowpass filtered
reference model (PDB 4UG0)
mask for 80S
signal subtraction
masked 3D classification of subtracted, recentered particles,
local searches, 30 Å lowpass filtered apo closed hSKI model
10 %
10 %
17 %
26 %
15 %
8 %
14 %
masked
3D auto-refinement
51,048 particles
3.6 Å
80S-subtracted closed ribosome-bound
reconstruction after postprocessing procedure
(b-factor -25)
51,048 particles
3.1 Å
60S-subtracted closed ribosome-bound reconstruction
after postprocessing procedure
(b-factor -10)
mask for 60S
signal subtraction
51,048 particles
3.1 Å
full closed ribosome-bound reconstruction after
postprocessing procedure
(b-factor -70.7)
60S ribosome signal subtraction, masked 3D auto-refinement
revert signal subtraction, masked 3D auto-refinement

Figure S6

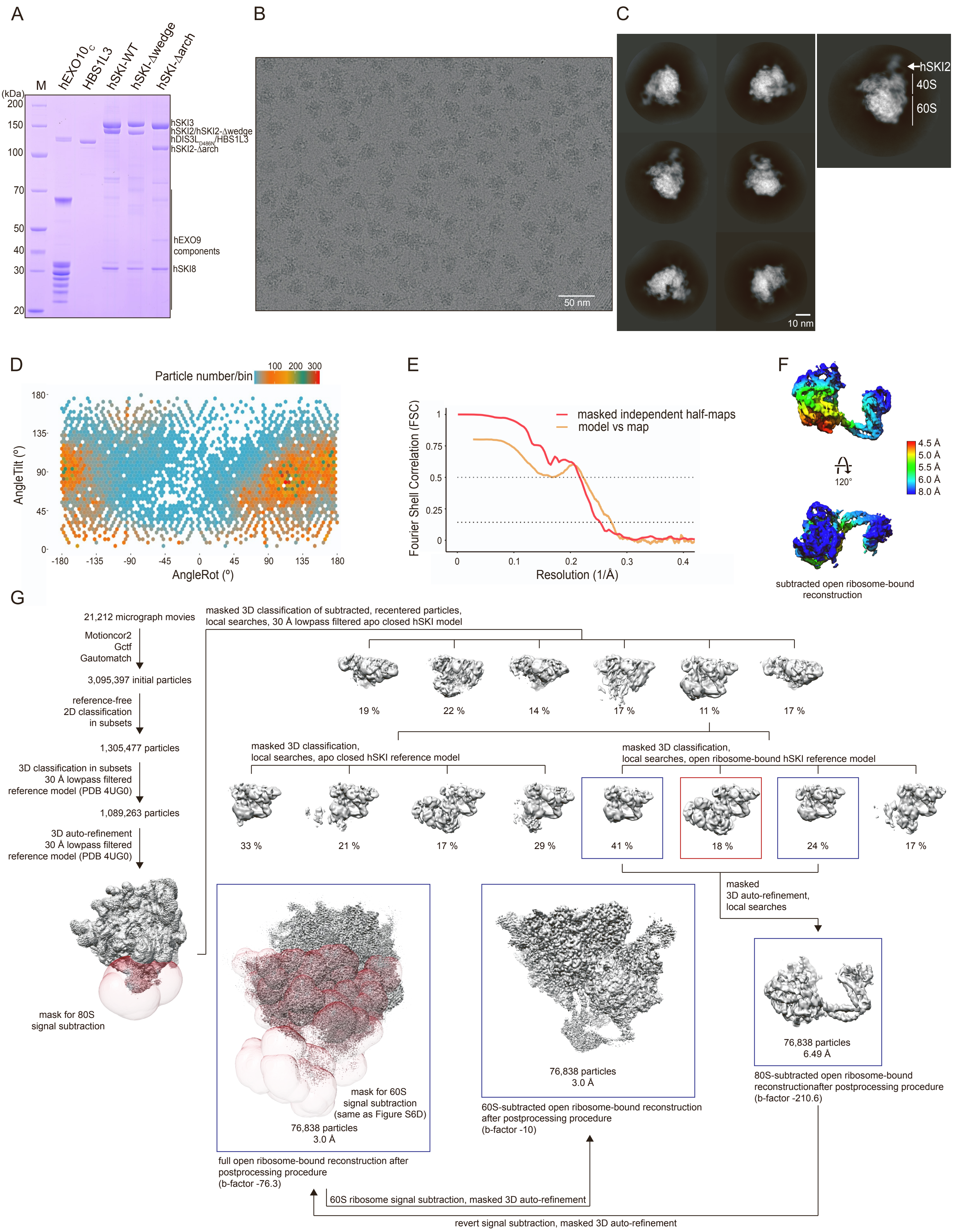
A
(kDa)
M
hEXO10C
HBS1L3
hSKI-WT
hSKI-Δwedge
hSKI-Δarch
200
150
100
70
50
40
30
20
hSKI3
hSKI2/hSKI2-Δwedge
hDIS3L D486N/HBS1L3
hSKI2-Δarch
hEXO9 components
hSKI8
B
50 nm
C
hSKI2
40S
60S
10 nm
D
Particle number/bin
100 200 300
AngleTilt (°)
AngleRot (°)
E
Fourier Shell Correlation (FSC)
Resolution (1/Å)
masked independent half-maps
model vs map
F
120°
4.5 Å
5.0 Å
5.5 Å
6.0 Å
8.0 Å
subtracted open ribosome-bound reconstruction
G
21,212 micrograph movies
Motioncor2
Gctf
Gautomatch
3,095,397 initial particles
reference-free 2D classification in subsets
1,305,477 particles
3D classification in subsets 30 Å lowpass filtered reference model (PDB 4UG0)
1,089,263 particles
3D auto-refinement 30 Å lowpass filtered reference model (PDB 4UG0)
mask for 80S signal subtraction
masked 3D classification of subtracted, recentered particles, local searches, 30 Å lowpass filtered apo closed hSKI model
19 %
22 %
14 %
17 %
11 %
17 %
masked 3D classification, local searches, apo closed hSKI reference model
33 %
21 %
17 %
29 %
masked 3D classification, local searches, open ribosome-bound hSKI reference model
41 %
18 %
24 %
17 %
masked 3D auto-refinement, local searches
mask for 60S signal subtraction (same as Figure S6D)
76,838 particles
3.0 Å
full open ribosome-bound reconstruction after postprocessing procedure (b-factor -76.3)
76,838 particles
3.0 Å
60S-subtracted open ribosome-bound reconstruction after postprocessing procedure (b-factor -10)
76,838 particles
6.49 Å
80S-subtracted open ribosome-bound reconstructionafter postprocessing procedure (b-factor -210.6)
60S ribosome signal subtraction, masked 3D auto-refinement
revert signal subtraction, masked 3D auto-refinement

Figure S7

Table S1. Cryo electron microscopy data collection summary, processing statistics and model quality indicators (related to Figures 1, 4, 5 and 6)

**Cryo electron microscopy data collection**					
Microscope	FEI Titan Krios GII				
Voltage (kV)	300				
Camera	Gatan K3-Summit				
Energy Filter	Gatan Quantum-LS (GIF)				
Pixel size (Å/pix)	1.094		0.851		
Preset target global defocus range (μm)	0.6 - 2.4				
**3D reconstruction**	**apo closed *H.s.* SKI**	**apo open *H.s.* SKI**	**RNA bound closed *H.s.* SKI**	**ribosome bound closed *H.s.* SKI**	**ribosome bound open *H.s.* SKI**
Number of movies	11,079		11,413	15,073	21,212
Exposure per sec ($e^-/Å^2/s$) /total exposure ($e^-/Å^2$)	11.86/47.44		22.77/68.31	11.16/55.8	11.27/67.62
Number of frames/movie	40		30	35	40
Initially selected particle candidates	4,400,241		6,036,405	1,601,273	3,095,397
Final number of particles	84,591	154,135	144,441	51,048	76,838
Resolution $_{\text{FSC independent halfmaps}}$ (Å)[a]	3.7	3.8	3.1	3.6	6.5
Local resolution range (Å)	3.3-15.8	3.4-13.1	2.8-12.6	3.1-15.6	4.6-10.7
Sharpening B-factor determined/applied ($Å^2$)	-88.1/-88.1	-145.3/-145.3	-52.3/-52.3	-73.6/-25	-210.6/-210.6
EMDB accession number	EMD-13923	EMD-13925	EMD-13927	EMD-13928	EMD-13929

Table S1 - continued.

**Refinement**	**apo closed *H.s.* SKI**	**apo open *H.s.* SKI**	**RNA bound closed *H.s.* SKI**	**ribosome bound closed *H.s.* SKI**	**ribosome bound open *H.s.* SKI**
PDB code	7QDR	7QDS	7QDY	7QDZ	7QE0
No atoms	22,827	13,798	22,933	22,598	7,619
Residues (protein)	2,991	1,771	2,991	2,924	949
Residues (RNA)	-	-	6	6	9
CC_{box}, CC_{mask}, CC_{volume}[b]	0.81, 0.86, 0.85	0.80, 0.82, 0.81	0.77, 0.80, 0.77	0.86, 0.81, 0.79	0.87, 0.74, 0.73
Resolution $_{\text{FSC map vs. model@0.143}}$ (Å)[b]	3.6	3.3	2.7	3.0	4.0
r.m.s. deviations					
Bond lengths (Å)	0.006	0.005	0.009	0.007	0.004
Bond angles (°)	0.819	0.852	1.101	0.969	0.998
Ramachandran favored (%)	94.25	94.32	94.39	95.05	96.19
Ramachandran allowed (%)	5.75	5.68	5.61	4.95	3.81
Ramachandran disallowed (%)	0.00	0.00	0.00	0.00	0.00
MolProbity score	1.99	2.04	1.96	2.09	2.07
Clash score	12.26	14.02	11.49	17.6	20.94

[a]according to the Fourier Shell Correlation (FSC) cut-off criterion of 0.143 defined in (Rosenthal and Henderson, 2003)
[b]according to the map-vs.-model Correlation Coefficient definitions in (Afonine et al., 2018)

Table S2. Kinetic parameters of hSKI ATP hydrolysis derived according to Michaelis-Menten (related to Figure 4A)

	hSKI-WT	hSKI-Δarch	hSKI-Δwedge	hSKI-WT (no RNA)	hSKI-DEAD*	hSKI-THES
V_{max} (μM/s)	0.03427	0.04478	0.02990	0.002387	N/A	0.0009766
K_m (μM)	149.4	121.5	190.5	582.2	N/A	-2.574
k_{cat} (s^{-1})	0.6853	0.8957	0.5981	0.04775	N/A	0.01953
R^2	0.9817	0.9871	0.9822	0.2016	N/A	0.1477

*The hSKI-DEAD data could not be reconciled with the Michaelis-Menten equation. No kinetic parameters were derived (N/A, not applicable).
